# Graphene and other 2D materials: a multidisciplinary analysis to uncover the hidden potential as cancer theranostics

**DOI:** 10.7150/thno.40068

**Published:** 2020-04-07

**Authors:** Laura Fusco, Arianna Gazzi, Guotao Peng, Yuyoung Shin, Sandra Vranic, Davide Bedognetti, Flavia Vitale, Acelya Yilmazer, Xinliang Feng, Bengt Fadeel, Cinzia Casiraghi, Lucia Gemma Delogu

**Affiliations:** 1 Department of Chemical and Pharmaceutical Sciences, University of Trieste, Trieste, Italy.; 2 Fondazione Istituto di Ricerca Pediatrica, Città della Speranza, Padua, Italy.; 3 Cancer Program, Sidra Medicine, Doha, Qatar.; 4 Institute of Environmental Medicine, Karolinska Institutet, Stockholm, Sweden.; 5 Department of Chemistry, University of Manchester, Manchester, UK.; 6 Nanomedicine Lab, Faculty of Biology, Medicine and Health, University of Manchester, Manchester, UK.; 7 Department of Neurology, Bioengineering, Physical Medicine & Rehabilitation, Center for Neuroengineering and Therapeutics, University of Pennsylvania, Philadelphia, USA; Center for Neurotrauma, Neurodegeneration, and Restoration, Corporal Michael J. Crescenz Veterans Affairs Medical Center, Philadelphia, USA.; 8 Department of Biomedical Engineering, Ankara University, Ankara, Turkey.; 9 Stem Cell Institute, Ankara University, Ankara, Turkey.; 10 Faculty of Chemistry and Food Chemistry, School of Science, Technische Universität Dresden, Dresden, Germany.; 11 Department of Biomedical Sciences, University of Padua, Padua, Italy.

**Keywords:** 2D materials, cancer theranostics, future perspectives, graphene, nanomedicine.

## Abstract

Cancer represents one of the main causes of death in the world; hence the development of more specific approaches for its diagnosis and treatment is urgently needed in clinical practice. Here we aim at providing a comprehensive review on the use of 2-dimensional materials (2DMs) in cancer theranostics. In particular, we focus on graphene-related materials (GRMs), graphene hybrids, and graphdiyne (GDY), as well as other emerging 2DMs, such as MXene, tungsten disulfide (WS2), molybdenum disulfide (MoS2), hexagonal boron nitride (h-BN), black phosphorus (BP), silicene, antimonene (AM), germanene, biotite (black mica), metal organic frameworks (MOFs), and others. The results reported in the scientific literature in the last ten years (>200 papers) are dissected here with respect to the wide variety of combinations of imaging methodologies and therapeutic approaches, including drug/gene delivery, photothermal/photodynamic therapy, sonodynamic therapy, and immunotherapy. We provide a unique multidisciplinary approach in discussing the literature, which also includes a detailed section on the characterization methods used to analyze the material properties, highlighting the merits and limitations of the different approaches. The aim of this review is to show the strong potential of 2DMs for use as cancer theranostics, as well as to highlight issues that prevent the clinical translation of these materials. Overall, we hope to shed light on the hidden potential of the vast panorama of new and emerging 2DMs as clinical cancer theranostics.

## 1. Introduction

Graphene, a single layer of graphite, is undoubtedly the most famous 2-dimensional material (2DM), due to its outstanding properties that can be exploited in various applications, ranging from electronics to composites and biomedical applications 1-3. Moreover, an entire family of graphene-related materials (GRMs), each with its own properties, is available - ranging from chemical derivatives of graphene, such as graphene oxide (GO), nano-graphene oxide (NGO), and reduced graphene oxide (rGO), to new 2DMs, such as graphdiyne (GDY). Furthermore, graphene hybrids can be easily obtained by combining single or few-layer graphene (FLG) or other GRMs with different types of nanomaterials (e.g., quantum dots (QDs), metallic and semiconducting nanomaterials, etc.). Although graphene quantum dots (GQDs) are not necessarily 2D, they have been used for biomedical applications, thus these are also included in this review for sake of completeness. In addition, the family of 2DMs is very large, and other materials have started to be used in nanomedicine, for instance the family of transition metal dichalcogenides (TMDs), such as tungstenum disulphide (WS_2_) and molybdenum disulfide (MoS_2_), hexagonal boron nitride (h-BN), nitrides and carbonitrides (MXenes), black phosphorus (BP) nanosheets, silicene, antimonene (AM), germanene, biotite, metal organic frameworks (MOF), and layered double hydroxide (LDH).

One of the main goals in modern oncology overlaps with that of nanomedicine: the development of alternative approaches through the combination of two or more forms of treatments and diagnostic techniques. To achieve this aim, advanced nanomaterials are extensively considered as they can be used as imaging components for cancer detection/visualization with customized therapeutic agents, as well as vectors for controlled-release mechanisms and targeting strategies, making nanomaterials promising nanotheranostic tools 4.

According to the traditional definition, a theranostic agent consists of a therapeutic and imaging (aimed at diagnosis) component combined within a single formulation. A more recent view expanded the definition of theranostics by including tools where imaging is performed to aid or guide the therapy and not necessarily to perform a diagnosis 5-9. Theranostic nanomedicines offer the opportunity to combine into a single nanoplatform multiple imaging methodologies and therapeutic functions, such as passive and active targeting for cancer therapy (theranostics for drug delivery), and stimuli-responsive drug release (e.g., theranostics for temperature- and pH-dependent therapy) 10.

In general, 2DMs shows outstanding properties, such as remarkable light-weightness and flexibility, high surface-to-volume ratio, near-infrared (NIR) light absorption, and characteristic Raman spectrum, which make them very attractive from a biological perspective. Furthermore, these materials are suitable for multiple functionalizations, which is fundamental for the treatment and diagnosis of cancer, or both (theranostics), as we will discuss in more detail in this review.

In the present review, the state-of-the-art of graphene and 2DMs in cancer theranostics is presented by depicting the four main scenarios of nano-based approaches in cancer, namely: i) imaging methods, ii) drug and gene delivery, iii) photothermal therapy (PTT), and iv) photodynamic therapy (PDT).

**i) Imaging in cancer.** Cancer imaging is indispensable not only to enable the early detection of cancer, but also to determine the precise tumor location and stage, as well as to direct therapy and check for cancer recurrence after the treatment. Various imaging techniques, including positron emission tomography (PET), X-ray computed tomography (CT), magnetic resonance imaging (MRI) and ultrasonography, are available for clinical applications, while other methodologies that are attracting the interest of the biomedical research community, such as fluorescence molecular tomography (FMT), are still at the preclinical stage 11-14. Since each technology has its unique strengths and limitations, hybrid imaging platforms, such as PET-CT, FMT-CT, FMT-MRI, and PET-MRI are being developed to improve data reconstruction and visualization. New nanotechnology-based agents may be beneficial when it comes to proposing new imaging tools that will help overcome the common issues of current agents (e.g., toxicity, such as the one related to X-ray contrast agent, and lack of specificity, common to both X-ray and MRI contrast agents, and increased hypersensitivity following chemotherapy). Some 2DMs show strong light-matter interaction effects, such as fluorescence, making them an ideal base to build experimental theranostic tools 15. Moreover, the 2-dimensionality enables easy load of specific nanomaterials and molecules, which provide complementary properties to the selected 2DMs as a vector (e.g., electrochemical properties, magnetic functions, fluorescence, radioactivity, etc.), allowing to enhance and expand their potential use in oncology for imaging and diagnosis.

**ii) Drug and gene delivery.** Since 1995, when Doxil entered into the clinic for the delivery of the potent aromatic anticancer agent doxorubicin (DOX), several nanotools have been approved for drug delivery, and the list of nanodrugs in clinical trials is still growing 16. The ability to multifunctionalize nanomaterials and nanoparticles (NPs), the high drug loading capacity, the possibility to reduce the drug exposure in the undesired tissue, and the improved drug solubility represent the main advantages of using nanosystems in cancer treatment. None of the cancer nanotherapeutics approved so far, mainly based on liposomal, silicon, gold, and iron particles, possess the same physical/chemical properties of graphene or other 2DMs 17,18. Systems can be designed to localize at the target disease site, which would allow the early detection of tumors. An ideal material, in this case, may be able to achieve molecular targeting that can be imaged during its circulation in the body. Upon reaching its destination, it may have targeting moieties that associate with cell-surface receptors, internalize into the cytosol, reach an intracellular target if necessary, and release the active therapeutic 7,18,19. The effectiveness of a drug conjugate is related to its ability to improve therapeutic index relative to free drug, generally by reducing toxicity and enhancing efficacy. Historically, a panel of nanosystems, including liposomes, metal NPs, and carbon-based materials, were modified via non-covalent or covalent modification in order to deliver chemotherapeutic agents, such as DOX and paclitaxel, or agents for gene therapy (e.g., short interfering RNA, siRNA) 20-23, in combination with imaging agents (encapsulated or intrinsic) to the tumor site 24-26.

**iii) Photothermal therapy (PTT)** is a type of localized treatment that relies on the presence of an optical absorbing agent, also known as photosensitizer (PS), which can absorb energy and convert it into heat upon stimulation by an electromagnetic radiation, such as radiofrequency, microwaves or NIR irradiation 27. When compared to conventional radiotherapy or chemotherapy 28, the key advantages of PTT include the capability of deep tissue penetration and minimal nonselective cell death in the surrounding healthy tissue 27. In the biological environment, overheating of the PS may lead to hyperthermia that may cause several hazardous effects, such as protein denaturation and aggregation, evaporation of cytosol, cell lysis, apoptosis, and necrosis. An ideal PS should possess a high selectivity towards the target tissue, together with large absorption cross-sections for optical wavelengths, low toxicity, easy functionalization capability, and high solubility in biocompatible solutions 29. PTT has been widely used in nanomedicine alone or in combination with other therapies and imaging modalities, such as gene therapy 30 and photoacoustic imaging (PAI) 31, and many are the types of nanomaterials investigated in this direction such as gold nanoparticles and PEGylated silica-cored Au nanoshells, which are the first photothermal nanoparticles that have advanced into clinical trials.

**iv) Photodynamic therapy (PDT)** is now well established as a clinical treatment modality for various diseases, including cancer, and particularly for the treatment of superficial tumors (e.g., esophagus, bladder, and melanoma) 32,33. Similar to PTT, also PDT mechanism relies on the presence of a PS; however, in this case, its activation is obtained through the illumination with visible light, instead of the local heat application. The combined action of the excited triplet photosensitizer and molecular oxygen results in the formation of singlet oxygen (^1^O_2_), which is thought to be the main mediator of cellular death induced by PDT.

To date, the most studied nanotheranostics for PTT and PDT involve organic NPs, such as polymeric NPs or liposomes and inorganic NPs including quantum dots (QDs) and silica NPs. Owing to their unique properties of high optical absorption capacity in the NIR region and photothermal conversion, carbon-based nanomaterials represent ideal candidates for PTT 34-37. The ability of some 2DMs to respond to light is exploited in optical therapies, including PTT and PDT. Finally, thanks to the adsorption onto their surface of specific molecules and NPs, these nanotools can be endowed with additional magnetic, radioactive, or electrochemical properties.

## 2. Further considerations before exploring the potential of 2DMs as cancer theranostics

Based on the needs in oncology, and the available imaging techniques and innovative therapeutic treatments, the 2DMs family should demonstrate a list of properties *before* being introduced into the clinic:

a) Lack of toxicity/acceptable biocompatibility (i.e., are the 2DMs able to escape immune recognition?);

b) Selective toxicity to cancer cells (i.e., are 2DMs selective to all cancer cells or only specific types?) and personalized medicine (i.e., are different 2DMs needed for different patients?);

c) Appropriate biodistribution (e.g., in relation to the type of route of injection, are 2DMs degraded and/or excreted?);

d) When designed for drug/gene delivery, 2DMs must be able to protect the conjugated drugs from degradation, facilitate their solubilization, sustain their release, and selectively target cancer cells;

e) When designed for PTT, 2DMs must be stable and possess large absorption cross-sections at the specific excitation wavelengths;

f) When designed for PDT, the 2DM must be able to act as a PS, being activated by light of a specific wavelength and producing a form of singlet oxygen that kills nearby cells.

In addition to the above points, 2DMs performance in different application must also be benchmarked against other particles and materials in clinical trials (are they capable of competing with other particles already at the stage of clinical trials?).

In the present review, the theranostic use of 2DMs is dissected from a different perspective compared to the recent reviews published on this topic so far 9,38-46. First, we discuss the studies carried out, from an oncological and biological point of view, highlighting the multifunctional complexity of the developed 2DMs in terms of different types of imaging approaches, therapeutic techniques, conjugated drugs/targeting moieties, as well as the relative targeted cancer. Our review also describes the material characterizations performed - as the properties of 2DMs change with functionalization and processing, a comprehensive characterization is necessary to ensure reproducibility and, therefore, envisage the use of 2DMs in nanomedicine. The first part of this review focuses on the most used 2DMs (i.e., graphene, and its chemical derivatives and hybrids) 47, and the multitude of characteristics exploited for therapy and diagnosis of cancer, highlighting multiple combined purposes: imaging, drug/gene delivery, PTT, and PDT. In contrast to previous reviews, a separate section is dedicated to 2DMs beyond graphene: each material is briefly introduced and theranostic works discussed taking into account of the design strategy, the type of cancer investigated, the working biological mechanisms (when reported), the model used, the techniques, and the resulting outcomes. We include also emerging 2DMs that did not prove their potentialities as theranostic tools yet, but have promising characteristics for their future development in this direction. Moreover, the present review provides an overview and a critical discussion on the characterization performed for each study reported. In addition, tables and graphics were generated to indicate the types of cancer investigated in relation to the different approaches, the nanodrugs, and the forms of imaging. Furthermore, schematic views to compare the materials are also specified. The information collected in this review will allow the readers to navigate among the 2DMs proposed for use in cancer theranostics, discriminate which material is more suitable for a specific theranostic aim, and understand the oncological gaps and the remaining open questions in 2D cancer theranostics.

## 3. Literature review: looking back at ten years of 2DMs theranostic research

A systematic and critical review of the literature on graphene, GRMs, graphene hybrids, and new emerging 2DMs, studied in biomedicine as nanotools for cancer theranostic applications, was performed according to the Preferred Reporting Items for Systematic Reviews and Meta-Analyses (PRISMA) guidelines.

The search has been carried out using different electronic databases as data sources (PubMed, Scopus, and ToxLine); the following predetermined keywords, related with the selected nanomaterials and their application in cancer theranostics, were used in several different combinations: graphene, GRMs, GO, rGO, GQDs, NGO, GDY, MXene, WS_2,_ MoS_2_, hBN, BP, silicene, AM, germanene, biotite, MOF or LDH and theranostics, 2DMs, drug delivery and cancer, gene delivery and cancer, imaging and cancer, PTT and cancer, PDT and cancer, cancer diagnosis, cancer therapy, theranostic nanomaterials, theranostic nanosystems, or theranostic nanoplatforms. As an additional tool, the research was extended by consulting the literature of relevant reviews and included studies in the field of nanotechnologies and theranostics. The list of reported studies includes all the retrieved publications from 2008 to July 2019. The adopted inclusion criteria were: (1) studies published in English; (2) full-text articles; (3) cancer as target disease; (4) the presence of at least one type of GRM or new emerging 2DMs in the considered nanosystem structure; (5) studies with at least one diagnostic method and one therapeutic strategy; (6) at least one of these strategies was due to the presence of GRMs or new emerging 2DMs; (7) *in vitro* or *in vivo* studies in appropriate animal models. The study selection required an initial stage in which the articles were selected, according to the eligibility criteria, based on their title, abstract, and keywords. In the second stage, the authors considered the full text of all the eligible studies, stating whether these met the eligibility criteria. Some of the first studies found in the literature were claiming possible theranostic applications, but only one application was experimentally demonstrated; these studies were excluded by the present review. According to these criteria, the selected works for discussion on graphene, GRMs, and graphene hybrids for imaging combined with gene/drug delivery, PTT/PDT, or gene/drug delivery in association with PTT/PDT are reported in **Table 1**, **Table 2** and** Table 3**, respectively;** Table 4** reports the published studies for the new 2DMs.

### 3.1. Graphene-related materials and hybrid nanosystems for cancer theranostics

The introduction of graphene and GRMs in the field of theranostics has allowed the combination of effective therapeutic procedures (e.g., relying on targeted drug/gene delivery, PTT, PDT, etc.) with a wide range of different imaging methods, such as MRI, PET, CT, fluorescent imaging, PAI, and photothermal imaging. The multiple functions of these materials arise from their intrinsic physicochemical properties and the possibility of conjugating them with a wide variety of molecules 48. We here discuss the different approaches used, starting from less complex nanotools, with only two applications (e.g., imaging and gene/drug delivery), to more and more complex theranostic tools with several multiple functions, where imaging, gene/drug delivery, and PTT/PDT are combined in a unique nanotheranostic platform. All the works taken into consideration are reported in **Table 1** (imaging and drug/gene delivery),** Table 2** (imaging and PTT/PDT) and** Table 3** (imaging and drug/gene delivery in association with PTT/PDT).

#### 3.1.1. Imaging and drug/gene delivery

Ten years ago, Sun* et al*. introduced the use of a GRM in cancer theranostics, exploring a single-layer NGO-based platform for combined diagnosis and therapy 49. The material, showing intrinsic photoluminescence (PL) exploitable for live cell imaging in the NIR, was coated by polyethylene glycol (PEG) to improve its solubility and biocompatibility. Moreover, NGO imaging properties were combined with its loading capability, resulting in a theranostic nanoplatform. To this end, the anticancer drug DOX was bound to NGO sheets, which were functionalized with the B cell specific antibody Rituxan (anti-CD20) for the *in vitro* selective binding and killing of Burkitt's lymphoma cells. A sustained Raji B cell growth inhibition (∼80%) was observed after 2-h incubation with the nanosystem at DOX concentration of 10 µmol/L followed by 48-h incubation in fresh cell medium.

This promising work paved the way for the subsequent explosion of graphene-related research for cancer theranostics. Several studies further involved the chemical functionalization of graphene, GRMs, and graphene hybrids to improve and expand their use for gene/drug delivery and imaging 49-77. In particular, DOX represented the first-choice chemotherapeutic agent also for other authors. For example, GO was functionalized by magnetic/fluorescent SiO_2_ microsphere through an amidation process and loaded with DOX, creating an active fluorescent magnetic drug carrier and a potential optical imaging tool 75. Similarly, Ma and co-authors successfully used Au-decorated GO NPs for combined DOX delivery and intracellular Raman imaging for cervical cancer (HeLa cells) 69,78. Thanks to the quenching of DOX fluorescence induced by the attachment to the GO-nanosystem, it was possible to track the delivery of the drug that was able to emit only when released in the tumor cells. Theranostic GO-based DOX nanocarriers were also fabricated by Nie* et al*. for combined drug delivery and PAI in lung cancer cells (H1975 cells) and *in vivo*. The system was also linked to the Cy5.5 dye to allow fluorescence imaging. Moreover, thanks to the high loading capacity, the material was able to induce effective PAI-monitored chemotherapy in mice 65.

Different studies have succeeded in exploiting GQDs fluorescence for targeted cancer imaging and, at the same time, for tracking and monitoring drug delivery processes and cancer therapies. For example, GQDs have proven to be an optimal multifunctional nanovehicle for delivering DOX to targeted cancer cells, enabling the monitoring of the intracellular anticancer drug release as a dual-fluorescent nanoprobe 54,56,60-62,66,68,79. It has been underlined how the drug-loaded stereochemistry can affect GQD imaging behavior 66. In this case, anticancer drug curcumin (Cur) chelated the fluorescence of GQDs until the release into the tumor site, allowing restoring of the fluorescence of GQDs, hence acting as a bio-probe for tumor imaging. GQDs were also explored as efficient nucleic acid nanocarriers for the regulation of intracellular miRNAs and imaging 62. In this case, the multiple gene probes loaded on GQDs, showed a combined effect for the enhancement of the therapeutic efficacy. The uptake of the GQDs by HeLa cells was monitored by exploiting the intrinsic PL of GQDs, while the fluorescence of the gene probe, produced by the recognition of the target, was used to monitor the regulation of the target gene.

Paclitaxel (PTX) was also explored for drug delivery by Zang* et al*., which used indocyanine green (ICG)-loaded NGO for combined PTX shuttle at the tumor site and fluorescence imaging 58. *In vivo* data demonstrated that the system was highly biocompatible and able to induce a total cancer suppression in mice.

Moreover, graphene has attracted increasing attention in MRI-based theranostic protocols. As a non-invasive diagnostic technology, MRI has been widely used in the clinic; however, challenges associated to biocompatibility and sensitivity of the contrast agents used in MRI and in nanotechnology-based approaches still need to be solved 80. In 2013, Zhang* et al*. developed a positive T_1_ MRI GO-contrast agent ⦋GO-DTPA-Gd/DOX⦌ based on GO-gadolinium (Gd) complexes. This nanocomplex offers a dual-modality: T_1_ MRI/fluorescence imaging and drug delivery functionalities, which exhibited low cytotoxicity. The developed MRI contrast agent can be internalized into cells, enabling cellular MR imaging.

Moreover, GO-DTPA-Gd allows a high capacity DOX loading, resulting in potent anti-cancer activity against HepG2 cells 71. Li* et al*. fabricated a GO/BSA-Gd_2_O_3_/AS1411-DOX theranostic nanocomplex with BSA-Gd_2_O_3_ NPs intended to be used as an MRI contrast agent, where graphene oxide nanoplates (GONs) are used as both contrast agents and drug nanocarrier, conjugated with an aptamer, AS1411, which serves as the targeting molecule 57. This theranostic nanocomplex has shown not only an increased MR contrast signal, but also a specific targeting and growth inhibition of human renal carcinoma 786-0 cells, demonstrating drug delivery ability both *in vitro* and *in vivo*. Working in a similar direction, in 2018, Usman* et al*. developed a bimodal theranostic nanodelivery system (BIT) suitable for combined and simultaneous MRI and drug delivery 52. This nanoplatforms consisted of GO, chlorogenic acid as a chemotherapeutic agent, gadolinium (Gd), and gold nanoparticles (AuNPs) as contrast agents for MRI. The authors reported targeting and growth inhibition of hepatocellular carcinoma HepG2 cells, and the nanoplatform was shown to produce a stronger signal than the conventional MRI contrast agents (Gd(NO_3_)_3_). The obtained results portray this system as a promising future chemotherapeutic for cancer treatment with MRI diagnostic modalities 52. The same group also developed another theranostic system for MRI, the so-called GOTS system, which consisted of GO, protocatechuic acid as an anticancer agent, Gd (III) nitrate hexahydrate combined with AuNPs as a diagnostic agent. The system was able to induce cancer cell HepG2 death at a concentration of 100 µg/mL. The T_1_ weighted image of GOTS showed increment in contrast to the nanocomposite to be higher than pure (Gd(NO_3_)_3_ and water reference. These initial *in vitro* outcomes suggest an upcoming solution to the highly toxic chemotherapy and diagnosis of cancer diseases 53. In 2019, Yu Luo* et al*. developed ultrasmall and superparamagnetic iron oxide nanoparticles (IONPs), loaded on GO nanosheets [SPIONs@GO], for T1-MR imaging and pH-sensitive chemotherapy of tumors 50. They showed a sensitive and modulable pH-responsive drug release behavior triggered by even subtle pH alterations. *In vivo* results further confirmed high-resolution T_1_-weighted MR imaging performance and high antitumor efficacy 50.

Functionalization with folic acid (FA) was introduced with the aim of both improving drug selectivity and reducing the material related toxicity towards healthy cells. For examples, He* et al*. designed a GO-capped mesoporous silica nanoplatform (MSP-BA-GO) for remote-controlled drug release, combined with DOX-loading and folic acid modification 67. DOX@MSP-BA-GOF displayed a selective cellular internalization via receptor-mediated endocytosis and the subsequent release of DOX by remote illumination. In another study, Diaz-Diestra* et al*. demonstrated that the FA functionalization of rGO-based nanoplatform (FA-rGO/ZnS:Mn) improved targeting of the folate receptor-positive cancer cells and inhibited the toxicity exerted on non-tumor cells up to 72 h exposure 51. This was due to the surface passivation of FA, which allowsto decrease the strong hydrophobic interaction between cell membrane wall and graphene flakes edges or corners, which are proven to induce toxic responses. The nanosystem killing efficiency was 50% at a concentration of 3 µg/mL of DOX, far below the values frequently reported in the literature (>10 µg/mL). Chen* et al*. developed a rGO-based nanoplatform based on rGO/Au/Fe_3_O_4_ hybrids, used as cargo-filled graphene nanosacks that when reintroduced into the aqueous environment can rapidly release soluble salt cargoes. These open structures can be adaptable to a drug-controlled release form by adding a polymeric filler. To apply the theranostic use on this system, the authors combined a magnetically responsive platform, deonstrating an optimal contrast enhancement as imaging probes in both MR imaging and X-ray computed tomography 70.

One of the most promising strategies for the treatment of cancer consists of combining chemotherapy with gene therapy. In this view, in the effort of creating a single platform, able to efficiently deliver genes, drugs and contrast agents to the cancer site, Wang* et al*. reported a functionalized chitosan magnetic graphene (CMG) system for the simultaneous delivery of gene/drugs and SPIO y to tumors. *Ex vivo* MRI demonstated the use of CMG as a strong T_2_ contrast-enhancing agent. As shown by biodistribution studies and MRI, CMGs selectively accumulated in tumors. The system has been reported to show an efficient drug loading capacity, pH-dependent drug/gene release and better cytotoxicity than free DOX. Moreover, DOX-CMG-GFP-DNA NPs were able to deliver both DOX and GFP coding pDNA to the tumor site in mice, serving as an integrated system of targeted imaging, drug/gene co-delivery and real-time monitoring of therapeutic effects 73.

#### 3.1.2. Imaging and photothermal/photodynamic therapy

Compared to conventional treatment methods, PTT and PDT exert a selective and non-invasive anticancer action. The former refers to the use of electromagnetic radiation, such as NIR wavelengths, to excite a PS. The absorption of a specific band light leads the PS to an excited state. Coming back to the steady-state, it releases energy in the form of heat, leading to cancer cell photoablation. To gain this effect and to avoid nonspecific killing of healthy cells, PSs need to absorb in the NIR and be selectively uptaken into cancerous cells 81. On the other hand, in PDT, PSs absorb light and transfer the energy to the oxygen present in the surrounding tissue. The production of highly reactive oxygen species (ROS), such as ^1^O_2_ and free radicals, oxidizes cellular and sub-cellular structures, such as plasma, lysosomal, mitochondrial, and nuclear membrane, leading to non-recoverable damage to tumor cells 82.

Currently, a large number of nanomaterials are being studied for PTT and PDT applications, thanks to their optical absorbance in the NIR, including AuNPs and GRMs 73,74,83-110. From a theranostic point of view, photothermal agents (PTAs) are very appealing since they can also serve as contrast agents for PAI, representing a noninvasive imaging modality thanks to its high spatial resolution and outstanding soft-tissue contrast 111,112.

The creation of advanced graphene-based multi-modal nanosystems for combined diagnosis and therapy has paved the way for new theranostic protocols. In this perspective, graphene is very attractive as PS agent or as a carrier for PSs for a photothermal and photodynamic alternative approach in cancer therapy, due to its excellent thermal properties and electrical conductivity 113.

In 2010, Yang* et al*. assessed the *in vivo* effects of nanographene sheets (NGS) coated by PEG and labeled with a fluorescent method 110. The fluorescence imaging showed a high NGS tumor uptake for several xenografted tumor mouse models. PEGylated NGS displayed an impressive *in vivo* behavior, including increased tumor targeting efficiency and low reticuloendothelial retention. The conjugation with PEG further improved the photothermal activity. The NGS strong optical absorbance in the NIR region allowed their use for PTT, achieving an optimal tumor ablation after their intravenous administration and tumor irradiation with low-power NIR laser.

The work of Li* et al*. represents a recent contribution for cancer diagnosis in combination with PTT, reporting the development of multifunctional NGO-based composite (UCNP@NGO), complexed with upconversion nanoparticle NaLuF4:Er3^+^, Yb3^+^, with high photothermal conversion efficiency in association with UCL imaging 84. Both *in vitro* and *in vivo* data demonstrated UCNP@NGO excellent biocompatibility and high theranostic effectiveness inhibiting tumor growth.

Also, nano-reduced graphene oxide (NrGO) was explored for imaging in association with PTT. Compared to NGS and NGO, NrGO has a higher photothermal activity due to the intrinsic physicochemical properties and uniform dispersivity. In fact, NGO reduction to NrGO causes an increased degree of π conjugation, producing an amplified NIR absorption with enhanced photothermal activity. In 2013, Sheng* et al*. developed a new protein-based method for the fabrication of NrGO, demonstrating its ability as a highly integrated theranostic agent for photoacoustic (PAI)/ultrasonic (US) dual-modality imaging and PTT. Systematic administration of NrGO displayed an optimal photoacoustic signal enhancement in the tumor area, paving its possible use for passive tumor targeting and PAI. Cancer cells, in tumor-bearing mice, were efficiently ablated due to the photothermal effect of NrGO after continuous-wave NIR laser treatment 107.

Other studies selected GO as starting material for their theranostic platform for imaging in association with PTT/PDT 73,74,84-96,99,100,102-109. For example, Wang* et al*. decorated the material with ICG as the photoresponsive imaging agent and FA as a targeting moiety 114. The resulting complex (ICG-GO-FA) exhibited a high optical absorbance in the NIR region, endowing it with excellent photothermal properties. *In vitro* data demonstrated that 1 h incubation with ICG-GO-FA (20 µg/mL), followed by 808 nm NIR laser irradiation, could induce a targeted photothermal HeLa cervical cancer cell death (8% residual cell viability) in association with PAI. In another study exploring GO potential for cancer theranostics, the PAI signals as well as the GO NIR absorbance were dramatically enhanced by the functionalization of the material with the NIR fluorescence dye CySCOOH, showing a complete PTT-induced tumor ablation *in vivo* without any sign of recurrence in the next 60 days of follow up 93.

Another GO-based theranostic platform for combined PAI and PTT was developed by Gao* et al*. 80. The authors studied a GO/gold-based probe (CPGA) with enhanced NIR absorbance and photoconversion efficiency applicable in multimodal fluorescence imaging and photoacoustic image-guided PTT of cancer. The intravenous administration of CPGA into tumor-bearing mice resulted in the observation of tumor localized high fluorescence and PA signals. Moreover, laser exposure caused tumors growth inhibition and ablation. One more successful procedure achieving total cancer elimination in mice after 808 nm laser irradiation was obtained by loading of Bi_2_Se_3_ NPs on GO in the presence of polyvinylpyrrolidone (PVP) 87. The high biocompatible nanosystem served a as an outstanding bimodal imaging (CT and PAI) in association with PTT platform for imaging-guided therapy, without any sign of tumor re-growth up to 24 days. Impressive system imaging ability in association with phototherapy was also reported in the study of Bi* et al*., where the designed GO-based nanoplatform, supported by ZnFe_2_O_4_ and UCNPs, was able to perform a quad-model imaging-guided PTT/PDD, exploiting its MRI, CT, UCL and PAT capability and obtaining a sustained tumor reduction in mice 85.

Ray* et al*. explored GO for highly selective and ultra-sensitive melanoma cancer cell detection from blood samples 99. To this end, an AGE-aptamer-conjugated magnetic hybrid GO-based assay was used as a multicolor luminescence system for tumor cells and attached with ICG for combined PTT/PDT using a 785 nm laser irradiation. In the presence of NIR light the system was highly effective, indicating good performances of the material in inhibiting tumor growth, while any reduction of cell viability was observed in the absence of the irradiation after 12 h exposure, demonstrating the biocompatibility of the nanoplatform. A novel photo-theranostic platform based on sinoporphyrin sodium (DVDMS) loaded PEGylated GO was studied in 2015 by Yan* et al*. The GO-PEG carrier improved the fluorescence of DVDMS through intramolecular charge transfer as well as the tumor accumulation of DVDMS. The NIR absorption of GO was enhanced by DVDMS, leading to improved PAI and PTT. *In vivo* results showed that systemic administration of the system could even result in total tumor eradication 94. Kalluru* et al*. reported single-photon excitation wavelength-dependent photoluminescence in the visible and short NIR region 88. When authors analyzed the formation of ^1^O_2_, it was shown that the system is suitable for the *in vivo* fluorescence imaging operated using inexpensive laser setups using low laser doses. By combining PEG and folate, nano-sized GO has been shown to effectively result in PDT and PTT *in vitro* and *in vivo* using NIR light at ultra-low doses. Yang and co-workers developed another promising gold-based GO/BaGdF5/ PEG usable as a T_1_-weighted MR and X-ray CT dual-mode contrast agent 96. GO/BaGdF5/PEG demonstrated to be an optimal photothermal agent for *in vivo* PTT cancer treatment due to its strong NIR absorbance and an improved contrast agent providing MR/CT bimodal imaging-guided therapy. Other studies further inspired the application of GO/gold hybrid nanocomposites for image-guided enhanced PTT in biomedical applications. In 2014, Nergiz* et al*. validated a novel class of multifunctional graphene/gold hybrid nanopatches consisting of GO and gold nanostars (GO-AuNS) for an improved image-guided PTT 100, whereas Jin* et al*. (2013) developed Au@PLA-(PAH/GO)n microcapsules as multifunctional theranostic agent, acting as contrast agent for both ultrasound (US) imaging and X-ray CT imaging and showing exceptional photoablation effectiveness, as suggested by the photothermal experiments 115. The use of US/CT bimodal imaging allowed to obtain an enhanced imaging contrast and specific tumor anatomic information. The contrast imaging was applied to identify the location and size of the tumor, while NIR laser-induced photothermal target therapy was carried out based on the diagnostic imaging results, avoiding damaging healthy tissues. To further improve the photothermal activity, Shi and collaborators exploited the conjugation with PEG, iron oxide and AuNPs developing GO-IONP-Au-PEG, as a powerful photothermal agent for *in vitro* cancer cell killing using molecular or magnetic targeting 116. Thanks to the subsequent *in vivo* studies, they were able to demonstrate the efficacy of this dual model imaging-guided photothermal tumor destruction method, proving an excellent tumor ablation. The study suggested that the IONP and Au substituents in the GO-IONP-Au-PEG nanocomposite structure could be further exploited for MR and X-ray dual-modal imaging.

The adoption of iron oxide could be used in order to increase in T_2_ contrast enhancement. For this reason, many studies developed graphene-iron oxide NPs with improved imaging properties. Wang* et al*. developed GO-iron oxide NPs, as a nanotheranostic agent for the diagnosis and treatment of regional lymph node (RLN) metastasis of pancreatic cancer 102. Intratumoral injection of GO-iron oxide NP resulted in its transportation to RLN *via* lymphatic vessels led to the regional lymphatic system dual-modality mapping through MRI and to efficient tumor ablation. PEGylation of the system allows achieving lower systematic toxicity, suggesting that these efficient theranostic nanoplatforms can be engineered to be safer for future clinical studies. Huang* et al*. tested iron oxide/GO-COOH nanocomposites with high photothermal conversion efficiency and enhanced contrast 91. The authors reported an effective inhibition of tumor growth due to the improved photothermal effect.

Only three studies were based on the use of GQD-based nanoplatform for combined imaging and PTT/PDT 83,97,101. For example, a theranostic probe based on SPIO and bismuth oxide (Bi_2_O_3_) with GQD coating was fabricated by the group of Mesbahi A for *in vitro* CT/MR dual-modal biomedical imaging and guided PTT 83. A high inhibitory effect on cancer cells proliferation was reported after the co-treatment with GQDs-Fe/Bi NPs and NIR irradiation, demonstrating the exceptional performance of this theranostic nanoplatform for MR imaging, high-contrast CT imaging, and CT enhancement efficiency.

The use of graphene and GRMs in PDT directed theranostics have also been reported in various studies, however to a lesser extent compared to PTT involving applications. For what concerns the adoption of graphene in the PDT protocols, in 2014, Ge* et al*. exploited the intrinsic GQDs properties, such as a broad absorption from the visible to the NIR, deep-red emission, high photo- and pH-stability and biocompatibility, for imaging purposes in association with PDT 97. GQDs exhibited a high ^1^O_2_ generation yield, enabling them to be used as *in vivo* multifunctional graphene-based nanoplatform for simultaneous imaging and extremely efficient PDT of different types of cancer, including skin melanoma and tumors located near the skin.

In cancer treatment, researchers are frequently motivated to combine different modalities to achieve an efficient cancer diagnosis and therapy. New advances have been made to establish a targeted protocol that covers the simultaneous application of imaging methods and PTT or PDT. The theranostic progress made by the previously cited studies led to the development of new combined protocols involving graphene or GRM-based nanoplatforms for simultaneous imaging, PTT, and PDT approach.

In 2013, a promising integrated probe was developed for upconversion luminescence (UCL) image-guided combinatorial PDT/PTT of cancer 102. This NGO-based multifunctional nanoplatform, UCNPs-NGO/ZnPc, could be used as UCL high contrast imaging probing of cells and whole-body for diagnosis, as well as for PDT causing the formation of cytotoxic ^1^O2 under light excitation and for PTT by converting the 808 nm laser energy into thermal energy. Another platform for combined PTT/PDT is the one developed by Cho* et al*., in which HA-conjugated Ce6 was combined with GO in order to improve biocompatibility 104. The resulting system (GO-HA-Ce6) was shown to be enzyme-activatable, which could be used for both NIR fluorescence imaging and photo-induced cancer therapy. The following year another group combined PEG-functionalized GO with the PS 2-(1-hexyloxyethyl)-2-divinyl pyropheophorbide-alpha (HPPH or Photochlor®), via supramolecular π-π stacking 103. The system showed significant improvement in photodynamic cancer cell killing efficacy due to the increased tumor delivery of HPPH. Golavelli* et al*. developed a superparamagnetic graphene-based nanoplatform; the MFGeSiNc4, an excellent T_2_-weighted MRI contrast probe 98. The graphene NIR absorption ability (600-1200 nm) and the presence of silicon phthalocyanine bis (trihexylsilyloxide) (SiNc4) facilitated the immobilization of various PSs for the achievement of both PTT and PDT effects using a single light source. *In vitro* studies have suggested that MFG-SiNc4 may thus be utilized as a potential theranostic nanocarrier for dual-modal imaging and phototherapy of cancer cells with a single light source for time and cost-effective treatments with a minimal therapy dose.

In 2015, Kim* et al*. engineered ZnPc-PEG-Au@GON NPs, in which the PS zinc phthalocyanine was loaded onto PEGylated Au@GON. The system showed promises for both combinational treatment of PTT and PDT and bioimaging. Results also suggested that ZnPc-PEG-Au@GON NPs resulted in low cytotoxicity 92. Luo* et al*. combined the PS IR-808 with NGO and studied its PDT, PTT, and imaging capabilities 90. Authors achieved high tumor accumulation by targeting organic-anion transporting polypeptides (OATPs) overexpressed in many cancer cells. Results suggested that this system (NGO-8080) can provide high-performance cancer phototherapy with minimal side effects through the synergistic PDT/PTT treatment and cancer-targeted accumulation. In 2018, Gulzar* et al*. covalently implanted upconversion NPs (UCNPs) with PEGylated NGO and loaded the system with the PS Ce6 86. The authors reported a significantly enhanced and synchronized therapeutic effect paralleled to the individual PTT or PDT. Therefore, this study showed that this multifunctional nanohybrid could be used as a potential theranostic probe for upconversion luminescence (UCL) imaging-guided combinatorial PDT/PTT.

#### 3.1.3. Imaging, drug/gene delivery, and photodynamic/photothermal therapy

The real potential of the graphene-based nanoplatforms lies in the possibility of combining multiple strategies to fight cancer in a single platform. As reported by Gazzi* et al*. 117, the opportunity to associate imaging diagnostic methods, drug delivery, PTT with PDT opens the way to new approaches and enhances the efficacy of the single modalities.

A typical example of this enhancement is well represented by the work of Feng L* et al*., where the authors fabricated a pH-responsive nanocarrier. NGO was coated with two polymers, PEG and poly (allylamine hydrochloride) (PAH), modifying the latter with 2,3-dimethyl maleic anhydride (DA) in order to acquire pH-dependent charge reversibility. The nanocarrier was then loaded with DOX, to form the NGO-PEG-DA/DOX complex which showed responses to pH change, enhanced cellular uptake, augmented DOX release in the tumor microenvironment and inside cellular lysosomes. The slow efflux of DOX from NGO-PEG-DA/DOX offers an enhanced killing of drug-resistant cancer cells compared with free DOX. Moreover, NGO-PEG-DA/DOX has an excellent photothermal conversion ability; therefore, a synergistic therapeutic effect was realized combining chemo- and PTT 118. Similarly, a pH-responsive nanoplatform for controlled drug release was developed by Battogtokh* et al*. where the author used a GO-based nanocarrier for pH-dependent release of the PS that was also used as a fluorescent imaging agent 119.

Five studies have used rGO as a starting material for combined therapy and imaging 120-124. For example, Shervedani* et al*. developed, rGO-PDA-BSA-DTPA-Mn(II)/MTX, a high biocompatible system for breast cancer-selective PTT 120. In this system, GO was partially reduced and functionalized with dopamine to obtain a reduced graphene oxide/polydopamine system (rGO-PDA). As a highly promising carrier in drug delivery, bovine serum albumin protein (BSA) was grafted onto the obtained system (rGO-PDA-BSA). Finally, the researchers adopted the decoration with diethylenetriaminepentaacetic acid (DTPA)-Mn(II) to achieve diagnosis and methotrexate for anticancer therapy.

A new functionalization strategy was proposed, where the photoresponsive imaging agent ICG has been loaded onto hyaluronic acid-anchored (HA) rGO nanosheets in order to enhance the photothermal properties of the system. Compared to rGO, HArGO resulted in enhanced tumor cell targeting *in vitro* and *in vivo* 123. Moreover, the ICG/HArGO targeted delivery has been shown to guide authors to identify the most relevant area for NIR irradiation to achieve PTT. Exploiting another functionalization strategy, a Gd-functionalized GO nanoplatfom for MRI guided photothermal-chemotherapy was developed 125. They have shown neglectable toxicity both *in vitro* and *in vivo* studies, GO@Gd-PEG-FA was able to kill cancer cells selectively demonstrating to be an excellent MRI guided photothermal-chemotherapeutic system with drug delivery and tumor-targeting properties. In 2012 a new system characterized by biocompatibility, high DOX loading capacity, NIR photothermal heating, facile magnetic separation, and large T_2_ relaxation rates (r2) was successfully developed 72. A single system for image-guided glioma therapy with integration of MRI, dual-modal recognition (magnetic and receptor-mediated active targeting), and chemo-photothermal therapy was developed by Chen* et al*., based ongraphene-gold nanohybrids, GO-Au@PANI, with excellent NIR photothermal transduction efficiency and ultrahigh drug-loading capacity 126. By using this ultrasensitive nanoprobe, cancer cells were analyzed through SERS-fluorescence dual-mode imaging, confirming optimal DOX-loading efficiency and delivery accompanied by an increased sensibility of NIR/pH-responsive release. The GO-Au NPs decorated with PANI, a new NIR PTT agent characterized by a strong NIR absorption, allowed the *in vitro* and *in vivo* chemo-photothermal ablation of breast cancer cells. Chang* et al*. produced a PEGylated GO/MnWO_4_ nanocomposite (GO/MnWO_4_/PEG) for dual imaging (MRI and PAI) and therapy (chemotherapy and PTT). *In vivo* data demonstrated that the nanosystem was an excellent bimodal contrast agent to aid the delivery and pH- and NIR-light dependent release of DOX in breast cancer 127. Another study using DOX as antitumoral drug for combined drug delivery and PTT in an MRI-capable nanomediator (rGO-Fe_2_O_3_@Au NPs) was reported by Chen* et al*. The superparamagnetic nanoplatform showed a high photothermal conversion efficiency (under 808 NIR laser irradiation) and an excellent drug loading ability in cervical cancer *in vitro* (HeLa cells) 124. The delivery of DOX in association with PTT was also evaluated by Khatun and co-workers 128. Their graphene-based nanosystem conjugated with a hyaluronic acid nanogel for photothermal imaging was able to induce an effective killing of lung cancer cells (A549), while showing only minor toxicity in the non-tumor MDCK cells. Jin and co-workers combined Au NPs into poly(lactic acid) microcapsules for combined drug delivery and PTT in a GO-based nanosystem to enhance ultrasound imaging and X-ray CT imaging 115. Thakur* et al*. developed a graphene-based nanoplatform for simultaneous imaging and combined drug delivery, PTT, and PDT. The nanosystem was able to perform as a NIR imaging agent and to induce the ablation on the tumor *in vivo* 60. In 2014, Bian and collaborators fabricated another graphene-isolated-Au-nanocrystal (GIAN) for multimodal cellular imaging by means of Raman scattering and NIR two-photons luminescence 129. Besides having an exploitable DOX loading capability, graphene-isolated-Au-nanocrystal (GIAN) showed NIR absorption that allowed using them also for PTT. Using NIR heating, they obtained a controlled release of DOX, drastically reducing the possibility of side effects in chemotherapy. In the same year, a GO-Au nanohybrid was fabricated by chemical deposition of Ag NPs onto GO through a hydrothermal reaction 106. DOX was loaded to obtain an anticancer activity. Functionalization of GO@Ag-DOX nanohybrid with DSPE-PEG2000-NGR, resulted in GO@Ag-DOX-NGR, a particular theranostic nanoplatform with powerful tumor-targeting capability, excellent stability in physiological solutions and a much higher antitumor efficacy without toxic responses owing to the higher DOX uptake at the tumor site. Moreover, GO@Ag-DOX-NGR not only served as a diagnostic X-ray contrast probe, but also as a potential agent for chemo and photothermal therapy. GO@Ag-DOX-NGR was demonstrated to have an ideal tumor-targeting capability with NIR laser-controlled drug release and X-ray imaging ability, ensuring a significant chemo-photothermal therapeutic efficacy.

Three studies also explored GQDs for combined multiple therapies and imaging 121,130,131. In the first study, a porous silica NPs-based nanosystem, encapsulated with the PS hypocrellin A and GQDs, allowed combining multicolor imaging and cervical cancer treatment 131. In more recent work, porphyrin derivatives were conjugated to an aptamer-functionalized GQDs for miRNA delivery and simultaneous PTT /PDT, allowing CLSM of lung and breast cancer cells 130.

Finally, concerning the simultaneous use of imaging, PTT, and drug delivery strategies, Chen and co-workers used a valuable approach 121. They fabricated biocompatible and photoluminescent GQDs which, in conjunction with superparamagnetic iron oxide nanoparticles, was exploited for both fluorescent and MR imaging without the use of any other fluorescent dyes. Thanks to the external magnetic stimulation, the drug has been continuously and slowly released by the GQDs *in vitro*. The NIR irradiation of HeLa cells and their consequent photothermal ablation confirmed their potential use for cancer PTT. Despite the few numbers of publications, we can highlight three different studies reporting methods based on PDT combined with imaging and drug delivery 132-134. In 2015, Yan* et al*. validated the effects of GO-PEG DVDMS for drug delivery, *in vivo* imaging, and PDT 132. Sinoporphyrin sodium (DVDMS), a novel photo-theranostic agent, was successfully loaded to PEGylated GO via intramolecular charge transfer, enhancing its fluorescent imaging and improving its tumor accumulation. In the same year, an MRI GO/MnFe_2_O_4_ nanohybrid was fabricated with very low cytotoxicity and negligible *in vivo* toxicity 133. MRI experiments demonstrated that the large magnetic spin magnitude of manganese ferrite (MnFe_2_O_4_) NPs make them suitable as reliable T_2_ contrast enhancement agents. The GO/MnFe_2_O_4_ optical absorbance in the NIR region and the optimal photothermal stability resulted in the highly effective photothermal ablation of HeLa cancer cell lines. In this study, nanohybrids were further tested for chemotherapeutic purposes by combining with DOX-induced chemotherapy. An enhancement in cancer cell killing activity was achieved after GO/MnFe2O4/DOX irradiation with NIR light, when compared to free DOX. Finally, Wu* et al*. fabricated a graphene-Au nanostar hybridized system (denoted as GO/AuNS-PEG) in order to achieve single wavelength laser-induced synergistic PDT and PTT and active cancer photothermal/fluorescence multimode imaging 134. The system was shown to be biocompatible *in vitro* and *in vivo*. Under the NIR laser irradiation, authors achieved high dual-enhanced photothermal efficiency, even for tumors found at deep locations. When the system was combined with the PS Ce6, both *in vitro* and *in vivo* data confirmed that efficient photoablation of tumors was achieved through the synergistic PDT and PTT effect under the activation of a single wavelength laser.

#### 3.1.4. Imaging and other new therapies

Beyond drug delivery, PTT, and PDT, other non-conventional therapies, like sonodynamic therapy (SDT) and immunotherapy, can also be enhanced by the use of graphene, GRMs, and graphene hybrid nanosystems. The following sections give a short description of the recent advances of these materials used for these applications.

***i) Sonodynamic therapy (SDT).*** Ultrasound-based therapies are opening new prospects in the oncological field; amongst them, SDT emerged very recently as a novel cancer treatment approach. Being more effective than PDT due to the higher tissue penetration depth 135,136, SDT consists in the use of ultraviolet light as an external stimulus in order to activate a sonosensitizer, a non-toxic and selective chemical agent that once activated is able to induce ROS synthesis and thermal effects.

Despite nanoparticle-assisted ultrasound therapy being still under development in the clinical field 137, different cell death pathways involved in the SDT process have been identified. To this end, Dai* et al*. have synthesised a novel nanoplatform for MRI-guided SDT and PTT by the functionalization of rGO with TiO_2_ NPs 135,138. Graphene's high electrical conductivity enableds the separation of the sono-generated electron-hole pairs, leading to an increased in vitro ROS production.

Recently, Huang* et al*. translated SDT into a theranostic context, by developing a graphene-based nanoplatform for PAI-guided SDT in breast cancer cells and tissues, paving a new path toward targeted medicine 139.

***ii) Immunotherapy.*** Immunotherapy is a new therapeutic approach that allows precise cancer treatment enhancing or restoring the patients' own immune system's ability to fight cancer 140. The introduction of 2DMs in these fields is aimed at improving the patients' outcome expanding the combination of possible different therapeutic and imaging modalities in a single nanosystem.

For example, in 2014, GO was explored both for PTT and the delivery of CpG oligodeoxynucleotide 141. The latter can be recognized by Toll-like receptor 9 leading to the secretion of proinflammatory cytokines, resulting in the activation of innate and adaptive immune responses 142. The intracellular trafficking of nanocarriers was improved thanks to the local heating allowed by the optimal nanosystem NIR optical absorbance, leading to an efficient tumor reduction *in vivo*.

Recently, Wang* et al*. developed a PEGylated-rGO nanoplatform hybridized with Fe_3_O_4_ nanoparticles for breast cancer dual treatment based on PTT and immunotherapy 143. Thanks to the incorporation of Fe_3_O_4_ nanoparticles, the resulting nanosystem induced the reduction of tumor-associated macrophages and the activation of dendritic cells in tumor draining lymph nodes in tumor-bearing mouse model. Moreover, the authors envisage the application of their smart nanocomposite for MRI-guided therapy. However, only recently the first attempt to apply GRMs for immunotherapy in the field of cancer theranostics was made by Wu and co-workers. In this study, polydopamine stabilized GQD PSs were integrated with immunostimulatory polycationic polymer/CpG oligodeoxynucleotide nanoparticles and Gd^3+^/Cy3 imaging probes, enabling dual imaging (i.e., MRI and fluorescence imaging)-guided photoimmunotherapy 144. The resulting theranostic nanostystem allowed the effective eradication of tumor in a murine mammary cancer model, because of the combination of enhanced PDT and PTT exerted by GQDs, together with the simultaneous activation of endosomal Toll-like receptor 9 mediated by the polycationic polymer/CpG oligodeoxynucleotide. This immuno-stimulation resulted in the secretion of proinflammatory cytokines and dendritic cell maturation, ultimately triggering the activation and infiltration of T cells.

### 3.2. 2D materials beyond graphene for cancer theranostics

After the rise of graphene, the whole family of 2DMs has started to be investigated for several applications, including the fight of cancer. GDY and other emerging 2DMs, such as WS_2_, MoS_2_, hBN, BP, and MXene, are under consideration for their use as cancer theranostic tools 145. All the reports present in the literature for these materials as theranostics are discussed and reported in **Table 4**. The following sections are dedicated to each type of material.

#### 3.2.1. The 2D family of transition metal dichalcogenides

TMDs have the empirical formula MX_2_; where M is a Group 6 transition metal (usually Mo or W) and X is a group 16 calcogen (S, Se or Te). In bulk form, TMDs are layered compounds which can be exfoliated down to few and single layers. In particular, in the single-layer form, many TMDs show strong light-matter interaction, i.e., strong absorption of light in the visible-near IR range and emission of light. The TMDs investigated so far in theranostics are tungstenum disulfide and molybdenum disulfide.

***i) Tungsten disulfide.***Tungsten disulfide has received considerable attention in recent years, thanks to the fascinating physicochemical properties that the whole family of TMDs have in common. WS_2_ is a well-known solid lubricant for its tribological near-zero friction or superlubricity and possesses a lamellar structure similar to that of MoS_2_, allowing it to be exfoliated into nanosheets 146, which can be loaded with drugs as drug delivery carriers, exactly as done for graphene.

The capability of WS_2_ to serve as a cancer nanoteranostic tool mainly derives from its strong absorbance in the NIR region and X-ray attenuation ability, making it a suitable photothermal and contrast agent for PAT and CT bioimaging-guided diagnosis and therapy. Exploiting its strong absorbance in the NIR region, Cheng* et al*. reported for the first time the opportunity of using WS_2_ nanosheets as novel agents for PTT in association with multimodal bio-imaging, obtaining a highly effective photothermal ablation of tumor in a mouse model 147. Their PEGylated WS_2_ nanosheets enabled an excellent NIR light-triggered tumor ablation after both intratumorally (low dose, 2 mg/kg) and intravenous injection (high dose, 20 mg/kg). Moreover, PEG-WS_2_ nanosheets demonstrated to serve as a bimodal contrast agent for CT and PAT imaging, due to their strong X-ray attenuation ability and the high NIR optical absorbance, respectively.

Another example of WS_2_ nanosheets-based theranostic application is represented by the work of Yong* et al*. 148. In this case, the developed WS_2_ nanosheets were employed not only as NIR absorbing agents for PTT, but also as PS carriers for PDT. Moreover, the PSs release behavior from WS_2_ nanosheets could be controlled by NIR irradiation manipulating ^1^O_2_ generation of the PSs-WS_2_ complex.

WS_2_ was used for theranostic purposes as reported by the work of Yang* et al*., where IONPs were adsorbed on PEGylated WS_2_ nanoflakes, which were subsequently coated with silica and manganese dioxide 149. The resulting nanoplatform (WS_2_-IO/S@MO-PEG) appeared to be highly sensitive to pH, enabling tumor pH-responsive MR imaging using IONPs and MnO_2_ as pH-inert T_2_ contrast probe and pH-sensitive T_1_ contrast probe, respectively. WS_2_-IO/S@MO-PEG allowed the synergistic combination of NIR light and X-ray absorbance of WS_2_ for PTT and cancer radiotherapy, resulting in remarkable tumor destruction.

Furthermore, with regards to cancer radiotherapy, Chao* et al*. have recently tested a WS_2_-based theranostic nanosystem functionalized with PEG and labeled with ^188^Re, a widely-used radioisotope for radioisotope therapy (RIT) 150. The ^188^Re labeling of WS_2_-PEG nanoflakes enabled not only the enhancement of RIT, but also allowed the *in vivo* tracking of the nanoflakes after their administration into animals.

***ii) Molybdenum disulfide.***Molybdenum disulfide is another relevant member of the 2D family of TMDCs, with properties very similar to WS_2_. Thus, it has been investigated as a theranostic cancer nanoplatform with multiple highly integrated functionalities 151. In particular, it has been explored as a photothermal agent for cancer PTT and as a nanocarrier for drug delivery-based therapeutic approaches. The principal limit for MoS_2_ application in biomedicine appears to be mainly related to its low stability in the biological milieu 152. Therefore, in the majority of the studies, different water-soluble and biocompatible molecules were applied to functionalize MoS_2_ to overcome this issue. In 2014, Liu* et al*. have reported the first demonstration for the use of MoS_2_ in cancer theranostics, modifying the material to obtain a high biocompatible PEGylated MoS_2_ loaded with the photodynamic agent Ce6 (MoS_2_-PEG/Ce6). The nanocarrier was able to enhance the intracellular delivery of Ce6, dramatically increasing the PDT efficacy *in vitro* in breast cancer cells (471 cell line) exposed to 660 nm light irradiation. Moreover, it further increased after induction of a moderate hyperthermia (808 nm laser irradiation) to promote the cellular uptake. Moreover, both *in vitro* and *in vivo* data demonstrated the ability of MoS_2_-PEG/Ce6 to promote tumor ablation exploiting a combined PTT and PDT synergistic affect triggered by laser with wavelengths of 808 nm and 660 nm, respectively. Also, the PEGylated MoS_2_ nanosheets served as a contrast agent for photoacoustic tomography (PAT) allowing the *in vivo* tracking of the nanosystem, and the effective photothermal heating was confirmed by IR thermal imaging 153.

In their subsequent study, in addition to PAT, the imaging was also performed in association to MR and PET in mice, thanks to the superparamagnetic properties of PEGylated MoS_2_ decorated with iron oxide (MoS_2_-IO-PEG) and to the adsorption of the positron-emitting radioisotope ^64^Cu, respectively. The triple-modal imaging-guided tumor PTT by the 808 nm laser resulted in the complete elimination of 471 breast cancer tumors *in vivo* 154. In the same year (2015), Wang* et al*. produced a MoS_2_/Bi_2_S_3_-PEG (MBP) composite, with enhanced colloidal stability and biocompatibility, for PTT combined with photoacoustic and CT imaging, demonstrating its therapeutic efficacy *in vitro* and tumor-bearing 4T1 mice 155. In the meantime, Yu at al. created MoS_2_/Fe_3_O_4_-PEG (MSIOs) composite consisting of NIR-absorbing MoS_2_ flakes for PTT decorated with Fe_3_O_4_ nanoparticles as target moieties spatially/timely guided by an external magnetic field to cancer tissue 156. Thanks to their superparamagnetic property and high NIR absorption, MSIOs nanosystem for magnetic targeted PTT was applied for MR and PAT imaging *in vitro* for cervical (HeLa cells) and liver cancer (HepG2 cells). Soon after, in 2016, Li* et al*. synthesized biocompatible soybean phospholipid-encapsulated MoS_2_ (SP-MoS_2_) nanosheets for *in vitro* and *in vivo* highly efficient breast cancer PTT using a laser with wavelengths of 808 nm and IR thermal imaging 157. The encapsulation of the nanosystem with soybean phospholipids was used as an alternative to PEG functionalization to enhance its colloidal stability, allowing improving the therapeutic performance. The following study aimed at exploiting MoS_2_ proprieties for cancer theranostics was based on a different kind of material synthesis, where polyvinylpyrrolidone (PVP) was used not only to enhance the colloidal stability but also to direct the growth of MoS_2_ nanosheets to produce an ultra-small MoS_2_-PVP composite for *in vitro* and *in vivo* combined PTT and PAI 158.

In 2017, Xu* et al*. integrated MoS_2_ nanosheets with IR-808 sensitized upconversion nanoparticle (UCNP) loaded with Ce6 for synergistic PTT and PDT *in vitro* and *in vivo*, simultaneously achieving trimodal UCL, CT, and MR imaging under a single 808 nm wavelength laser excitation 159. Similarly, Liu* et al*. designed a trimodal imaging (IR, PA, and MRI) Mo@Fe-ICG/Pt multifunctional nanocomposite, consisting of polyethyleneimine (PEI) functionalized MoS_2_ nanosheets ingeniously decorated with Fe_3_O_4_ nanoparticles and loaded with ICG molecules and platinum (IV) as PSs and prodrugs, respectively 160. The resulting nanosystem showed a remarkable tumor cell killing ability both *in vitro* and *in vivo* by taking advantages of the synergistic PTT, PDT, and chemotherapy triggered by a single 808 nm NIR laser. In the same year, Cheng at al. conjugated bovine serum albumin-gadolinium (BSA-Gd) complexes with MoS_2_ nanoflakes (MoS_2_-Gd-BSA) for PTT and bimodal MR and PA imaging 161. The MoS_2_-Gd-BSA biocompatible nanosystems presented excellent tumor cell inhibition effectiveness *in vitro* and could totally ablate cancer *in vivo* using 808 nm laser irradiation, without any sign of recurrence in the next two weeks of follow up. Maji at al. synthesized a hybrid gold nanobipyramid nanostructure coated with MoS_2_ (AuNBPs@MoS2) for enhanced photothermal conversion and ROS production-based cancer treatment and simultaneous two-photon luminescence imaging in HeLa cells 162. Most recent works explored a therapeutic approach based on drug delivery to counteract drug-resistant cancer. In 2018, Dong *et al*. used a biodegradable hyaluronic acid (HA) and PEI-decorated MoS_2_ nanocarrier (MoS_2_-PEI-HA) loaded with DOX 163. HA was used as a targeting moiety against CD44-overexpressing breast cancer cells (MCF-7-ADR) and, thanks to its localized biodegradation by hyaluronidase concentrated at the tumor site, it also served for controlled and faster DOX release. Moreover, the composite was labeled with ^64^Cu for PET imaging *in vitro* and *in vivo*. Another study also exploited drug delivery through the loading of chitosan and metformin on Mn-doped Fe_3_O_4_@MoS_2_ composites for combined PTT and MR imaging 164. *In vitro* data demonstrated that the load with metformin led to hepatic cancer ablation (Hep3B cells) not affecting healthy cell viability (LO2 cells), while chitosan, besides enhancing the dispersibility and biocompatibility of the nanoplatform, and was able to improve the PTT efficiency. Finally, a similar approach was employed in a very recent publication concerning the use of MoS_2_ for cancer theranostics 165. In this work, hyaluronic acid (HA)-functionalized MoS_2_ nanosystems were applied to deliver Gd and the anticancer drug gefitinib (Gef) for combined chemo-PPT and MR. This smart nanoplatform induced the inhibition of lung cancer *in vitro* and *in vivo*.

#### 3.2.2. Black phosphorus

Phosphorous is one of the most abundant elements on Earth and can exist in various allotropes, namely white, red, black, and violet phosphorus. Bulk black phosphorus (BP), the thermodynamically most stable allotrope of phosphorus 166, is one of the emerging monoelemental materials, which have shown outstanding potential in biomedical applications 167,168. It is a semiconductor 2DM with a tunable bandgap dependent on its thickness. In the single-layer form, each phosphorus atom is covalently bonded with the P atom on both sides of the same plane, and the other P atom is in the neighboring plane through the 3p orbitals. BP actively interacts with incident light, owing to its tunable bandgap ranging from UV to NIR, depending on the thickness. Thus, this opens the door for its possible applications in various fields, including bio-photonics, photocatalysis, and bioimaging 169. In one of the earliest theranostic studies with BP, Sun* et al*. used PEGylated BP nanopartcles in order to have better stability (single and few-layers BP are unstable in the air), biocompatibility and long-blood circulation. Following PAI *in vivo*, BP nanoparticles were shown to accumulate in through the enhanced permeability retention effect. NIR light irradiation of these tumors after material injection resulted in photothermal ablation of tumor 170. Later, other studies used PEGylated BP nanoparticles to improve their anti-cancer activity in theranostics by combining with AuNPs 171 or chemotherapeutic drug DOX 172. Among the attempts to improve imaging properties, BP has been conjugated to other nanoplatforms for MR or PAI 173-177.

#### 3.2.3. MXenes (carbides and nitrides)

**Figure 2:**
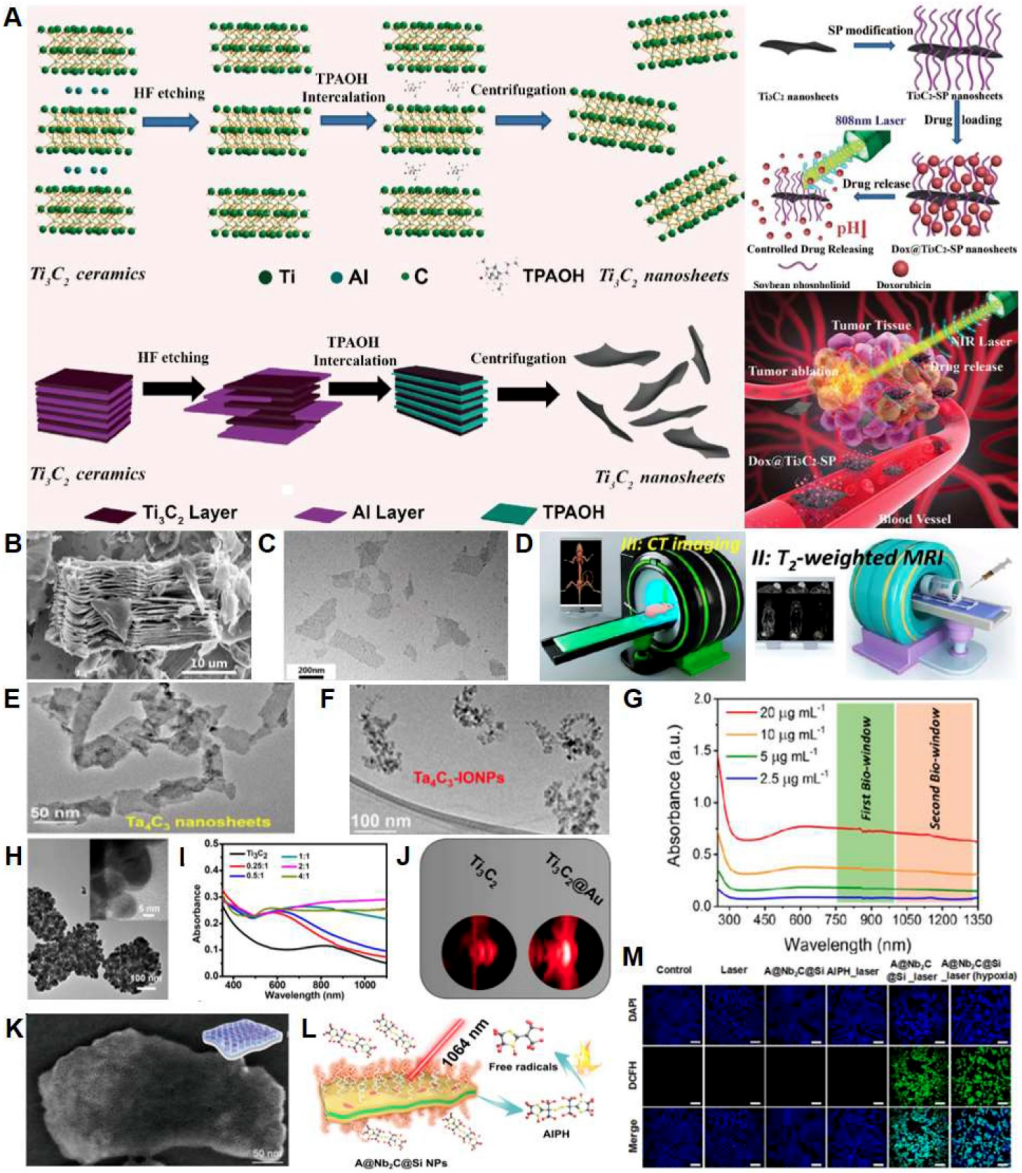
Illustration of MXene properties. (A) Schematic representation of Ti_3_C_2_ MXene NSs synthesis, functionalization with SP and DOX for colloidal stability and multistimuli-triggered chemotherapy, and illustration of a synergistic platform for cancer therapy based on Ti_3_C_2_ MXene NSs. (B) Scanning electron microscopy (SEM) of Ti_3_C_2_ after HF etching of the A phase and (C) transmission electron microscopy (TEM) of Ti_3_C_2_ NSs after exfoliation. Adapted with permission from 182, copyright 2018 Advanced Healthcare Materials. (D) Dual-mode CT/MR imaging contrast-enhanced by Ta_4_C_3_. (E, F) TEM of Ta_4_C_3_ (E) before and (F) functionalization with IONPs. Adapted with permission from 188, copyright 2018 ACS Nano. (G) UV-VIS-NIR absorption spectra of Nb_2_C MXene NSs at different concentrations. Adapted with permission from 189, copyright 2018 Journal of the American Chemical Society. (H) TEM of Ti_3_C_2_@Au nanocomposites. (I) UV-VIS-NIR absorption spectra of Ti_3_C_2_@Au-PEG at varying Au/Ti ratios. (J) PAI enhancement with Ti_3_C_2_@Au compared to Ti_3_C_2_ at the same Ti_3_C_2_ concentration (0.5 mg/mL). Adapted with permission from 190, 2018 *ACS Nano*. (K) High-resolution SEM of CTAC@ Nb_2_C-MSN NSs. Reproduced with permission from 192, copyright 2018 Theranostics. (L) Schematic illustration of free-radical generation with AIPH@Nb_2_C@mSiO_2_ nanocomposites under NIR irradiation at 1064 nm. (M) Photo-thermodynamic free-radical generation with AIPH@Nb_2_C@mSiO_2_ in 4T1 breast cancer cells detected by dichlorodihydrofluorescein diacetate (DCFH-DA) fluorescence under normoxic and hypoxic conditions. Adapted with permission 193 from 2019 *ACS Nano*.

MXenes are among the most recently discovered 2DMs 178. The term MXenes comprehensively defines early-transition-metal carbides, carbonitrides, and nitrides characterized by structural formula Mn+1Xn, where M is an early transition metal, X is carbon, nitrogen, or both, and n = 1-3. MXenes are synthesized by selective etching the A-group element from the precursor ternary-layered carbides of MAX phases, where A is a group 12-16 elements of the periodic table (**Figure 2A-C**). MXenes contain abundant surface-terminating functional groups, such as hydroxyl (-OH), oxygen (-O), or fluorine (-F), which endow them with a hydrophilic nature and allow flexible surface modification, functionalization, and scalable processability. MXenes share a number of the advantageous properties of 2DMs for application in biomedicine stemming from their topology, including extreme thinness, high surface area, high surface-to-volume ratio, and mechanical toughness. Furthermore, they show remarkably high volumetric capacitance (1,500 F/cm^3^) 179 and metallic conductivity (~10,000 S/cm) 180, and rich surface chemistry for enzyme or drug functionalization. The field of cancer theranostics, however, is undoubtedly spearheading the field of biomedical applications of MXenes as they are increasingly attracting attention as PTAs for synergistic chemo/photothermal (PTT)/PDT and imaging, due to the early recognition of outstanding internal photothermal conversion efficiency and light absorption capability 181.

Han* et al*. demonstrated a platform for PTT, chemotherapy, and imaging-based on Ti_3_C_2_ nanosheets (NSs, **Figure 2C**) functionalized with soybean phospholipids (SP), which significantly improved the colloidal stability of Ti_3_C_2_ NSs in a physiological environment without affecting their photothermal properties (**Figure 2A**) 182. UV-VIS-NIR absorption spectroscopy on aqueous Ti_3_C_2_ solutions showed a characteristic spectrum with peak absorption at ~800 nm, right within the first NIR absorption window. The abundant OH- surface terminations on in Ti_3_C_2_-SP MXenes were used to adsorb electrostatically cationic molecules such as DOX (**Figure 2A**) and achieve a drug-loading capability of up to 211%. Once the Ti_3_C_2_-SP-DOX NSs penetrated inside 4T1 breast cancer cells, DOX release was triggered endogenously by the acidic tumor microenvironment through the interference of H+ ions with the electrostatic interactions between DOX and Ti_3_C_2_-SP, or exogenously via NIR irradiation (**Figure 2A**). Compared to single therapeutic modalities, Ti_3_C_2_-SP-DOX synergistic chemotherapy and PTT led to enhanced inhibition of 4T1 cancer cell viability *in vitro* as well as to improved therapeutic and recurrence outcomes *in vivo* in 4T1 breast cancer-bearing mice. Furthermore, Ti_3_C_2_-SP showed significant contrast-enhancement for intratumor PAI up to 24h post-injection. In a similar system, Liu* et al*. first demonstrated the feasibility of Ti_3_C_2_ NSs as PSs for PDT in a synergistic platform for PTT/PDT/chemotherapy. The Ti_3_C_2_ NSs-mediated generation of reactive oxygen species (ROS) was investigated with 1,3-diphenyl-sobenzofuran (DPBF) as the singlet oxygen (^1^O_2_) detector. NIR irradiation of Ti_3_C_2_ NSs at 808 nm for 10 min, led to a ~80% decrease in DPBF absorbance at 420 nm, thus revealing the generation of ^1^O_2_. ROS production, although less pronounced, was also observed when Ti_3_C_2_-DOX NSs were exposed to the same irradiation protocol 183. Proposed mechanisms for ^1^O_2_ generation in Ti_3_C_2_ attributes it to the energy transfer of photoexcited electrons from Ti_3_C_2_ to triplet oxygen (ground state oxygen, ^1^O_2_), similar to the photodynamic behavior of graphene quantum dots 184 and black phosphorous 185. Synergistic PTT/PDT/chemotherapy with Ti_3_C_2_-DOX led to significant improvements in therapeutic efficacy and recurrence outcomes against human colon carcinoma (HCT-116) *in vivo* in tumor-bearing mice. The abundant surface terminations in the Ti_3_C_2_ NSs also enabled functionalization for specific tumor targeting, such as hyaluronic acid coatings which increased colloidal stability and enabled active targeting of the surface protein CD44+ overexpressed in cancer cells.

To expand the specific tumor-imaging capabilities and realize a theranostic platform integrating multimodal imaging, such as PA, magnetic resonance (MR) and X-ray computerized tomography (CT) imaging (**Figure 2D**) it is possible to substitute Ti in the M-phase with Ta, a transition metal with higher atomic number (Z=73) and X-ray attenuation coefficient (4.3 cm^2^ g^-1^ at 100 keV) 186. Ta_4_C_3_ MXene displays concentration-dependent broadband light absorption in the UV-VIS-NIR 187,188 and high photothermal conversion efficiency (>30%). Ta_4_C_3_-SP MXene functionalized with MnO_x_ 187 or superparamagnetic iron-oxide nanoparticles (IONP, **Figure 2E-F)** 188 realized a multimodal imaging and therapeutic platform where Ta enabled enhanced-contrast *in situ* CT imaging, MnO_x_ or IONP were the contrast agents for MRI and Ta_4_C_3_ was the high-efficiency PTA for PAI and PTT.

The possibility of working with PTA with high conversion efficiency also in the NIR II biowindow (1000-1300 nm) offers several advantages, including higher tissue penetration depth and maximum permissible exposure limit (1 W cm^-2^) compared to 808 nm (0.33 W cm^-2^, ANSI Z136.1-2007, American National Standard for Safe Use of Lasers). Compared to Ti_3_C_2_ and Ta_4_C_3_, Nb_2_C MXene shows a strong and almost constant optical absorption in the NIR I and NIR II biowindows 189 (**Figure 2G**). Taking advantage of this favorable broadband absorption, Lin* et al*. demonstrated a theranostic platform for PTT and PAI in 4T1 breast cancer cells based on Nb_2_C NSs stabilized with biocompatible polyvinylpyrrolidone (PVP) with photothermal conversion efficiencies of 36.5% at 808 nm and 46.65% at 1064 nm, respectively 189. In the attempt to extend the optical absorbance range of Ti_3_C_2_, Tang* et al.* proposed a Ti_3_C_2_-Au nanocomposite synthesized by seed-growing Au on the surface of the Ti_3_C_2_ NSs (**Figure 2H**), followed by PEGylation to achieve colloidal stability. The Au decorations significantly enhanced the photothermal conversion efficiency compared to Ti_3_C_2_ NSs of both at 808 nm (34.3% Vs. 30.7%) and 1064 nm (39.6 Vs. 28.3%, **Figure 2I**). Furthermore, the strong X-ray absorption capability enabled simultaneous CT and PAI up to 24-h post-injection (**Figure 2J**), as well as a synergistic PTT/radiotherapy platform. Interestingly, synergistic PTT/ radiotherapy appeared to most effectively inhibit breast cancer growth *in vivo* compared to PTT or radiotherapy alone 190.

Possible challenges when engineering 2DMs as drug-delivery carriers are the lack of a confined space for high loading of drugs, as well as achieving optimal drug release profiles. To enhance drug loading/release capabilities and tumor targeting Li* et al.* surface-engineered Ti_3_C_2_ MXene via sol-gel chemistry to create a mesoporous silica shell directly on the NSs (Ti_3_C_2_@mMSNs) using cetanecyltrimethylammonium chloride (CTAC) as the mesopore-directing agent and tetraethylorthosilicate (TEOS) as the silica precursor in an alkaline environment. The resulting Ti_3_C_2_@mMSNs showed a uniform structure with high pore volume (0.96 cm^3^ g^-1^) with an average size of 3.1 nm. The NSs were also functionalized with arginine-glycine-aspartic acid (RGD) for specific tumor targeting and DOX for chemotherapy. Drug delivery through the Ti_3_C_2_@mMSNs was triggered via combined pH modulation, with an enhanced release in an acidic environment, and local hyperthermia through NIR irradiation 191. As the positively-charged surfactant, CTAC shows chemotherapeutic effects, Han* et al*. 192 proposed a similar surface-engineering scheme based on 2D Nb_2_C MXenes coated with mesoporous silica shells (CTAC@ Nb_2_C-MSN) and functionalized with RGD (**Figure 2K**).

Due to the mesoporous silica reservoir and the broadband absorption of Nb_2_C NSs, the CTAC@ Nb_2_C-MSN display a drug loading capacity of 32.7% and photo-triggered release upon irradiation at 1064 nm, although the photothermal conversion efficiency appears to be lower than unmodified Nb_2_C-PVP NSs 189. Feasibility of CTAC@ Nb_2_C-MSN as PTA in a synergistic PTT-chemotherapy and PAI theranostic platform was demonstrated against U87 glioblastoma cell lines *in vitro* and *in vivo* on glioblastoma-bearing mice.

Effective generation of ROS is critical for PDT. ROS generation is controlled by local oxygen tensions, which affects PDT efficiency in the hypoxic tumor microenvironment. To circumvent this issue, Xiang* et al.* developed an alternative “thermodynamic” cancer treatment strategy based on the thermally-activated free-radical generator 2, 2′-Azobis[2-(2-imidazolin-2-yl)propane] dihydrochloride (AIPH) loaded into the mesopores on a silica-coated Nb_2_C MXene (AIPH@Nb_2_C@mSiO_2_, **Figure 2L**). AIPH is a hydrophilic and thermo-labile molecule that can generate ROS independent of oxygen concentration. Therapeutic efficacy of the synergistic PTT and free-radical generation via hyperthermia under NIR-II irradiation was demonstrated *in vitro* in both normoxic and hypoxic conditions (**Figure 2M**) and *in vivo*, resulting in complete tumor eradication in breast cancer-bearing mice. AIPH@Nb_2_C@mSiO_2_ was also demonstrated to be an effective multimodal imaging agent for real-time monitoring of the cancer therapeutic process via PAI, fluorescence and photothermal imaging 193.

The results obtained thus far on MXene-based theranostic platforms are encouraging. However, the field is still in its infancy and there is extensive work to be done in order to fully realize the potential of MXenes as nanoagents for multimodal cancer therapy and imaging. Future work is required to elucidate the fundamental principles controlling the photothermal conversion and ROS generation activity of MXenes, as well as to fully expand and explore the capabilities of the different species available in the library of MXenes. Most importantly, the long-term safety profiles of MXenes need to be thoroughly and extensively elucidated. They are being the first discovered and the most extensively characterized, many of the preliminary studies available focus on Ti_3_C_2_ biocompatibility. To date, there is no reported evidence of apoptosis or signs of cytotoxicity *in vitro* on cancer cells 182,194-196 and cultured neurons 197 from Ti_3_C_2_ exposure. Ti_3_C_2_ NSs injected *in vivo* in the bloodstream appear to be either excreted in the urine via physiologic renal clearance or retained in the tumor via the enhanced permeability and retention (EPR) effect, without evidence of accumulation in the major organs. Additional works report no sign of Ti_3_C_2_ NSs-induced red blood cell hemolysis *in vitro* 196, and similar findings have been reported for the cyto- and systemic biocompatibility for Ta_4_C_3_ 187,188 and Nb_2_C MXenes 189.

Finally, controlling the lifetime of the nanoagents in the body and mitigating the risks related to retention of nanomaterials and their byproducts, could significantly advance nanomaterial-based theranostic platforms in the translational pipeline. Recently, an active biodegradation scheme for Nb_2_C MXene NSs has been proposed 189. This process leverages human myeloperoxidase (hMPO), a free-radical species generating enzyme expressed by neutrophils to carry out their antimicrobial activity. In the presence of H_2_O_2_, hMPO generates hypochlorous acid and reactive radical intermediates, which degrade polymers and carbon-based materials. The incubation of Nb_2_C NSs in hMPO and H_2_O_2_ enriched medium for 24 h caused the complete degradation and disappearance of NSs, thus demonstrating *in vitro* the feasibility of this enzyme-triggered degradation route for MXenes.

#### 3.2.4. Other emerging 2D materials: graphdiyne, hexagonal boron nitride, silicene, antimonene, germanene, biotite, metal-organic frameworks, and layered double hydroxides

Beside the abovementioned 2DMs, it is worth considering other kind of emerging 2DMs with strong potential in the field of cancer theranostics. Considering the large number of 2DMs available, we have considered new 2DMs that are representative of less than the 6% of the works present into the literature: graphdiyne, hexagonal boron nitride, some members of the Xenes family such as silicene, antimonene (AM), and germanene 167, biotite, metal-organic frameworks (MOFs), and layered double hydroxides (LDHs).

***i)* Graphdiyne.** Graphdiyne is a carbon allotrope made of a combination of sp- and sp^2^-hybridized carbon atoms, which was first synthesized in 2010 198. This particular structure gives these material different physicochemical properties when compared to graphene, which is only formed by sp^2^-bonded carbons. It is characterized by uniformly distributed subnanometer pores, making GDY a potential candidate for applications in the area of clean energy as a new system for gas separation 199. From a biomedical point of view, GDY shows outstanding drug loading efficiency and high photothermal conversion ability, making it a promising tool for tumor-fighting. In this view, few recent works have started to explore its application for cancer treatment, such as the study of Jin* et al*., which applied GDY as a dual therapy platform for PTT and combined drug delivery of DOX *in vitro* and in mice 200. A very recent study of Li* et al*. also designed a specific GDY nanoplatform for cancer diagnosis as a photoelectrochemical biosensor for ultralow levels of cancer marker detection (microRNA let-7a) in human serum 201. In 2017, GDY was introduced in cancer theranostics as photothermal-acoustic wave nanotransducer for simultaneous PTT and PAI *in vivo* - this is the only study published in the field so far, to the best of our knowledge 202. Thanks to the PEGylation-enhanced biocompatibility, GDY was simultaneously exploited as a safe PTT agent and a PAI probe. Owing to the high extinction coefficient in the NIR region and the excellent photothermal conversion efficiency (42%), the nanoplatform showed an outstanding photoacoustic response and an ideal photothermal capability, exhibiting an efficient photothermal ablation of tumor* in vivo*.

***ii)* Hexagonal boron nitride.** Structurally, single-layer hexagonal boron nitride is an analog of graphene, where B and N atoms are bonded to form a hexagonal lattice. Despite the structural similarity, h-BN has very different properties compared to graphene: it is an insulator and has exceptional chemical and thermal stability, which make it an ideal encapsulating layer in 2DMs based devices 203,204. In the biomedical field, a great deal of studies focused on applications of boron nitride nanotubes, since their biocompatibility profile was more favorable compared to carbon nanotube counterparts 205,206. Thus, the interest in h-BN nanosheets for biomedical applications has emerged only in recent years, driven by the availability of highly concentrated h-BN dispersions in water 207. Yang* et al*. 208 first reported exfoliation of h-BN using pyrene derivatives in order to synthesize water dispersible and stable dispersions of h-BN. The method was further improved by McManus* et al*., which demonstrated highly concentrated dispersions with excellent biocompatibility profile in different cell lines up to a concentration of 100 µg/mL 209. Proof of concept studies reported the potential application of h-BN as a vector for the delivery of chemotherapeutic agents (e.g., DOX), oligonucleotides for immunotherapeutic purposes in cancers, and, recently, for the combined PTT and chemotherapy 210-213. Application of h-BN nanosheets in the field of theranostics is still in its infancy, with only one work proposing h-BN for theranostic applications 214 so far, to the best of our knowledge. In detail, Liu* et al*. developed a multifunctional CuPc@HG@BN theranostic platform composed of hexagonal boron nitride nanosheets (h‐BNNS), conjugated DNA oligonucleotide, and copper (II) phthalocyanine (CuPc). CuPc molecule in this construct plays a double role as a PS for PDT as well as a diagnostic tool for *in situ* monitoring and imaging of miR‐21 by surface‐enhanced Raman spectroscopy (SERS). miR-21 was selected as a target miRNA with expression levels significantly elevated in a variety of cancers, especially in breast cancer 214. The main reason for using h-BN nanosheets in this composite was to improve the SERS signal obtained from CuPc molecule. Interestingly, Raman signal of CuPc was negligible on the blank SiO_2_ while remarkable on h‐BN nanosheets and higher than the one on other 2DMs with the same number of layers, such as graphene and MoS_2_. The SERS signal enhancement was attributed to the interface dipole interaction between h‐BN nanosheets and CuPc induced by the highly polar B-N bond. Moreover, the intensity of CuPc was independent of the h‐BNNS thickness. Both *in vitro* and *in vivo* data demonstrated that this nanoplatform accumulates efficiently in tumor sites and has a remarkable therapeutic effect with minimized damage to healthy tissues 215.

***iii) Silicene.*** Silicene has attracted increasing attention in the theranostic field. It belongs to the family of Xenes, which are 2DMs with X being Si, Ge, Sn, Sb, etc. This crystal is characterized by a hexagonal honeycomb lattice presenting a non-planar buckled configuration. Thanks to its dimensionaliy, silicene stands out due to its oustanding properties, which include superconductivity 216, quantum spin Hall magnetoresistance 217,218 and chirality 219, to name a few examples. In a recent work in cancer theranostics, silicene was used as biodegradable phototherapeutic agent 220. In particular, the authors functionalized silicene with bovine serum albumin (SNSs-BSA) for cancer PTT and PAI of mice tumor xenografts. *In vivo* data demonstrated the efficiency of SNSs-BSA-based cancer PTT, due to the high photothermal conversion performances showed by the nanosystem and its resulting degradation accelerated by the photothermal activity. These outstanding properties, along with SNSs-BSA extraordinary photoacoustic contrast as well as intrinsic ambient degradability and biocompatibility, make silicene a promising multifunctional nanoplatform for photo-triggered therapeutics and diagnostic imaging.

***iv) Antimonene.*** Antimonene is a member of the Xenes with X=Sb, which attracted strong interest in the community due to its changes in electrical properties with decreasing thickness, from semimetal into semiconductor at the single layer 221,222. Indeed, AM is characterized by high thermal conductivity, superior stability, excellent carrier mobility, good strain-induced band transition, and unexpected spintronic properties 221-224.

In a recent study by Tao* et al*., antimonene was studied as a photothermal agent for minimal invasive and high selective PTT 225. The strategy adopted by the authors relied on the application of novel 2D AM quantum dots for cancer treatment. The nanosystem, synthesized by liquid exfoliation method and functionalised with PEG to enhance its biocompatibility and stability, exhibited a rapid NIR-triggered degradability and a noteworthy NIR-triggered tumor ablation *in vitro* and *in vivo*. Encouraged by these promising results, in a more recent study, the same group further explored AM as an innovative photonic drug-delivery nanosystem for cancer theranostics 223. PEGylated AM nanosheets have shown superior accumulation and penetration at tumor sites, but to their extraordinary photothermal properties, excellent DOX-loading capacity, controlled NIR light- and pH-triggered drug release, fully metabolic degradability, as well as multimodal-imaging properties (i.e., PAI/fluorescence imaging and PT imaging). The combination of these outstanding properties resulted in a noteworthy inhibition of tumor growth *in vivo* without recorded side effects and optimal clearance of the nanoplatform from the mouse body, suggesting promising applications in the field of cancer theranostics.

***v) Germanene.*** Germanene belongs to the Xenes family and shows promising properties for application in electronics 226,227. In the theranostic field, an innovative study has been successfully performed by Ouyang* et al*., leading to the development of functionalized 2D germanene quantum dots (GeQDs) 228. GeQDs were assessed to be excellent PTAs owning an outstanding photothermal conversion efficacy (superior to graphene and BP-based QDs) 225, higher stability 167, and optimal biocompatibility 225. Moreover, in order to define a complete multimodal nanosystem for cancer theranostics, beside the therapeutical aspects, GeQDs were proved to be useful also for multimodal diagnostic imaging (fluorescence/PAI /photothermal imaging-guided hyperpyrexia ablation of tumors).

***vi) Biotite.*** Biotite, or black mica (BM), consists of sandwiched sheets of silicate minerals offering great potential in the theranostic field. The unique structure of BM lays the foundations for the design of a multiple ROS-mediated combined PDT and chemodynamic therapy (CDT). Indeed, the BM-PEG NSs recently introduced by Ji* et al*. 229 served as robust theranostic nanoplatform for multimodal imaging-guided chemodynamic therapy, PDT and PTT. Thanks to the presence of MgO, Fe_2_O_3_, and FeO in the PEGylated BM structure, this new engineered theranostic system emerged as an intelligent tool with unique features. The BM-PEG NSs can be activated by a 650 nm laser, in order to produce anion superoxide ·O_2_- from molecular oxygen (O_2_), or by a 808 nm laser to induce local hyperthermia. Moreover, PAI as well as fluorescent and photothermal imaging capabilities of the engineered nanosystem enabled the multimodal imaging-guided breast cancer treatment.

***vii) Metal-organic frameworks.*** These materials are composed by coordinated metal cations or clusters, which are held together by organic bridging ligands, creating a porous structure 230-234. Typically, MOFs have a 3D structure, but they can also be made in a layered form, bridging the field of 2DMs. In the theranostic field these materials have shown exceptional promise 8.

For example, in a recent study, Zhao* et al*. tested a new theranostic MOF-based nanoplatform, where the material is used as both magnetic resonance-contrast agent and DOX carrier, enabling an improved therapeutic outcome compared to the one obtained with DOX alone 235. Following the same path, another group designed a MOF-based theranostic Fe_3_O_4_@UiO-66 core-shell for combined MRI and drug delivery 236, obtaining tumor eradication in both *in vitro* and *in vivo* studies. Another MOF-based tumor targeting drug delivery system with fluorescent properties was developed by Gao* et al*. allowing the guided-MRI of cancer 237. Further works regarding the application of imaging-trackable MOFs into PDT 238,239 and PTT 240 were carried out with positive results, exploring also MRI as a diagnostic modality combined with multiple therapies (i.e., drug delivery and PTT) in a single MOF-based system 241.

***viii) Layered double hydroxides.*** 2D structured layered double hydroxides have aroused extensive curiosity in the scientific community. New intercalative nanohybrids, such as inorganic-, organic- and bio-LDHs, have displayed extremely synergetic properties and complementary performances, being used first as antacid and anti-pepsin agent named “Talcid®” (Bayer) 242 and then as a biocompatible carrier/reservoir for drug and gene delivery 243. LDH is a class of inorganic 2D nanolayers 244,245 and one of the most commonly used nano-carriers in drug delivery systems. In the theranostic field, LDH-based nanohybrids, found a place for multimodal imaging in combination with different anticancer therapeutic approaches in order to realize synergistic treatments. LDHs have been widely studied as delivery carriers for theranostic approaches in different therapies, including drug and gene delivery, phototherapy and even immunotherapy 246-251. Following this line of research, Wang* et al*. 246 developed a Gd-doped layered double hydroxide (LDH)/Au nanocomposite for cancer bimodal imaging of and drug delivery. The new nanocomposite was shown to have a high DOX loading capacity and an interesting pH-responsive drug-release profile. Moreover, the nanosystem demonstrated a high performance *in vitro* and *in vivo* CT and T(1)-weighted MRI attitude without cytotoxicity or tissue damage. Another pH-responsive nanoplatform was tested by Huang* et al*. 247. The new MnFe-LDH releases paramagnetic Mn^2+^ and Fe^3+^ ions when in contact with the acidic microenvironment of solid tumors producing the enhancement of the T_1_ MRI contrast. In addition, the layered structure enables the delivery of chemotherapeutic drugs in a pH-controlled manner, and therefore it can simultaneously inhibit the growth of solid tumors.

Interestingly, Peng* et al*. 250 studied a DOX&ICG/MLDH composite material showing both pH-controlled and NIR-irradiation-induced DOX release, which included *in vivo* dual-mode imaging consisting in NIR fluorescence and MRI.

A step forward has been taken by Zuo* et al*. 251 with the delivery of dsDNA/siRNAs to Neuro-2a cells, thanks to the development of innovative manganese-based layered double hydroxide nanoparticles, explored for anticancer drug/gene delivery system and T_1_-weighted MRI for brain cancer theranostics.

In addition to imaging and drug delivery, Guan* et al*. 248 developed a NIR-sensitive layered supramolecular nanovehicle for a chemo-photothermal synergistic therapeutic agent. The particular structure of Gd-LDH not only stabilized ICG, enhancing the photothermal capacity, but also improved the recombination between electron and holes, generating more ROS under NIR irradiation.

## 4. The importance of 2D material characterization for theranostics

Characterization of nanomaterials is of fundamental importance for the successful development of commercial applications 252, in particular in the context of human health and safety because the biological and toxicological properties of the nanomaterial are directly determined by its structure.

In the case of 2DMs, the importance of characterization has been recognized as a high priority by the community 253-256: this is because different forms of graphene-based materials - each of them with its own properties - can be made, depending on the production method and processing used. For example, using graphene oxide provided by different commercial sources may give different results because it is produced using different protocols, giving rise to materials with a different structure (e.g., C/O content), which will behave differently in the biological environment (see stability, degree of functionalization, etc). A change in the exfoliation parameters used to produce graphene by liquid-phase exfoliation can provide graphene with different size, thickness or defects concentration 256. Similar issues can be extended to other 2DMs. Thus, it is of primary importance to determine the nature of the material produced and how this changes during the processing. In the case of biomedical studies, as the nanomaterial is often functionalized to achieve improved stability or biocompatibility or it is loaded with NPs or drugs, used for imaging or to deliver the therapy, a careful analysis of the properties from synthesis to the final vector is of crucial importance.

**Figure 3:**
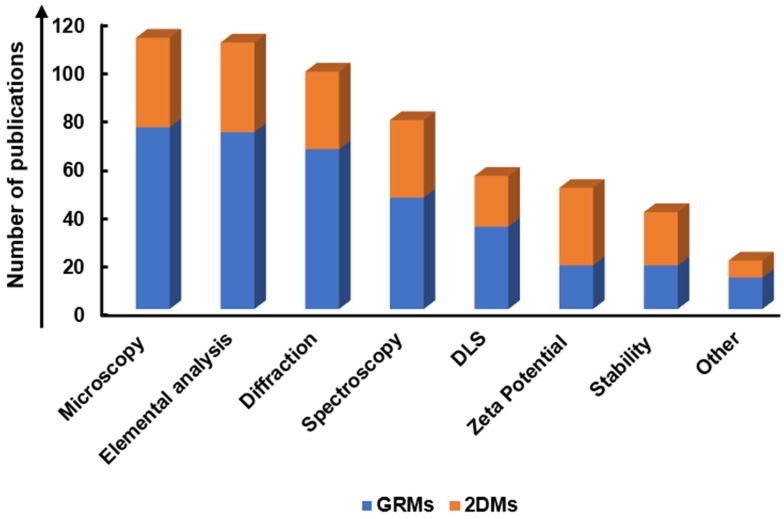
Analysis of the literature based on methods of material characterization for GRMs and other 2DMs.

Recently, the community has introduced some guidelines to establish universal practices to allow a better classification of solution-processed 2DMs, based on specific parameters, such as thickness, size, and surface chemistry 253. However, characterization of solution-processed 2DMs is currently very challenging because dispersions typically contain nanosheets with a wide distribution in size and thickness, in contrast to traditional colloidal dispersions. Thus, a combination of different techniques (**Table 5A, Table 5B**), ranging from nanoscale to microscale resolution, must be used to characterize solution-processed 2DMs. Furthermore, due to the different nature and dimensionality of solution-processed 2DMs, characterization methods typically used for colloids may have limited validity, when applied to 2DMs. In this section, we provide an overview of the most used techniques for characterization of 2DMs used for theranostics (**Table 5A, Table 5B**). The following sections are dedicated to the different characterization methods (and refer to **Figure 3** for an overview).

***i) Microscopic techniques for morphology characterization***: Microscopic techniques such as atomic force microscopy (AFM) and transmission electron microscopy (TEM) are typically used for size and thickness measurement of 2DMs. In case of AFM, by depositing the nanosheets on a flat substrate, such as on silicon wafer, the morphology of individual nanosheets can be directly measured in sub-nanometer scale resolution to obtain statistical analysis of size and thickness distribution of nanosheets. It is recommended to scan different area on the same sample in order to achieve a realistic distribution of the size and thickness of the flakes. However, in the case of solution-processed 2DMs, adsorption of solvent molecules or chemical modification of the surface of 2DM can lead to overestimation of the thickness (number of layers) of the nanosheets 256. Thus, AFM can be used only as a qualitative method for thickness identification. TEM, on the other hand, can be used to directly count the number of layers by analyzing the flake edges and characterize the structure at sub-nanometer resolution in order to identify possible defects 257. Low-resolution TEM can be used to look at the shape of the flakes and also to qualitatively identify the thickness by looking at the contrast. However, TEM is very time consuming and needs to be performed on at least 100 nanosheets. Many works show TEM pictures of 1-2 flakes, which may not be representative of the whole material in dispersion. Furthermore, TEM can damage the material during measurement due to the use of highly energetic electron beams (typically of a few hundred kV acceleration voltages) at high-pressure vacuum. These microscopic techniques are beneficial to study the morphology of 2D material hybrids, for example, in the case of QDs or other nanomaterials deposited or grown directly on the 2DM surface, due to their very high resolution. In this case, it is crucial to determine the coverage of the loaded drug or nanomaterial on the nanosheet, e.g., if there are isolated NPs or aggregates, and if the coverage is similar for all flakes. A few studies have used scanning electron microscopy (SEM) to characterize the nanosheets morphology, but the resolution of this technique is not as good as that of AFM or TEM. Thus, the smallest flakes cannot be resolved. Furthermore, SEM requires conductive samples. In the case of non-conductive nanomaterials, deposition of a metallic film is required, sometimes making more difficult to assess the morphology of individual flakes.

***ii) Optical spectroscopic techniques***: Several absorption/emission spectroscopies, such as Fourier Transform Infrared (FT-IR), UV-Visible, and Raman spectroscopy are often used as a simple, non-invasive technique to characterize the structure and changes in chemical composition and/or degree of conjugation *in-situ*. FT-IR is typically used to confirm functionalization with polyethylen glycol (PEG), which has been used in many works to improve water stability of the nanomaterial. In the case of graphene oxide, both FT-IR and UV-Vis can be used to confirm the reduction of the material: in the FT-IR spectrum, the intensity of the peaks associated to the carbonyl, carboxyl, hydroxyl and epoxy groups will decrease in intensity. In the case of UV-Vis spectroscopy, the absorption spectrum of graphene oxide shows a peak at ~250 nm, due to π-π* transitions, which moves to slightly higher wavenumbers for reduced-graphene oxide (rGO). However, this analysis is only qualitative and requires further characterization in order to measure the C/O ratio.

UV-Vis spectroscopy is also a potent technique for the structural characterization of semiconducting 2DMs, such as TMDs, due to their unique exciton's features, which sharply change depending on defects formation, functionalization or polymorph change. One can note that Li-intercalation has been used very often to produce TMDs dispersions: this method is well known to produce a substantial change in the structure of the material, which changes from 2H to 1T polymorph, leading to metallic properties. This fact should reflect in changes in the absorption spectrum and also changes in the photoluminescence and fluorescence of the hybrid material, which is of fundamental importance in theranostics. Recent studies have also proposed protocols based on absorption spectroscopy to identify size and thickness distribution of such materials 256. It is noted that UV-Vis spectroscopy is of fundamental importance in theranostics as it is used to confirm strong absorption of light. Photoluminescence and fluorescence are also used for determining the theranostics effects - as such, we did not consider these features in the list of characterization techniques.

Raman spectroscopy is one of the most popular techniques to characterize graphene and other 2DMs 258. It has been shown that Raman spectroscopy can be used to identify graphene 259, to determine type and concentration of defects 260-262, amount and type of doping 263 and many other properties. However, these studies have been conducted on a high-quality graphene nanosheet, produced by mechanical exfoliation. Solution-processed or chemically modified graphene materials, produced by different methods, do show very different Raman features because the spectrum (i.e. the intensity of the peaks, their full width and half maximum, FWHM, and positions) is strongly sensitive to the presence of adsorbed or intercalated molecules and to morphological changes, such as restacking of the flakes, wrinkles, folding, defects, strain, and so on. Thus, the Raman analysis typically performed on perfect and clean graphene flakes produced by mechanical exfoliation cannot be extended to solution-processed graphene. For example, the FWHM of the 2D peak is used as a fingerprint to identify single-layer graphene (~25 cm^-1^) 259- however, the typical FWHM of the 2D peak of solution-processed graphene is around 50 cm^-1^.

Despite these limitations, Raman spectroscopy can still provide qualitative information on the thickness distribution of the flakes produced by liquid-phase exfoliation, as reported in 264-267, by using a protocol based on the quality of the 2D peak fitting. Note, however that this method is very time-consuming as it requires analyzing 50-100 isolated flakes, which could be hardly visible under the optical microscope, and cannot be applied to GO and rGO and solution-processed graphene produced by other methods. In the case of GO and rGO, these materials are defective, hence the Raman spectrum sharply changes and therefore Raman spectroscopy cannot be used to identify the number of layers. Despite this limitation, Raman spectroscopy can still provide useful information regarding number of defects and to confirm reduction of GO. Defects analysis is typically done by using the intensity ratio between D and G peaks (I(D)/I(G)). However, this analysis needs to be done with care: first, I(D)/I(G) can be compared to other values reported in literature only if measurements are performed at the same excitation wavelength, as this parameter strongly changes with the laser excitation 268; second, I(D)/I(G) do not always increase for increasing defects concentration. Graphene shows a two-stage defects trajectory 262: in Stage 1, starting from pristine graphene, the Raman spectrum evolves as follows: the D peak appears and I(D)/I(G) increases; the D', another defect activated peak located at ~1600 cm^-1^, appears; all the peaks broaden and G and D' begin to overlap. In this stage, I(D)/I(G) can be used to estimate the amount of defects 269,270, while I(D)/I(D') can be used to distinguish between different type of defects 270. At the end of Stage 1, I(D)/I(G) starts decreasing. As the number of defects keeps increasing, the Raman spectrum enters Stage 2, showing a marked decrease in the G peak position and increase broadening of the peaks; I(D)/I(G) sharply decreases towards zero and second-order peaks are no longer well defined. Thus, I(D)/I(G) needs to be coupled with another Raman fit parameter, such as the FWHM of the D, G or 2D peak, in order to distinguish between the two-stages. Note that GO belongs to stage 2- defective graphene, so reduction of the material should lead to a decrease of the peaks FWHM and to an increase in I(D)/(IG), assuming rGO to remain in stage-2.

Similar mistakes done in the analysis of the Raman spectrum of graphene can be seen also for other 2DMs, where the Raman spectrum of a solution-processed nanosheet is compared to the Raman spectrum of a single-layer 2DM produced by mechanical exfoliation. Changes in FWHM and positions of the peaks in a spectrum from solution-processed material may not necessarily be related to changes in thickness.

Because of the limits and challenges with optical spectroscopic techniques, these measurements should always be complemented with microscopic techniques. Note that since the resolution of optical spectroscopic and microscopic techniques is very different, one should not expect to get exactly the same results. For example, if Raman spectroscopy shows that the dispersion contains mostly single and few layers graphene, this should be confirmed by TEM as well, although the numerical distribution may be slightly different.

It is pertinent to note that Raman spectroscopy, being so sensitive to changes in the structure, is an ideal technique to investigate the changes in the properties of the nanomaterial after each functionalization step. For example, if the Raman spectrum of the final vector does not show any G peak, this would possibly indicate the almost complete disappearance of graphene.

***iii) X-ray diffraction (XRD)***: XRD is often used for qualitative identification of the crystal phases from the study of the diffraction angle 271. For graphene and other 2DMs, XRD is often used to measure the *d*-spacing between the layers (2θ = 26.3° for graphite (002) peak and 2θ = ~10 - 11° for GO) 272,273, degree of oxidation of GO (decreasing 2θ value and peak broadening observed with increasing degree of oxidation) 274 or confirmation of conjugation with other materials. Note that XRD is a “bulk characterization technique”, providing average information on all material, while Raman spectroscopy can be used on individual nanosheets.

***iv) X-ray photoelectron spectroscopy (XPS)***: XPS is a surface-sensitive, quantitative spectroscopic method that is often used to study the elemental and chemical composition of materials 275. Oxygen species of GO and rGO, and other elemental peaks from functionalization can be quantitatively characterized by XPS to estimate the degree of oxidation, reduction and/or functionalization 276. XPS can be a powerful technique to quantitatively determine the degree of oxidation or functionalization, but the measurement should be carefully carried out since it is highly surface-sensitive technique (measurement depth is usually a few nanometers). Moreover, possible contamination during the sample preparation and/or measurement should be carefully avoided, or possible adsorption of adventitious carbon from the atmosphere should be taken into account during analysis. Finally, XPS cannot be made on individual flakes, so it requires producing a film or a membrane. Thus, XPS provides average information on all material. A similar technique is given by Energy-dispersive X-ray spectroscopy (EDX, EDS or XEDS), which allows analyzing much smaller material size, but needs to be carried out in a scanning electron microscope.

***v) Thermogravimetric analysis (TGA)*** is often used to determine the weight percentage of oxygen species, graphitic material, and/or degree of conjugation with additional materials.

***vi) Zeta potential and Dynamic Light Scattering (DLS)***: the zeta potential is the measure of surface charge and potential distribution of a nanomaterial in solution. In some cases where the particles are electrostatically stabilized in a colloidal suspension, the zeta potential can be correlated to the sedimentation behavior 277. For graphene-related materials, the zeta potential can be used to measure the degree of oxidation and functionalization, if the charge of the functionalized material is known. DLS is a simple and fast technique to measure the hydrodynamic size of nanomaterial dispersed in a solution by using light scattering. Correlative relationship between the size measured by DLS and microscopic techniques has been shown 278, but compared to microscopic techniques, DLS is a crude method to measure the size of 2DMs as the flakes will be always crumpled into arbitrary shape in solution. Moreover, since DLS technique depends on the intensity of scattering for size measurement, larger particles can have more influence on the calculated average than smaller particles, which can lead to overestimation of size measurement, especially for solutions involving wide distribution of particle sizes, such as in graphene dispersion 279.

## 5. Comparing theranostic approaches and results: a critical analysis

**Figure 4:**
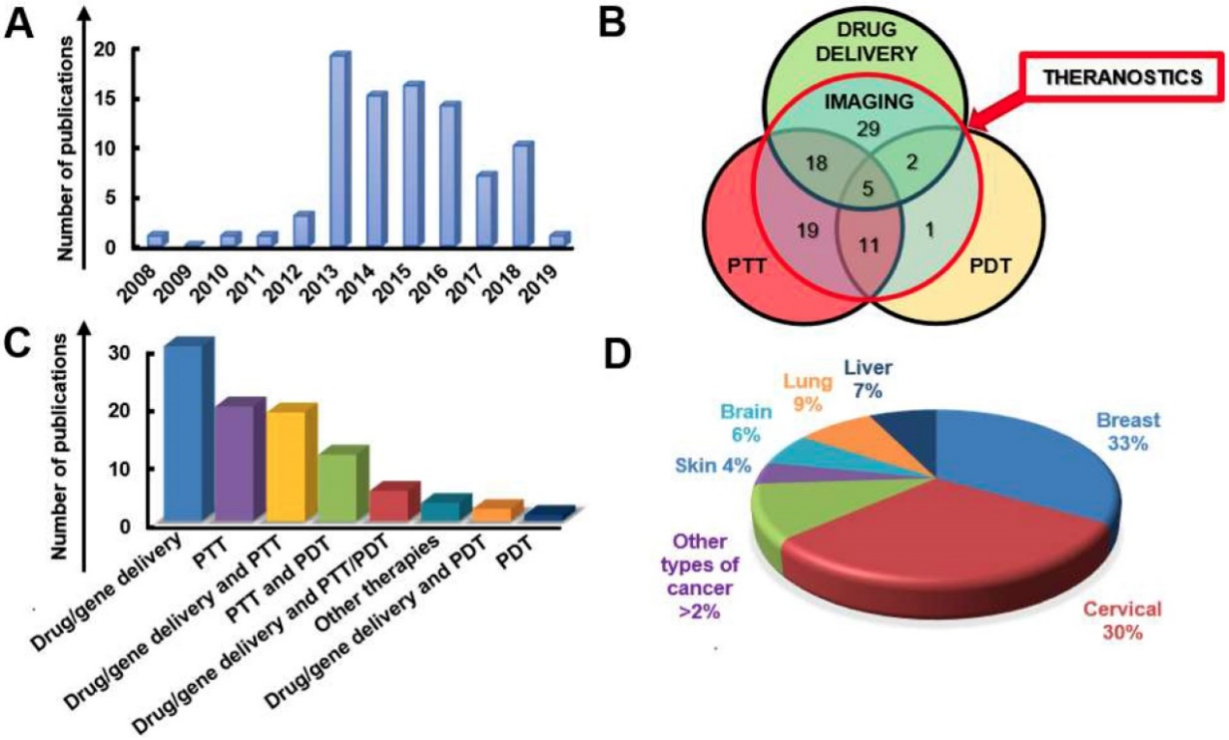
Analysis of the literature concerning GRMs. (A) The number of publications on graphene and GRMs in cancer theranostic (from 2008 to 2019). (B) Venn diagram based on the combination of imaging and the main therapeutic applications (drug delivery, PTT, and PDT) using GRMs as cancer nanotheranostic tools. (C) Histograms showing the numbers of publications concerning GRMs in cancer theranostics based on the different therapeutic approaches used in combination with imaging. (D) Overview on different types of cancer treated with GRMs as nanotheranostic tools (other types of cancer include: renal, bone, hepatic, Burkitt's lymphoma, ovarian, pancreatic, sarcoma, colon, and prostate).

**Figure 5:**
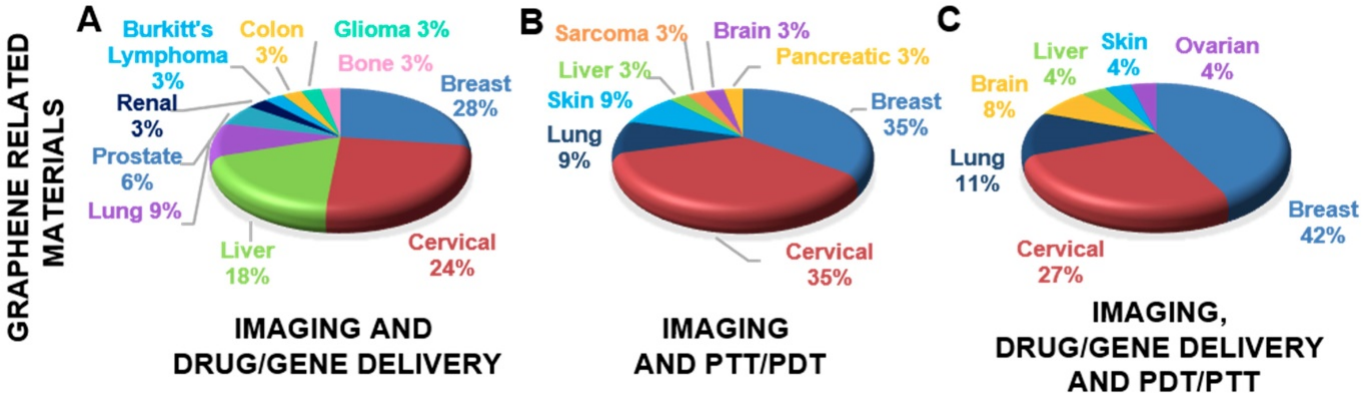
Percentages of various types of cancer treated with different combinations of theranostic approaches using GRMs. (A) Overview of different types of cancer treated with imaging and drug/gene delivery. (B) Overview of different types of cancer treated with imaging and PTT/PDT. (C) Overview of different types of cancer treated with imaging, drug/gene delivery, and PTT/PDT.

**Figure 6:**
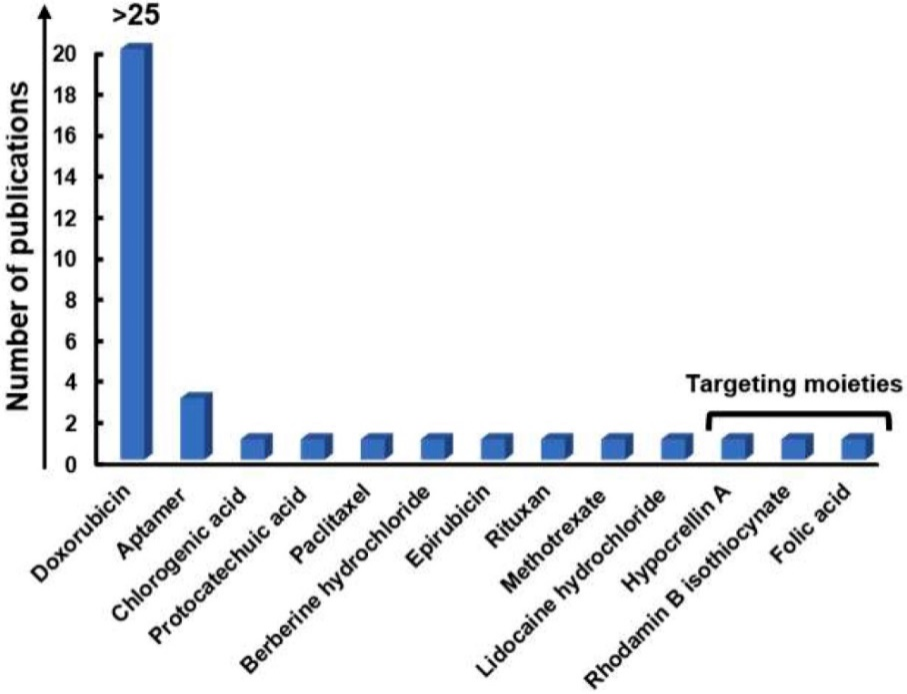
Number of publications related to the different drugs used for drug delivery based on GRMs, including targeting moieties.

Through our review of the literature we found 84 publications concerning graphene, GRMs, and graphene hybrids (**Figure 4A**), which are summarized in **Table 1**, **Table 2** and** Table 3** according to the different type of combined applications, cancer, species of investigation, model as well as material and relative functionalization. Most of the cited studies implied the use of imaging (MRI, X-ray CT, fluorescent imaging, PAI, photothermal imaging, ultrasonography, and PET) as a diagnostic tool, combined to one or more therapeutic strategies at the same time as shown in the Venn diagram (**Figure 4B**). Focusing on the type of application, we found that, among the different theranostic protocols, the combined use of imaging and drug or gene delivery, represents the majority of the analyzed studies (31%). The second most commonly used method is represented by combined imaging, drug delivery and PTT (24%); followed by combined imaging and PTT (23%); imaging PTT and PDT (11%); imaging, PDT, PTT and drug delivery (5%); other therapies (3%); imaging, PDT and drug delivery (2%); and imaging and PDT (1%) (**Figure 4C**). From the analysis of the literature concerning the different types of cancer treated with GRMs as a theranostic tool, breast and cervical cancers appear the most studied (34% and 30% of the studies, respectively), followed by lung, liver and brain cancer, which appeared approximatively in the same number of publications (8%), and skin cancer (4%). Other types of cancer (renal, bone, hepatic, Burkitt's lymphoma, ovarian, pancreatic, sarcoma, colon, prostate) represented the focus for less than the 2% of the papers (**Figure 4D**). In particular, even by looking at the different types of therapeutic approaches and the combinations of strategies, the majority of the papers involving imaging and drug/gene delivery or PTT/PDT were carried on breast and cervical cancer models, independently from the type of approach (**Figure 5**). Focusing on the different molecules used for drug delivery applications of GRMs for cancer theranostic, DOX emerged as a first-choice drug to functionalize as a common therapeutic drug (**Figure 6**). This fact opens the window on the multitude of emerging new drugs never conjugated with 2DMs, therefore, offering the possibility of more innovative functionalizations perhaps able to reduce possible side effects and drug doses.

**Figure 7:**
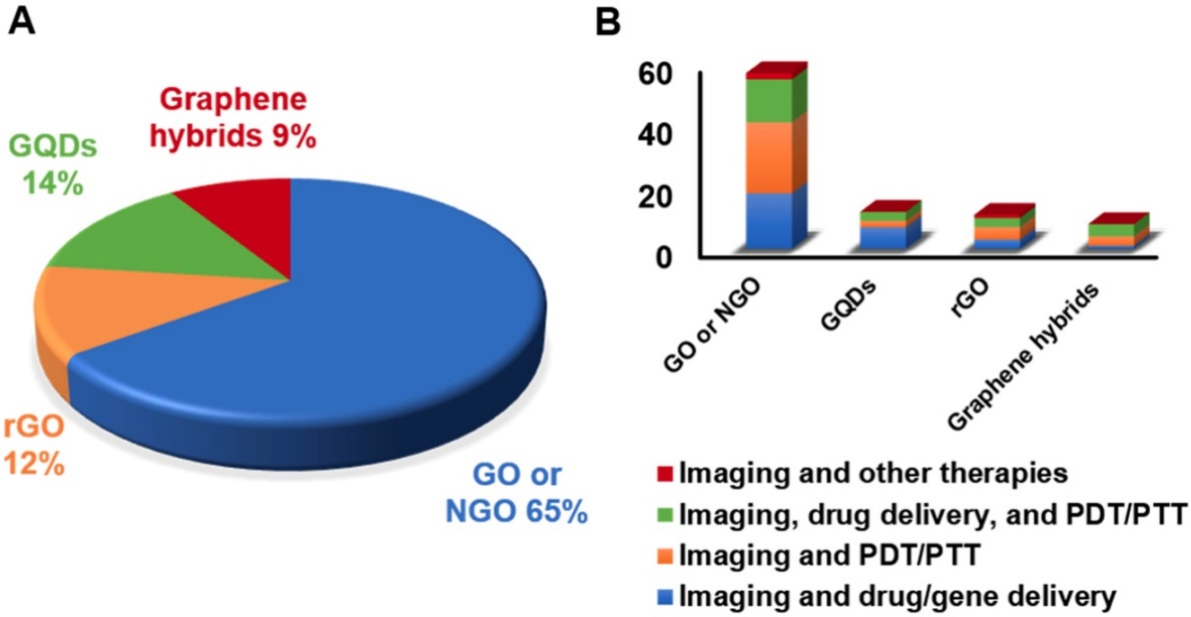
Analysis of the literature concerning the type of GRMs. (A) Percentages of the different types of GRMs used in theranostics. (B) Overview of different types of GRMs and their theranostic application.

Comparing the reports present in the literature concerning the use of graphene for cancer theranostics, GO emerges as a first-choice material for the design of nanotheranostic tools in 65% of the studies, followed by GQDs (14%), and rGO (12%), while only 9% of the tested materials included graphene or other graphene hybrids (**Figure 7A**). This view is mainly due to GO superior biocompatibility, the ease of functionalization, due to the considerable presence of chemical polar groups, as well as high dispersibility in aqueous biological environments and prolonged blood circulation time compared to other GRMs 280. In particular, the high surface-to-volume ratio allows the conjugation with different molecules able to improve and expand the range and effectiveness of simultaneous imaging methods and multiple therapies applicable in the same nanotheranostic tool. Indeed, the diagnostic and therapeutic capability can be improved by the conjugation of the material with PSs, genes, and drugs for enhanced hyperthermia, gene therapy, and anticancer drug effectiveness. Therefore, not surprisingly, a good portion of the studies, where GO or NGO have been exploited for imaging application, is associated with drug/gene delivery alone or in combination with PDT/PTT (**Figure 7B**).

However, rGO resulted in one of the most effective materials in terms of tumor reduction ability, demonstrating to be able to totally ablate the tumor *in vivo* 281. This seems to be due to its physicochemical properties, such as the strong NIR absorbance capability compared to GO and other GRMs, endowing it with a high photothermal conversion efficiency for cancer thermal ablation 282. The number of publications focusing on the use of rGO for imaging application associated to PDT /PTT, alone or in combination with drug/gene delivery, is much higher compared to the use of rGO for imaging and drug/gene delivery alone (**Figure 7B**). Indeed, in several studies, rGO has shown to be an appealing candidate for controlled irradiation-responsive nanoplatforms. Moreover, its therapeutic properties can be enhanced thanks to the association with noninvasive temperature-dependent drug release in response to illumination or pH-depended drug release in response to acidic conditions similar to those of cancer cells 51,283. The ability of rGO to absorb NIR light and convert it into heat can also be exploited for PAI, due to the consequent ultrasonic emission, resulting in a theranostic nanoplatform with dual-enhanced photothermal conversion properties 89,114. In addition, rGO displayed magnetic properties and was able to improve the contrast in both MRI and X-ray CT 284. This scenario, is of particular interest in theranostics, since MRI represents the most powerful imaging method for tumor diagnosis and early detection 285.

Considering GQDs, they were mainly exploited for imaging properties, due to their intrinsic photoluminescence, making them ideal candidates for imaging purpose, but were also used in conjugated forms for drug/gene delivery (**Figure 7B**). However, better results with GQDs in terms of cancer ablation seems to be obtained through the association of imaging with PTT or PDT, alone or in combination, leading to considerable (53.4%) 83 or even total 97,171 tumor eradication. However, the effects showed different potency also dependently on the different cell ability to internalize the materials and their susceptibility to reactive oxygen species 121. These results suggest that these aspects should be taken into account given future clinical translation, in accordance with the different type of cancer to be treated. In general, promising results were obtained by both *in vitro* and *in vivo* studies, however, animal models showed a more variable response, obtaining a total cancer eradication only in 20 treatments 74,80,84,87-90,93-97,123,281,286-291 out of 59. Of these successful studies, only two were carried out only using gene/drug delivery while all the others exploited PTT or PDT alone or in combination with gene/drug delivery. A summary of the advantages and disadvantages of GRMs for cancer theranostic applications emerged from the reported studies is illustrated in **Table 6**.

**Figure 8:**
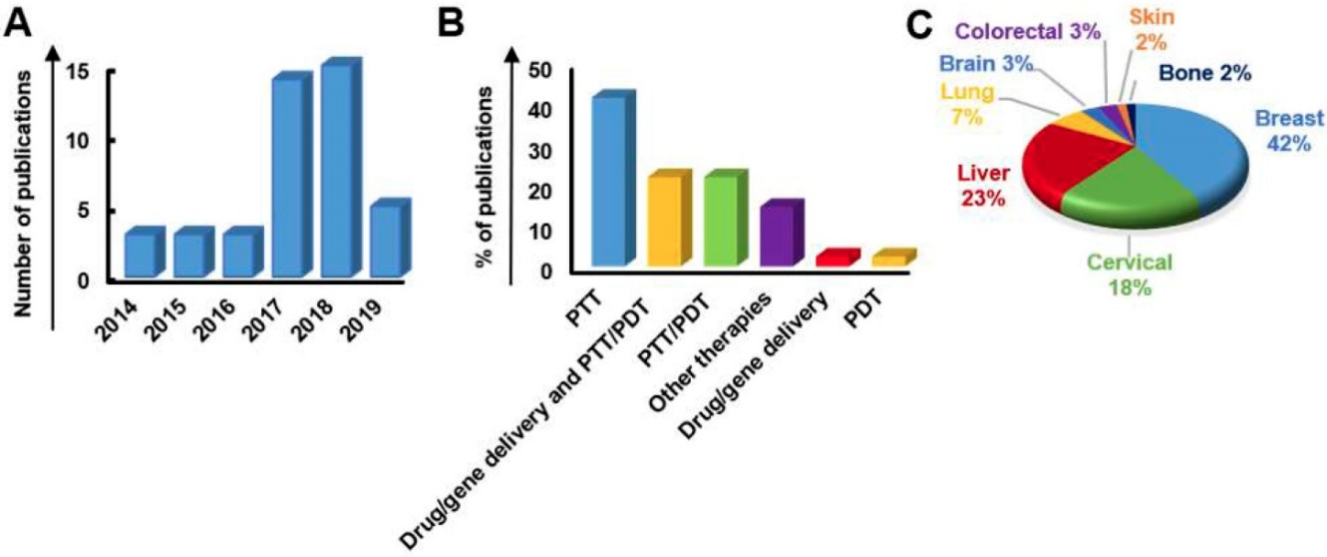
Analysis of the literature concerning 2DMs beyond graphene. (A) The number of publications of 2DMs in cancer theranostics. (B) The number of publications concerning 2DMs in cancer theranostics based on the different therapeutic approaches used in combination with imaging. (C) Overview of different types of cancer treated with 2DMs.

Concerning the analysis of 2DM beyond graphene, it is clear that they are rising increasing interest; as shown by the recently growing number of publications based on these promising tools for cancer theranostics (**Figure 8A**).

As shown in **Figure 8B** these materials were mainly employed for imaging in association with approaches involving PTT alone (41%) or in combination with drug/gene delivery (22%), and PDT (19%). By the analysis of the literature concerning the different types of cancer treated with 2DMs beyond graphene as a theranostic tool, breast and liver cancers appear the most studied (42 and 24% of the studies, respectively), followed by cervical (18%), lung (7%), brain (3%), while only a small portion of works involved colorectal, skin and bone cancer, which appeared approximatively in the same number of publications (2%) (**Figure 8C**).

Although the applications of new emerging 2DMs are still rare in the field of cancer theranostics and biomedicine in general, from the works here analyzed it is possible to comment on the merits and potential clinical translation of those new materias. Among new emerging 2DMs, antimonene QDs and GDY exhibit a photothermal conversion efficacy (45.5 and 42%, respectively) higher than traditional 2DMs, such as graphene, GO, MoS_2_, and BP 225. This property makes these emerging nanomaterials particularly suitable for PTT. GDY has also shown to exceed pristine graphene nanosheets in terms of drug loading ability, with a DOX loading content equal to 38% 292, due to its structure, composed by both sp- and sp_2_-hybridized carbon atoms, allowing GDY to preserve its conjugated structure even after covalent functionalization. This ability shows promises in the field of nanotheranostic, since it could allow simultaneous covalent link of several molecules, including drugs and targeting, tracking or bioactive structures.

MXenes in general possess remarkable photothermal performance 194,293. Ti_3_C_2_ nanosheets are characterized by an exceptional high extinction coefficient of 25.2 L g^-1^ cm^-1^, compared to that of GO nanosheets (3.6 L g^-1^ cm^-1^) 294. However, their relatively low photothermal conversion efficiency could limit their future applications. The development of Ta_4_C_3_ allowed to obtain both optimal photoabsorpion properties and photothermal conversion efficiency (44.7%) thanks to the strong absorption band, similar to those of other conventional 2DMs, such as graphene 110 and MoS_2_ 152.

Compared to other types of 2DMs, such as MoS_2_ 147, BP 295, graphene 110,294, Ti_3_C_2_, and Ta_4_C_3_ 296, Nb_2_C exhibits a strong and almost constant optical absorption in the NIR I and NIR II windows. The possibility of employing PTA with high conversion efficiency also in the NIR II window (1000-1300 nm), such as Nb_2_C, is expected to attract increasing interest in the future of cancer therapy, allowing a higher tissue penetration depth.

It is clear that the physicochemical properties of 2DMs are urging their use in biomedicine 297. For instance, thanks to the higher water dispersibility and colloidal stability, g-C_3_N_4_ and BP are supposed to be more advanced in the biomedical field, without the use of any surfactants. In contrast, despite their limited stability in aqueous dispersions 298, electrically inert h-BN sheets seem to be more biocompatible and better suited for the drug delivery. With the aim to improve the biocompatibility of 2DMs as well as to control their solubility and biodistribution, the development of new covalent functionalizations will be an interesting effort for future studies. Moreover, following this path, the syntheses for theranostic applications needs to focus on different parameters that could dramatically change the fate of the new synthetized 2DMs: dimensions, surface functionalities, and aqueous dispersibility.

**Figure 9:**
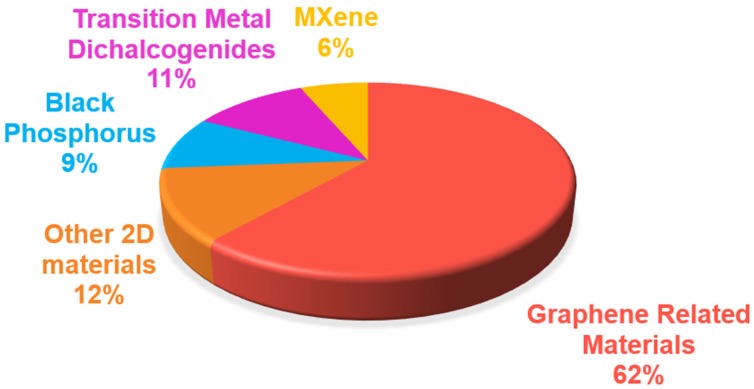
Graphic representation of the discussed works concerning the use of GRMs and other 2DMs in cancer theranostics. “Other 2D materials” includes: graphdiyne, hexagonal boron nitride, silicene, antimonene, germanene, biotite, metal organic frameworks, and layered double hydroxide.

**Figure 10:**
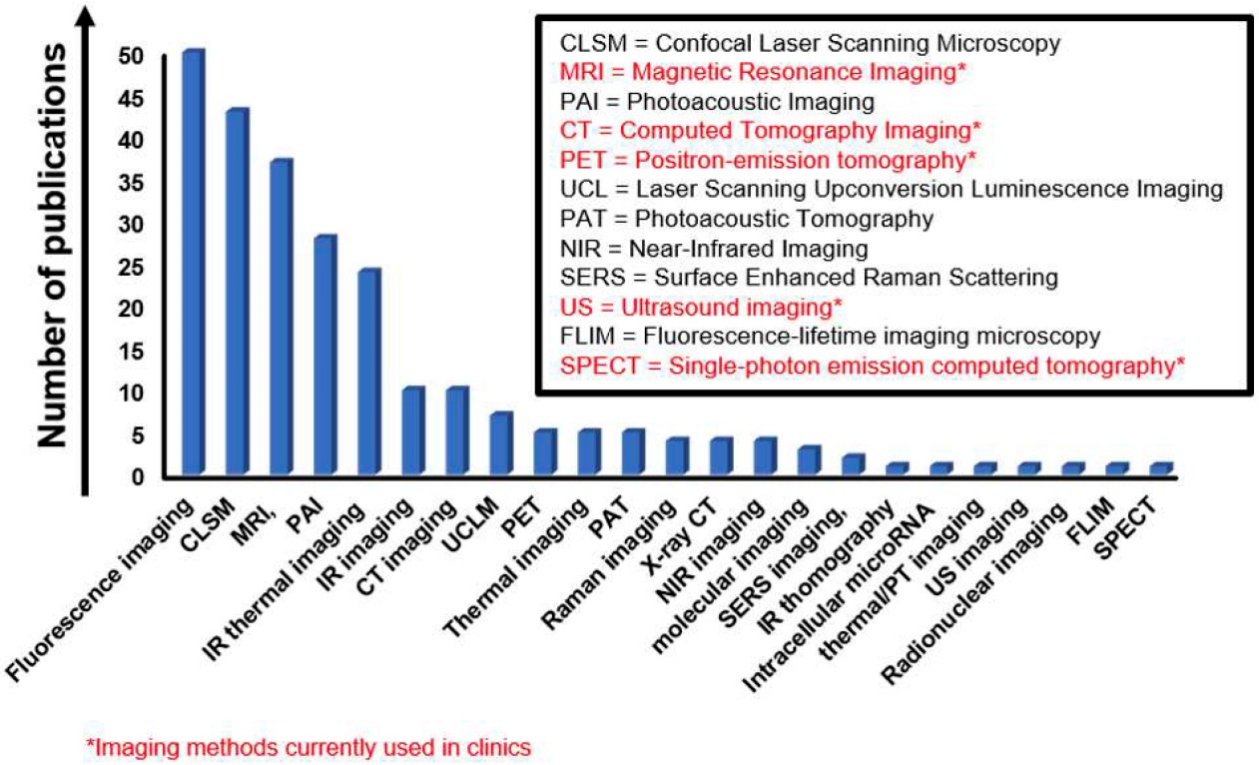
Overview of different types of imaging modalities used for theranostic purposes (GRMs and 2DMs). Abbreviations: CLSM = Confocal Laser Scanning Microscopy, CT imaging = Computed Tomography Imaging, FLIM = Fluorescence-lifetime imaging microscopy, MRI = Magnetic Resonance Imaging, NIR imaging = Near-Infrared Imaging, PAI = Photoacoustic Imaging, PAT = Photoacoustic Tomography, PET = Positron-emission tomography, UCL = Laser Scanning Upconversion Luminescence Imaging, SERS imaging = Surface Enhanced Raman Scattering, SPECT = Single-photon emission computed tomography, US imaging = Ultrasound imaging.

**Figure 11:**
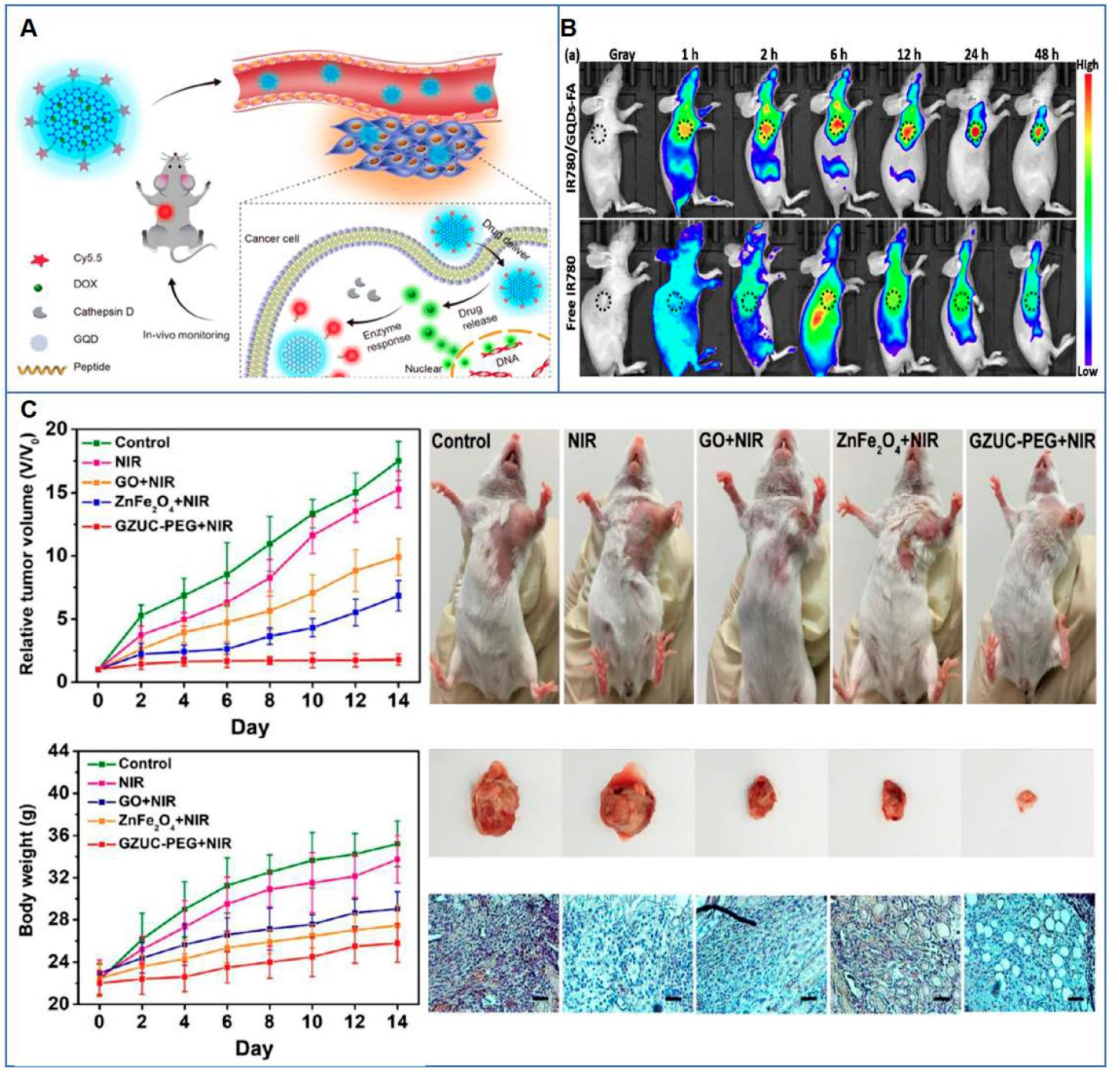
Examples of GRM-based imaging and therapy for combined and multimodal applications in cancer theranostic. (A) Schematic illustration of drug delivery theranostic strategy based on GQD fluorescence for a programmatic monitoring of the anticancer drug delivery, release, and response. Adapted with permission from 56, copyright 2017 American Chemical Society. (B) Theranostic application of GQDs fluorescence; NIR fluorescence imaging of tumor-bearing mice intravenously injected with free IR780 or IR780/GQDs-FA up to 48 h. Adapted with permission from 288, copyright 2017 American Chemical Society. (C) Tumor photographs, histological images, tumor volume and body weight of mice trated with GO-based nanotheranostic tool. Adapted with permission from 85, copyright 2018 American Chemical Society.

Overall,** Figure 9** shows a comparison among the percentage of different type of materials (GRMs and new 2DMs beyond graphene) used in cancer theranostics. On the whole, by an analysis of the imaging approaches, the most used imaging technique was CLSM (42 papers), followed by fluorescence imaging (41 papers), MRI (37 papers), and PAI (28 papers), while other imaging methods were less frequently used (**Figure 10**). As mentioned before, even if in several studies, the fluorescence properties are claimed as possible diagnosis strategies, it is important to mention that fluorescence imaging has not entered the clinic yet. Some outstanding examples are reported in **Figure 11** which summarizes some of the most complex and multimodal applications of these nanotools available to date.

## 6. Conclusions: how to develop 2D materials for theranostics

### 6.1. Targeting and resolution: two historical limits to consider

The recent advances in new 2DM development were directed to overcome some of the persisting limitations of conventional cancer-fight modalities such as: i) the nonspecific targeting and ii) low resolution in imaging and difficulties in diagnosis.

***i) The nonspecific targeting.*** One of the main limitations of current chemotherapeutic drugs concerns non-specific targeting, which is especially relevant for drugs with a broad range of targets that could lead to severe organs damage 16. On the other hand, the delivery of therapeutic molecules could be difficult for those drugs that can hardly penetrate tumor cells through the blood or showing a short circulation half-live 299.

The delivery of drugs at the requested site by nanomaterials is still challenging, even for FDA approved particles. Notably, it has been determined that less than 1% of NPs explored for anticancer drug delivery typically reach the tumor 300. It is far too early to conclude if 2DMs have a chance of entering the clinic for drug delivery, and eventually to perform better than current clinically approved nanomedicines 301-304. Undoubtedly, the high surface-to-volume ratio makes 2DMs very useful in this sense allowing a high load of drugs and the functionalization with specific ligands recognized by particular receptors overexpressed on specific tumor cells, facilitating the selective targeted process of internalization and the delivery of chemotherapeutic agents or genes up to the action site. Furthermore, the multiple functionalizations are not inside of the particles as it can happen with multistage vectors 305. The drugs in the case of 2DMs may have an easier way of release. However, on the other hand, multistage anticancer vectors can incorporate several components, these components are activated sequentially in order to successively address transport barriers; this “multistage advantage” has not been reported yet for 2DMs. Microenvironmental priming strategies in the theranostics context of 2DMs are highly desired. Moreover, a polymer coating would allow the carrier to circulate unnoticed towards the immune system, while the cargo release could be programmed according to the presence of various stimuli, such as pH and temperature variations, that typically occur into the cancer tissues. To overcome the issue concerning the lack of target specificity, further information concerning the biodistribution of nanomaterials in the context of cancer theranostic research are also still needed. Indeed, toxicological studies concerning GRM biodistribution investigation have highlighted the impact of critical aspects on nanomaterial fate into the body, including physicochemical properties, such as dimension and functionalization, as well as the formation of protein corona 306-309. Moreover, a key aspect still not considered in the studies analyzed here and that could influence the biodistribution of nanomaterials, is the difference between healthy and cancer blood vessels 310. Indeed, the latter is characterized by slower blood flow and the presence of holes (fenestration) that should be taken into consideration in 2D design and production to predict the possible extravasation to reach the tumor region 302.

***ii) Low resolution in imaging and difficulties in the diagnosis.*** The low-resolution performance in imaging represents one more hurdle for cancer theranostics development. The suitability of the promising 2DMs here discussed to serve as imaging tools for cancer nanotheranostic purpose mainly relies on their outstanding physicochemical characteristics useful for cancer diagnosis and guided therapy, as reported in all the studies analyzed. For example, GO exhibit fluorescence from the visible to NIR range and NIR fluorescent dyes to reduce the intrinsic graphene quenching effects. However, while some of the imaging opportunities offered by nanomaterials appear to be more easily and readily applicable to the clinic, such as the implementation of MRI- and CT imaging-based technologies, which interact mainly at the atomic level allowing the visual inspection at the organ length scale, other optical techniques exploitable at the molecular level by these nanosystems (e.g. fluorescence-based imaging for diagnosis and guided therapy) face hurdles for bench-to-bedside translation. Indeed, for instance, fluorescence imaging is being more an experimental approach and requires the development of new technologies before adoption into the clinic. Indeed, despite the *in vivo* promising results, optical imaging is usually tested in mouse xenograft models where the tumors are usually located superficially and therefore easily detectable. Moreover, fluorescent molecular imaging is affected by the biological tissue autofluorescence, photobleaching of fluorescent dyes, and significant spectra overlap between broadband molecules 311-313. In general, the issue could be more easily overcome by the design of multi-modal nanoplatform integrating both a new optical modality and an already proven clinical methodology to extend its utility, aimed at improving and assisting a standard methodology rather than disrupt the common clinical workflow. One more point to consider is the fact that nanomedicine development has allowed rethinking the concept of nanotheranostics, expanding its notion beyond the classical meaning of physical entity that affords both diagnostic and therapeutic functions to a broad generic nano-enabled approach. In this view, a new theranostic concept is exploiting the molecular imaging functionalities not only for diagnosis but also to aid or guide a nanotherapeutic procedure 9. It is therefore noteworthy that in some cases fluorescence can be used as optical imaging to aid or guide minimally invasive surgery, such as in the case of brain tumors 314 or for the detection of sentinel lymph nodes 315.

Concerning other imaging techniques, while radiolabeling-based nuclear imaging has been explored for 2DMs thanks to superior sensitivity and the possibility to obtain whole-body images compared to fluorescent labeling, there is still a lack of studies investigating nanomaterials for ultrasonography, an extreme safe imaging modality present in the every-day clinical practice 316.

The cardinal feature that renders 2DMs more suitable compared to other particles is undoubtedly their multiple imaging advantages, already proven by the analysis of the scientific literature 108,317. The multiple imaging should, however, take into consideration the real cancer needs, the real translation into the clinic when it will come to the diagnosis of tumors in human patients, and the potential differences among mice and humans in terms of depth of tissues and, therefore, also in terms of sensitivity. Finally, for imaging as well as for the drug delivery aims, it is vital to analyze and, possibly, quantify the material distribution in each type of theranostic study, taking into account the different cancer models, since the distribution may vary depending also on the cancer tissues.

### 6.2. Necessary considerations *before* the design of nanomaterial theranostics

The first and most crucial aspect still requiring to be fully elucidated to develop and design theranostic tools, allowing their translational application into the clinic, are the assessment of their specific toxicity. In this view, their toxicological effects and the underlying mechanisms after entering the organism (both in cancer and healthy model at the same time and with the same material) need further investigation, in particular following intravenous injection, representing the main route of administration of nanomaterials in nanotheranostics 318.

The understanding of the larger picture depicting the relationship between the structure and the activity of the materials, from living systems to the molecular level, can be ensured through new systems biology approaches 319, designing robust and validated experimental methods 320.

The relationship mentioned above can be extended to the application level, designing the structure to modulate different physicochemical properties and potential biological impacts suitable for specific imaging and therapeutic procedures in consideration of the specific tissue and form of cancer taken into consideration. This correlation is not straightforward, and its clarification requires a multi-interdisciplinary approach, involving clinicians and scientists whose efforts rely on the field of material science, chemistry, physics, biology, and toxicology. However, it must be considered that the toxicological profile of nanomaterials presents Janus's double-face in the cancer fight. On the one hand, the potential systemic toxicity may cause harm. On the other hand, localized toxic effects can be useful for clinically controlled cancer ablation, when the mechanism of action is known.

The investigation of the toxicity should not overlook their impact on the immune system, since nanomaterials directly interact with the blood immune cells when entering the bloodstream during a medical procedure. In this view, several studies and different reviews have been reported to evaluate this interaction 280,321-328. Indeed, the immune system governs every aspect of our health and plays a crucial role in the response of the organism to tumor eradication as well as to cancer therapy. In this view, immunotherapy is able to trigger the immune system of cancer patients to elicit a strong antitumor response. In view of their multifunctional properties, new 2DMs can serve as nanotheranostic tools for cancer immunotherapy thanks to their ability to modulate the immune system to eradicate cancer. Moreover, their functionalization with specific monoclonal antibodies, such as Rituxan, allows to specifically recognizing the cancer cells leading to tumor destruction 49. The potentialities of 2DMs as immunotherapeutics need further investigations as done for other types of nanomaterials 329.

So far, *in vivo* oncological nanotheranostic research has been performed primarily in mice models, which are often not sufficiently adequate clinically relevant models. In these models, the tumor usually derives from immortalized human tumor cell lines after subcutaneous injection, which shows a variable success rate of cancer xenograft growth and not well represent the patient tumor heterogeneity offered by patient-derived xenograft models or accurate genetically engineered mouse models. To accelerate the advance to clinical implementation, adequate supportive data related at least to nanotool performance in terms of imaging, along with long-term assessment of their safety, stability and biodistribution, need to be acquired in larger animal models different from mice, which share more physiologic similarities with humans and present a longer life span, such as swine and non-human primates. In particular, these translational (preclinical) models (to human clinical trials) would provide a suitable step to evaluate the long-term destination of theranostic nanotools in the body, which is particularly relevant for slowly degradable or nondegradable nanomaterials, to assess whether and to what extent they can persist and accumulate in the organism for a long time, eventually leading to chronic inflammation. The possibility to control these aspects would allow mitigating the side effects and therefore improving the therapeutic efficacy.

As a critical step for the evaluation of their biocompatibility, the investigation of the *in vivo* impact of 2DMs applied to clinical application requires further toxicity and biodistribution analysis as well as metabolism studies that should be carried out applying the principles of *reduction*, *refinement*, and *replacement*. This long trial needs to be accomplished according to specific standardized procedures at an international level, such as the regulatory European guidelines EN ISO 10993.

Among these tests, the evaluation of irritation and sensitization reactions would be critical to determine the allergic response and the localized inflammatory response after, for instance, skin contact. This aspect is particularly relevant for topical application of biomedical nanotool, like PSs applied to the skin or nanosystems for wound healing 330. Moreover, beside the assessment of systemic toxicity following a single or multiple dose during a short period (acute toxicity), it is crucial to evaluate the impact of repeated doses for a prolonged period (subacute, subchronic, and chronic systemic toxicity), since the latter represents the most common administration of 2DM-based medical devices. Mutagenicity, carcinogenicity, reproductive and development toxicity also need to be investigated, especially for materials destined to have a long-term exposure to the human body. In case of materials applied for therapy, such as in the case of cancer theranostics, it would be important to investigate the biocompatibility in healthy tissues by using experimental model developed to mimic pathological conditions of the surrounded organs/tissues, since the outcome of the nano-therapy will be influenced by the morbid condition 331.

The development of scalable nanotools is also a relevant need for their clinical translation. There are still critical issues related to the large-scale production of graphene, GRMs, graphene hybrids, and, even more, for that of the other 2DMs here discussed. These limits are mainly represented by the difficulties in obtaining a large number of high-quality products, dispersible in physiological solutions and at high concentrations, in a simple, low-budget, biocompatible, sterile and green-environmentally friendly manner. Indeed, for any nanomaterial toxicological evaluation and the subsequent medical application, there is a requirement to avoid chemical and biological impurities (e.g., endotoxin contamination) 332,333, which can derive from residual reagents used for their synthesis and the lack of sterile conditions during their production, respectively. Therefore, in studies concerning the investigation of theranostic tools, including the new 2DMs here presented, these aspects must be taken into consideration. Indeed, all the above-mentioned requirements are not easy and obvious to obtain for all the materials here analyzed for cancer theranostics.

In addition, defects and variability in size and thickness that can occur during production may affect the performance and the theranostic effectiveness of the material. It is crucial to deeply characterize the materials through well-established and standardized techniques to evaluate the impact of their properties (e.g., lateral size, number of layers, shape, surface charge, elasticity, chemical modification, etc.) 334. Indeed, it has been highlighted the importance of considering the family of GRMs as composed by different individual materials with specific physicochemical characteristics, which in turn will affect their toxicity 253, and the same concept should be applied for their theranostic potentialities.

Moreover, a rational, systematic classification, allowing a scientific comparison among the different materials and their specific characteristics, is required to optimize and predict their possible theranostic applications. The current Food and Drug Administration (FDA) process to approve nanodrugs does not differ from that of any other drug or biologic 16,335-337, involving three phases of clinical trials, but a specific regulation of nanotechnology medical products is still missing and expected to be released 336. To this end, it is essential to fully elucidate the biocompatibility of nanomaterials to draw conclusions on their toxicity and possible hazards in order to develop proper guidelines.

Altogether, these observations show a puzzling picture where many aspects still need to be further explored or improved. However, the growing research in the field highlights how the scientific community is becoming aware that cooperation and multi- and inter-disciplinary approaches are essential to address the abovementioned limits in designing cancer theranostics. We hope that our conclusions will help the community to reflect on the key considerations and open questions for 2D theranostics development. Overall, it is clear that the applications of graphene, graphene hybrids, and other novel 2DMs for cancer theranostics have the potentialities to offer a substantial contribution in the fight against cancer.

### 6.3. The future of 2D materials: clinical translation

2D materials represent a thriving field not least in cancer theranostics, due to the manifold advantages of these materials including their varied physicochemical properties, and low-cost production. The challenge of translating graphene and other 2DMs into the clinic is not a new undertaking. Similar concerns exist in every cutting-edge technology in the biomedical field, such as gene therapy, genome editing or stem cell therapeutics. In recent years, we have witnessed an exceptional accumulation of new knowledge on graphene and its applications in medicine. In terms of nanotechnologies applied to biology, graphene is represented by two different forms: as an engineered graphene-incorporating device, for use as sensors and implants, or as a highly oxygenated and structurally defective form of graphene oxide and its derivatives, stable in water and easy to functionalize. The latter formulations are currently explored in different drug delivery, photothermal, photodynamic, pharmacological, toxicological, and theranostical studies. The growing interest in the translation of graphene and other 2DMs into a range of application areas, including medicine, promoted, in part, by the Graphene Flagship project 2, is thus fueling great expectations. Graphene has been tested *in vitro* and *in vivo* obtaining promising results, which now allows us to look at the next phase of clinical studies and the safe translation of these materials into the clinic in the coming decades ahead.

Considering the fact that graphene has undergone such an incubation process over several years 338, in order to make this class of materials mature for the clinical translation, it is necessary to take a step back in order to ascertain the impact of other emerging 2DMs on human health and find the particular role that each material may play. Considering the entire panorama of 2DMs and taking into account their different properties it is impossible to say that a single type of material will be able to perform every type of biomedical applications; it is clearly more reasonable to select, optimize, and further develop each material for a specific purpose in order to provide solutions for unmet clinical needs.

## Figures and Tables

**Figure 1:**
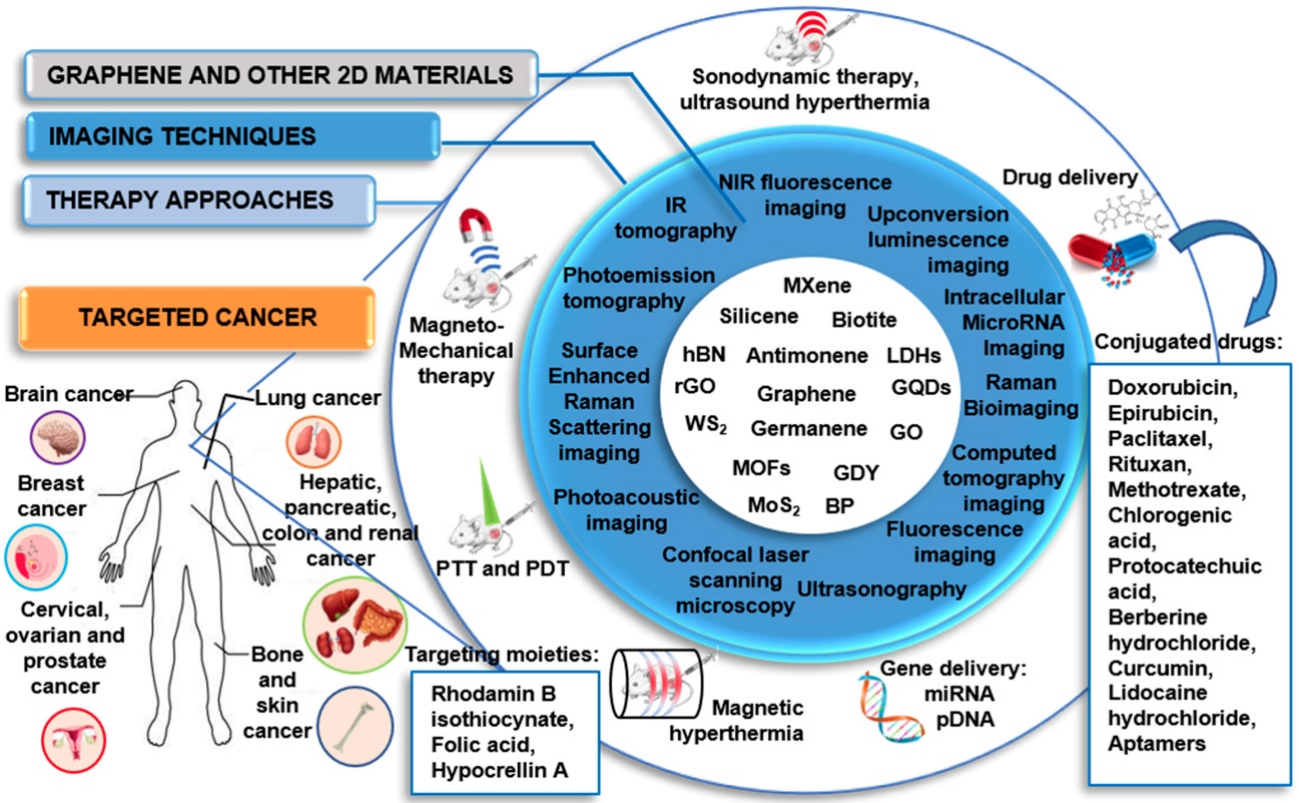
Graphical representation of the theranostic applications of different GRMs and 2DMs beyond graphene.

**Table 1:** Characterization of all the studies using GRMs in cancer theranostics *for combined imaging and drug delivery*, on the basis of type of imaging, therapy, cancer, cell line, model, drug, gene, targeting moieties & other molecules, material and name of the nanotools.

	GRAPHENE-RELATED MATERIALS										
	Application		Target			Functionalization/coating			Graphene-related material		Reference
	Imaging	Therapy	Cancer	Cell line	Model	Drug	Gene	Targeting moieties & other molecules	Material	Name	
**Imaging and drug/gene delivery**	MRI, CLSM	Drug delivery	Breast cancer	4T1	*In vitro, in vivo*	DOX	Suitable for gene delivery	SPION	GO	CAD-SPIONs@GO	Luo Y.* et al*. Chem Comm (2019)
	CLSM	Drug delivery	Breast cancer	MDA-MB 231	* In vitro*	DOX, FA	-	-	rGO	FA-rGO/ZnS:Mn QDs	Diaz-Diestra D.* et al*. Nanomaterials (2018)
	MRI	Drug delivery	Liver cancer	HepG2	* In vitro*	CA	-	Gd, Au	GO	BIT	Usman M.S.* et al*. PLoS ONE (2018)
	MRI	Drug delivery	Liver cancer	HepG2	*In vitro*	PA	-	Gd, Au	GO	GAGPAu (or GOTs)	Usman M.S.* et al*. Molecules (2018)
	CLSM	Drug delivery	Breast cancer	BT-474, MCF-7	*In vitro*	DOX	-	HER and beta-cyclodextrin	GQDs	GQD-NH_2_, GQD-βCD, and GQD-comp, DL-GQD	Ko N.R.* et al*. RSC Adv.(2017)
	MRI, CLSM	Drug delivery	Cervical cancer	HeLa	*In vitro*	DOX	-	-	GQDs	Fe_3_O_4_@SiO_2_@GQD-FA/DOX	Su X.* et al* Biosensors and Bioelectronics (2017)
	CLSM, fluorescence imaging	Drug delivery	Breast cancer	4T1	*In vitro, in vivo*	DOX	-	Cy	GQDs	DOX@GQD-P-Cy	Ding H.* et al*. ACS Appl Mater Interfaces (2017)
	Fluorescence microscopy, flow cytometry	Drug delivery	Breast cancer	MDA-MB-231, MCF-7	*In vitro*	Apt	-	-	GO	MUC1 aptamer-NAS-24 aptamer-GO, MUC1 aptamer-Cytochrome C aptamer-GO	Bahreyni A.* et al*. Int J Pharm (2017)
	MRI	Drug delivery	Renal cancer	786-0	*In vitro, in vivo*	Apt and DOX	-	Gd2O3, BSA	GO	GO/BSA-Gd_2_O_3_/AS1411-DOX	Li J.* et al*. J Biomed Nanotechnol. (2016)
	Fluorescence imaging	Drug delivery	Bone cancer	MG-63	*In vitro, in vivo*	PTX	-	ICG	NGO	NGO-PEG-ICG/PTX	Zhang C.* et al*. RSC Adv. (2016)
	CLSM, flow cytometry	Drug delivery	Breast, cervical cancer	HeLa and MDA-MB-231	*In vitro*	BHC	-	-	GQDs	GQDs@Cys-BHC	Thakur M.* et al*. Mater Sci Eng C Mater Biol Appl. (2016)
	CLSM	Drug delivery	Lung, cervical, breast, liver cancer	A549, HeLa, MCF-7, HepG-2	*In vitro*	Apt	-	-	GQDs	AS1411-GQDs	Wang X.* et al*. J. Mater. Chem. B (2015)
	Intracellular microRNA imaging	Gene deliery	Cervical cancer	HeLa	*In vitro*	-	miRNAs-21	-	GQDs	f-GQDs	Dong H.* et al* ACS Appl Mater Interfaces. (2015)
	MRI, CLSM	Drug and gene delivery	Brain cancer	U87	*In vitro, in vivo, ex vivo*	EPI	Let-7g miRNA.	-	NGO	Gd-NGO/Let-7g/EPI	Yang H.W.* et al*. Biomaterials. (2014)
	Optical imaging	Drug delivery	Liver cancer	Bel-7402, SMMC-7721, HepG2	*In vitro*	DOX	-	-	GO	GO-RGD-Chitosan	Wang C.* et al* Colloids and Surfaces B: Biointerfaces (2014)
	Fluorescence imaging PAI	Drug delivery	Lung cancer	H1975	*In vivo*	DOX	-	Cy5.5	GO	GO-Cy5.5-Dox	Nie L.* et al*. ACS Nano (2014)
	Fluorescence microscopy	Drug delivery	Colon cancer	HCT116	*In vitro, in vivo*	Cur	-	-	GQDs	GQDs-Cur	Some S.* et al.* Scientific Reports (2014)
	CLSM	Drug delivery	Cervical cancer	HeLa and L02	*In vitro*	DOX	-	Lysotracker Green	GO	DOX@MSP-BA-GOF	He D.* et al* Langmuir (2014)
	Fluorescence imaging	Drug delivery	Cervical cancer	HeLa	*In vitro*	DOX	-	-	graphene-HQDs	DOX-graphene-HQDs-Trf	Chen M.L.* et al.* Bioconjugate Chem. (2013)
	CLSM, Raman imaging	Drug delivery	Cervical cancer	HeLa	*In vitro*	DOX	-	Au	GO	Au@NGO	Ma X.* et al*. J. Mater. Chem. B, 2013
	MRI, CT imaging	Drug delivery	Cancer	-	Not tested in biological models	-	-	Au/Fe_3_O_4_andBaTiO_3_/Fe_3_O_4_	rGO	rGO/Au/Fe_3_O_,_ andrGO/BaTiO_3_/Fe_3_O_4_	Chen Y.* et al.* ACS Nano. (2013)
	MRI	Drug delivery	Liver cancer	HepG2	*In vitro*	DOX	-	Gd(III)	GO	GO-DTPA-Gd	Zhang M.* et al.* ACS Appl. Mater. Interfaces (2013)
	MRI	Drug delivery	Brain cancer	U251	*In vitro*	DOX	-	MGMSPID	GO	MGMSPI	Wang Y.* et al* Small (2013)
	Fluorescence imaging	Drug and gene delivery	Lung, prostate cancer	A549, LLC1, PC3, C42b	*In vitro, in vivo*	DOX	pDNA	-	GO	DOX-CMG-GFP-DNA	Wang C.* et al*. J Mater Chem B Mater Biol Med. (2013)
	PET	Drug delivery	Breast cancer	MCF-7	*In vitro, in vivo, ex vivo*	TRC105	-	^64^Cu	rGO	^64^Cu-NOTA-rGO-TRC105	Shi S.* et al.* Biomaterials (2013)
	MRI, fluorescence imaging	Drug delivery	Liver cancer	HepG2	*In vitro*	DOX	-	SiO_2_	GO	GO-SiO_2_	Gao Y.* et al.* Colloids and Surf. B Biointerfaces (2013)
	PET	Drug delivery	Breast cancer	MCF-7, 4T1	*In vitro, in vivo*	TRC105	-	^64^Cu	GO	^64^Cu-NOTA-GO-TRC105	Hong H.* et al.* ACS Nano (2012)
	Fluorescence imaging	Gene delivery	Cervical, prostate Cancer	HeLa and PC-3	*In vitro*	-	pDNA (pCMV-Luc)	BPEI	GO	GO-BPEI	Kim H.* et al.* Bioconjugate Chem. (2011)
	Fluorescence imaging, NIR imaging	Drug delivery	Burkitt's Lymphoma	Raji B-cell	*In vitro*	DOX, Rituxan	-	-	NGO	NGO-PEG/DOX + Rituxan	Sun X.* et al*. Nano Res. (2008)

**Table 2:** Characterization of all the studies using GRMs in theranostics *for combined imaging, PTT and* PDT, on the basis of type of imaging, therapy, cancer, cell line, model, PS, targeting moieties, material and name of the nanotools.

	GRAPHENE-RELATED MATERIALS									
	Application		Target			Functionalization/coating		Graphene-related material		Reference
	Imaging	Therapy	Cancer	Cell line	Model	PS	Targeting moieties	Material	Name	
**Imaging and PDT/PTT**	MRI, CLSM, UCL, CT, PAT, UCLM	PTT, PDT	Cervical cancer	Hela, U14	*In vitro, in vivo*	-	-	GO	GO/ZnFe_2_O_4_/UCNPs (GZUC)	Bi H.* et al*. ACS publications (2018)
	UCL imaging	PTT, PDT	Cervicalliver cancer	HeLa, U14	*In vitro, in vivo, ex vivo*	-	Ce6	NGO	NGO-UCNP-Ce6 (NUC)	Gulzar A.* et al*. Dalton Trans (2018)
	X-ray CT imaging, PAI	PTT	Cervical cancer	HeLa	*In vitro, in vivo*	-	-	GO	GO/Bi_2_Se_3_/PVP	Zhang Y.* et al*. J. Mater. Chem. B. (2017)
	NIR imaging, PAT	PTT	Skin cancer	SCC7	*In vitro, in vivo*	Au	Cy5.5	GO	CPGA	Gao S.* et al* Biomaterials (2016)
	Fluorescence imaging	PTT, PDT	Skin cancer	B16F0	*In vivo*	-	-	NGO	GO-PEG-folate-mediated NmPDT	Kalluru P.* et al*. Biomaterials (2016)
	PAI	PTT	Brain cancer	U87MG	*In vitro, in vivo*	-	-	rGO	PEG-rGO-GSPs	Lin L.S.* et al*. Nanoscale. (2016)
	CLSM, NIR fluorescence and thermal imaging	PTT, PDT	Lung cancer	A549 and Lewis lung cancer cells	*In vitro, in vivo*	-	-	NGO	NGO-808	Luo S.* et al*. ACS Appl Mater Interfaces. (2016)
	PAI	PTT	Breast cancer	4T1	*In vitro, in vivo*	-	ICG	GO	ICG-PDA-rGO	Hu D.* et al* Theranostics. (2016)
	MRI, fluorescence imaging, IR thomography	PTT	Sarcoma	S180	*In vitro, in vivo*	IO	-	GO	IO/GO-COOH	Huang G.* et al* Nanoscale (2015)
	Ramanbioimaging	PTT, PDT	Cervical cancer	HeLa	*In vitro*		-	GO	PEG-Au@GON	Kim Y.K.* et al*. Small. (2015)
	PAI	PTT	Breast cancer	4T1	*In vitro, in vivo*	-	-	GO	GO-PEG-CysCOOH	Rong P.* et al*. RSC Adv. (2015)
	Fluorescence imaging, PAI	PTT, PDT	Lung cancer	PC9	*In vitro, in vivo*	-	DVDMS	GO	GO-PEG-DVDMS	Yan X.* et al*. Nanoscale. (2015)
	MRI, CT imaging	PTT	Cervical cancer	HeLa	*In vitro, in vivo*	Gd(III)	-	GO	GO/BaGdF5/PEG	Zhang H.* et al*. Biomaterials. (2015)
	Fluorescence imaging	PDT	Cervical Cancer	HeLa, MDA MB-231	*In vitro*	-	-	GQDs	NGs-QDs	Ge J.* et al* Nature Communications (2014)
	MRI, fluorescence imaging	PTT, PDT	Cervical Cancer	HeLa	*In vitro*	SiNc_4_	-	MGF	MFG	Gollavelli G.* et al* Biomaterials (2014)
	Fluorescence imaging	PTT, PDT	Skin cancer	G361	*In vitro*	-	ICG	GO	ICG-FeCl_3_ @GO	Viraka Nellore* et al*. Faraday Discuss. (2014)
	Raman imaging	PTT	Breast Cancer	SKBR-3	*In vitro*	-	-	GO	GO and GOAuNS	Nergiz S.Z.* et al* ACS Appl. Mater. Interfaces (2014)
	CLSM, flow citometry, molecular imaging	PTT, PDT	Breast cancer	MDA-MB231	*In vitro, in vivo*	-	-	cGdots	cGdots	Nurunnabi M.* et al*. ACS Appl. Mater. Interfaces (2014)
	MRI	PTT	Pancreatic cancer	BxPC-3	*In vitro, in vivo*	ION	-	GO	GO-ION-PEG	Wang S.* et al* Biomaterials (2014)
	Fluorescence imaging, PET	PTT, PDT	Breast cancer	4T1	*In vitro, in vivo, ex vivo*	-	HPPH	GO	GO-PEG-HPPH	Rong P.* et al*. ADV Theranostics (2014)
	NIR fluorescence imaging	PTT, PDT	Lung cancer	A549	*In vitro*	-	Ce6	GO	GO-HA-Ce6	Cho Y.* et al*. Chem Commun Camb (2013)
	MRI, CLSM	PTT, PDT	Cervical cancer	KB, HeLa	*In vitro, in vivo*	-	-	NGO	UCNPs-NGO/ZnPc	Wang Y.* et al*. Biomaterials. (2013)
	MRI, X-ray CT	PTT	Cervical, breast cancer	KB, 4T1	*In vitro, in vivo*	IONP-Au	-	GO	GO-IONP-Au-PEG	Shi X.* et al*. Biomaterials. (2013)
	PAI	PTT	Cervical cancer	Hela	*In vitro*	-	ICG	GO	ICG-GO-FA	Wang Y.W.* et al*. Journal of material chemistry B (2013)
	PAI	PTT	Breast cancer	MCF-7	*In vitro, in vivo*	-	-	NrGO	NrGO	Sheng Z.* et al* Biomaterials (2013)
	MRI, fluorescence imaging, PAI	PTT	Breast cancer	4T1	*In vivo*	IONP	-	rGO	rGO-IONP-PEG	Yang K.* et al* Advanced Materials (2012)
	Fluorescence imaging	PTT	Breast, cervical cancer	HeLa, MCF-7	*In vitro, in vivo*	-	-	rGO	rGO-QD	Hu S.H.* et al* Advanced Materials (2012)
	Fluorescence imaging	PTT	Breast cancer	4T1	*In vivo*	-	-	NGS	NGS-PEG	Yang K.* et al* Nanoletters (2010)

**Table 3:** Characterization of all the studies using GRMs in theranostics *for combined imaging, drug delivery, PTT and* PDT, on the basis of type of imaging, therapy, cancer, cell line, model, drug, gene, PS, targeting moieties, material and name of the nanotools.

	GRAPHENE-RELATED MATERIALS											
	Application		Target			Functionalization/coating				Graphene-related material		Reference
	Imaging	Therapy	Cancer	Cell line	Model	Drug	Gene	PS	Targeting moieties	Material	Name	
**Imaging, drug delivery and PTT/PDT**	MRI	Drug delivery, PTT	Breast cancer	4T1	*In vitro*	MTX	-	Mn(II)	DTPA	rGO	rGO-PDA-BSA-DTPA Mn(II)/MTX	Karimi Shervedani R.* et al*. Biosens Bioelectron. (2018)
	MRI, PAI	Drug delivery. PTT	Breast cancer	4T1	*In vitro, in vivo*	DOX	-	MnWO4	-	GO	GO/MnWO_4_/PEG	Chang X.* et al*. Carbon (2018)
	CLSM, UCL imaging	Drug delivery, PTT	Cervical, liver cancer	Hela, U14	*In vitro, in vivo*	DOX	-	-	FITC	NGO	UCNPs-DPA-NGO-PEG-BPEI-DOX	Gulzar A.* et al*. Dalton Trans (2018)
	CLSM	Gene delivery, PTT, PDT	Lung, breast cancer	A549, MCF-7	*In vitro*	-	miRNA	-	-	GQDs	GQD-PEG-P	Cao Y.* et al*. ACS Appl. Mater. Interfaces (2017)
	CLSM, thermal/PT imaging	Drug delivery, PTT, PDT, sonodynamic therapy	Breast cancer	EMT6	*In vitro, in vivo*	-	-	-	Ce6	GO	GO/AuNS-PEG and GO/AuNS-PEG/Ce6	Wu C.* et al*. Acta Biomater. (2017)
	CLSM, NIR fluorescence imaging	Drug delivery PTT, PTT	Breast cancer	B16F10, MCF-7	*In vitro, in vivo*	-	-	-	PheoA	GO	PheoA + GO:FA-BSA-c-PheoA NC	Battogtokh G.* et al*. Journal of Controlled Release (2016)
	CLSM	Drug delivery, PTT	Cervical cancer	HeLa	*In vitro*	LH	-	-	-	rGO, GQDs	MGQDs-LH	Justin R.* et al*. Carbon (2016)
	Fluorescence imaging, CLSM, SERS imaging, Optical imaging, Raman imaging	Drug delivery, PTT	Lung cancer	A549	*In vitro*	anti‐EGFR SERS probes	-	-	-	rGO	anti‐EGFR‐PEG‐rGO@CPSS‐Au‐R6G	Chen Y.W.* et al*. Small (2016)
	NIR imaging, SERS	Drug delivery, PTT	Breast cancer	4T1	*In vitro, in vivo*	DOX	-	-	PANI	GO	GO-Au@PANI/DOX	Chen H.* et al.* Theranostics (2016)
	MRI	Drug delivery, PTT	Breast cancer	MCF-7	*In vitro, in vivo*	DOX	-	Gd(III)	-	GO	GO@Gd-PEG-FA/DOX	Shi J.* et al*. Pharm Res. (2016)
	Molecular imaging, real-time IR thermal imaging	Drug delivery, PTT	Cervical cancer	KB	*In vitro, in vivo*	-	-	-	ICG	rGO	ICG/HArGO and ICG/rGO	Miao W.* et al*. J Control Release. (2015)
	Fluorescence imaging	Drug delivery, PTT, PDT	Ovarian cancer	A2780/AD	*In vitro, in vivo*	-	-	-	Pc	LOGr	LOGr-Pc-LHRH	Taratula O.* et al*. Int. J. Nanomed (2015)
	Fluorescence imaging	Drug delivery, PDT	Brain cancer	U87MG	*In vitro, in vivo, ex vivo*	-	-	-	DVDMS	GO	GO-PEG-DVDMS	Yan X.* et al*. Biomaterials. (2015)
	Fluorescence imaging, MRI	Drug delivery, PTT	Cervical cancer	Hela	*In vitro*	DOX	-	-	-	rGO	DOX-rGO-Fe_2_O_3_@Au NPs	Chen H.* et al*. RCS Adv. (2015)
	Fluorescence imaging	Drug delivery, PDT	Cervical cancer	HeLa	*In vitro*	HA	-	-		GQDs	HA-GQD-SiO_2_	Zhou L.* et al*. Chem. Commun. (2015)
	Photothermal imaging, optical imaging	Drug delivery, PTT	Lung cancer	A549	*In vitro, in vivo*	DOX	-	-	-	Graphene	GDH	Khatun Z.* et al*. Nanoscale (2015)
	MRI	Drug delivery, PTT	Cervical cancer	HeLa	*In vitro, in vivo, ex vivo*	DOX	-	MnFe_2_O_4_	-	GO	GO/MnFe_2_O_4_/DOX	Yang Y.* et al*. Journal of Biomaterials Applications (2015)
	TPL	Drug delivery, PTT	Breast cancer	MCF-7	*In vitro*	DOX	-	Au	-	graphene	NGsAu nanocrystal	Bian X.* et al* Scientific Reports (2014)
	CLSM	Gene delivery, PTT	Breast cancer	MCF-7	*In vitro*	DOX	-	-	FITC and DAPI	NGO	GO-PEG-DA	Feng L.* et al* Adv Healthc Mater. (2014)
	CLSM	Drug delivery PTT	Breast cancer	MCF-7	*In vitro, in vivo*	DOX	-	Ag	-	GO	GO-Ag	Shi J.* et al* Biomaterials (2014)
	Fluorescence imaging, X-ray CT imaging, US imaging	Drug delivery, PTT	Cervical cancer	Hela	*In vitro, in vivo*	PLA	-	-	-	GO	Au@PLA-(PAH/GO)n	Jin Y.* et al*. Biomaterials (2013)
	CLSM	Drug delivery, PTT	Brain cancer	U251	*In vitro*	DOX	-	-	-	Graphene	GSPI	Wang Y.* et al* J.Am.Chem.Soc. (2013)
	UCL imaging	Drug delivery, PTT, PDT	Breast cancer	KB, HeLa	*In vitro, in vivo, ex vivo*	ZnPc	-	-	ZnPc	GO	GO-UCNPs--ZnPc	Wang Y.* et al* Biomaterials (2013)
	Molecular imaging	Drug delivery, PTT	Skin cancer	SCC7	*In vitro, in vivo*	DOX	-	-	Ce6	GO	Ce6/Dox/pGO	Miao W.* et al* Biomaterials (2013)

**Table 4:** Characterization of all the studies using 2DMs in cancer theranostics, on the basis of type of imaging, therapy, cancer type, cell line, model, drug, PS, targeting moieties, material, and name of the 2D tools.

	NEW 2D MATERIALS								
	Applications		Target			Functionalization/coating		2D materials	Reference
	Imaging	Therapy	Cancer	Cell line	Model	Drug	Other molecules/ particles		
WS_2_	PAI, MRI, fluorescence imaging	PTT and radiotherapy	Breast cancer	4T1	*In vitro, in vivo*	-	IONPs, MnO_2_	WS_2_-IO/S@MO-PEG	Yang G.* et al*.Small (2018)
	SPECT, IR thermal andfluorescent imaging	PTT and radiotherapy	Breast cancer	4T1	*In vitro, in vivo*	-	^188^Re	^188^Re-WS_2_-PEG	Chao Y.* et al*.Small (Weinheim an der Bergstrasse, Germany, 2016)
	CT, IR and fluorescence imaging	PTT, PDT	Cervical cancer	HeLa	*In vitro, in vivo*	-	Bovine serum albumin (BSA), methylene blue	BSA-WS_2_@MB	Yong Y.* et al*.Nanoscale (2014)
	PAT, CT, IR imaging	PTT	Cervical and breast cancer	4T1, HeLa and 293T	*In vitro, in vivo*	-	LA-PEG	WS_2_-PEG	Cheng L.* et al*.Advanced Materials (2014)
MoS_2_	MRI, CLSM, flow cytometry	Drug delivery, PTT	Lung cancer	A549, H1975	*In vitro, in vivo*	Gefitinib	Hyaluronic acid (HA), gadolinium (Gd), DTPA	MoS_2_-HA-DTPA-Gd	Liu J.* et al*.Journal of Colloid and Interface Science (2019)
	NIR fluorescence imaging	Drug delivery, PTT	Liver cancer	LO2, Hep3B	*In vitro*	MET	Mn-doped Fe_3_O_4,_ chitosan	Mn-doped Fe_3_O_4_@MoS_2_@CS	Jing X.* et al*. Bioconjugate Chemistry (2018)
	Two-photon CLSM and fluorescence imaging	PTT, PDT	Cervical cancer	HeLa	*In vitro*	-	AuNBPs	AuNBPs@MoS_2_	Maji S.* et al*. ACS Applied Materials and Interfaces (2018)
	IR, PET, FLIM, Flow cytometry	Drug delivery, PTT	Breast and lung cancer	A549, MCF-7, MCF-7-ADR	*In vitro, in vivo*	DOX	PEI, HA	MoS_2_-PEI-HA	Dong X.* et al*. ACS Applied Materials and Interfaces (2018)
	MRI, PAI, CLSM	PTT	Breast cancer	4T1, RAW 264.7, L929	*In vitro, in vivo*	-	Bovine serum albumin-gadolinium (BSA-Gd)	MoS_2_-Gd-BSA	Chen L.* et al*. ACS Applied Materials and Interfaces (2017)
	MR, IR, and PA imaging	PTT, PDT and chemotherapy	Hepatoma and cervical cancer	L929	*In vitro, in vivo*			MoS_2_@Fe_3_O_4_-ICG/Pt(IV) Nanoflowers	Liu B.* et al*. Advanced Science (2017)
	MRI, CT, CLSM, UCLM	PTT, PDT	Cervical cancer	HeLa, L929	*In vitro, in vivo*	-	chlorin e6 (Ce6), UCS	MUCS-FA	Xu J.* et al*. Small (2017)
	PAI, NIR	PTT	Colorectal cancer	HT29, L929	*In vitro, in vivo*	-	PVP	MoS_2_-PVP	Zhao J.* et al*. Oncotarget (2017)
	IR and phase contrast imaging	PTT	Breast cancer	4T1, L929	*In vitro, in vivo*	-	Soybean phospholipid	SP-MoS_2_	Li X.* et al*. International Journal of Nanomedicine (2016)
	MRI, PAT and fluorescence imaging	PTT	Cervical and liver cancer	HeLa, HepG2	*In vitro*	-	Fe_3_O_4_, PEG	MSIOs	Yu J.* et al*. Theranostics (2015)
	PAI, CT, IR	PTT and radiotherapy	Breast cancer	4T1	*In vivo*	-	Bi_2_S_3_	MoS_2_/Bi_2_S_3_	Wang S.* et al*. Advanced Materials (2015)
	PAT, MRI, IR, PET	PTT	Breast cancer	4T1, RAW 264.7	*In vitro, in vivo*	-	LA-PEG, IONPs, ^64^Cu	^64^Cu-MoS_2_-IO-(d)PEG	Liu T.* et al*. ACS Nano (2015)
	CLSM, IR, flow cytometry	PTT, PDT	Breast cancer	4T1	*In vitro, in vivo*	-	Ce6, LA-PEG	MoS_2_-PEG	Liu T.* et al*. Nanoscale (2014)
BP	PAI, fluorescence imaging	PTT	Breast and lung cancer	A549, MCF-7, LO2	*In vitro, in vivo*	-	RGD	RP-p-BPNSs	Li Z.* et al*. ACS Applied Materials and Interfaces (2019)
	MR, IR, CLSM	PTT, PDT	Cervical cancer	HeLa	*In vitro, in vivo*	-	Fe_3_O_4_-CDs, GP, PGA	GP-PGA-Fe_3_O_4_-CDs@BPQDs	Zhang M.* et al*. International Journal of Nanomedicine (2018)
	MRI, fluorescence imaging, flow cytometry	PDT and radiotherapy	Melanoma	A375	*In vitro, in vivo*	-	Bi_2_O_3_	BP/Bi_2_O_3_	Huang H.* et al*. Biomaterials (2018)
	IR thermal, CLSM	PTT	Cervical cancer	HeLa	*In vitro, in vivo*	-	-	BPQDs	Wang M.* et al*. Analyst (2018)
	MRI, IR, CLSM	Drug delivery, PTT	Breast and lung cancer	A549, MCF-7	*In vitro, in vivo*	DOX	Fe_3_O_4_@C, SiO_2_	BPQDs@ss-Fe_3_O_4_@C	Zhang M.* et al*. Chemistry - A European Journal (2018)
	IR thermal, fluorescence imaging	Drug delivery	Breast cancer	MDA-MB-231	*In vitro, in vivo*	DOX	-	BP@Hydrogel	Qiu M.* et al*. Proceedings of the National Academy of Sciences (2018)
	IR thermal, fluorescence imaging	PTT, PDT	Cervical cancer	HeLa	*In vitro, in vivo*	-	PEG, Ce6	BP@PEG/Ce6 NSs	Yang X.* et al*. ACS Applied Materials and Interfaces (2018)
	IR thermal, CLSM	PTT	Osteogenic sarcoma	Saos-2	*In vitro, in vivo*	-	Bioglass (BG)	BP-BG scaffold	Yang B.* et al*. Advanced Materials (2018)
	IR thermal, fluorescence imaging	Drug delivery, PTT, PDT	Breast cancer	4T1	*In vitro, in vivo*	DOX	-	BP	Chen W.* et al*. Advanced Materials (2017)
	PAI	PTT, PDT	Breast cancer	MCF-7	*In vitro, in vivo*	-	TiL_4_	TiL_4_@BPQDs	Sun Z.* et al*. Small (2017)
	NIR, CLSM	Drug delivery, PTT	Cervical cancer	HeLa	*In vitro, in vivo*	DOX	PEG, Cy7	BP-PEG-FA/Cy7 NSs	Tao W.* et al*. Advanced Materials (2017)
	IR thermal, CLSM	PTT, PDT	Liver and breast cancer	HepG2, 4T1	*In vitro, in vivo*	-	RdB, PEG	RdB/PEG-BPQDs	Li Y.* et al*. ACS Applied Materials and Interfaces (2017)
	IR thermal imaging	PTT	Breast cancer	4T1	*In vitro, in vivo*	-	Au	BP-Au NSs	Yang G.* et al*. Biomaterials Science (2017)
	PAI, IR thermal, and fluorescence imaging	PTT	Breast cancer	4T1	*In vitro, in vivo*	-	PEG	PEGylated BP	Sun C.* et al*. Biomaterials (2016)
MXene	IR thermal, PAI, CLSM, fluorescence imaging, flow cytometry	PTT and photonic thermodynamic therapy	Breast cancer	4T1	*In vitro, in vivo*	-	AIPH, SiO_2_	AIPH@Nb_2_C@mSiO_2_	Xiang H.* et al*. ACS Nano (2019)
	PAI, CT, CLSM, IR thermal	PTT and radiotherapy	Breast cancer	4T1	*In vitro, in vivo*	-	Au	Ti_3_C_2_@Au	Tang W.* et al*. ACS Nano (2019)
	MRI, CT, CLSM	PTT	Breast cancer	4T1	*In vitro, in vivo*	-	Soybean phospholipid, IONP	Ta_4_C_3_-IONP-SPs	Liu Z.* et al*.Theranostics (2018)
	PAI, CLSM, IR, flow cytometry	PTT	Brain cancer	U87	*In vitro, in vivo*	-	CTAC, RGD	CTAC@Nb2C-MSN	Han X.* et al*.Theranostics (2018)
	PAI, IR, CLSM	Drug delivery, PTT	Liver cancer	HCC, SMMC-7721	*In vitro, in vivo*	DOX	RGD	Ti3C2@mMSNs	Li Z.* et al*. Advanced Materials (2018)
	PAI, fluorescence imaging	Drug delivery, PTT	Breast cancer	4T1	*In vitro, in vivo*	DOX	Soybean phospholipid	Ti_3_C_2_-SP	Han X.* et al*. Advanced Healthcare Materials (2018)
	IR thermal, PAI and fluorescence imaging	PTT	Breast and brain cancer	4T1, U87	*In vitro, in vivo*	-	PVP	Nb_2_C-PVP	Lin H.* et al*. Journal of the American Chemical Society (2017)
	IR thermal, PAI	PTT	Cervical cancer	HeLa	*In vitro and in vivo*	-	-	Ti_3_C_2_ QDs	Yu X.* et al*. Nanoscale (2017)
	MRI, CT, PAI, CLSM	PTT	Breast cancer	4T1	*In vitro and in vivo*	-	MnOx, soybean phospholipid	MnOx/Ta_4_C_3_-SP	Dai C.* et al*. ACS Nano (2017)
	CLSM, IR thermal, fluorescence	PTT, PDT	Colon cancer	HCT-116	*In vitro and in vivo*	DOX	HA	Ti_3_C_2_-DOX	Liu* et al*. AM&I (2017)

**Table 5A:** Table summarizing the different methodologies for the characterization of GRMs.

Ref	Material		Characterisation Techniques			
	GRMs	Acronym	Microscopy	Elemental Analysis/ Diffraction	Spectroscopy	Zeta Potential/ DLS/Stability/Others
Luo et al. Chem Comm (2019)	GO	CAD-SPIONs@GO	TEM: GO height= 0.5-1.1 nm & size= ~100 nm.	XPS shows partial reduction.; XRD confirmsGO	RS: confirm SPION growth on GO; FT-IR confirms functionalisation with CAD; 1H NMR confirms functionalisation of GO with CAD	Zeta: GO = -52 mV & CAD-SPIONs@GO = +201 mV; DLS: GO = 127 nm & CAD-SPIONs@GO = 175 nm; TGA confirms functionalisation
Diaz-Diestra et al. Nanomaterials (2018)	rGO	FA-rGO/ZnS:Mn QDs	TEM: GO size =200-500 nm; SEM: ZnS:Mn NPs well dispersed onto the surface of rGO.	SAED confirms ZnS:Mn; XRD: peak at 10° for GO, which disappears for rGO/ZnS:Mn	RS: confirms reduction of GO & functionalisation with QD & ZnS; UV-Vis&FTIR: confirms reduction & functionalization.	Zeta: increases from +5.5 mV to -18 mV with FA conjugation of rGO-ZnS:Mn in PBS; Stability: Stable for 24 h, in cellular media supplemented with FBS
Usman et al. PLoS ONE (2018)	GO	BIT	TEM: confirms formation of the hybrids; size= 200-500 nm	ICP‒ES & EDS: C & H % increase from GO to BIT. Gd & Au detected; XRD: peak @ 10° for GO, broadening & downshifts after functionalization	UV-Vis: confirms drug loading & release; RS(532nm): I(D)/I(G) = 0.84 for GO; =0.86 for GOGCA; = 0.94 for final BIT composite; FT-IR: confirms functionalization.	TGA: confirms the composition of the hybrid
Usman et al. Molecules.(2018)	GO	GAGPAu (or GOTs)	HRTEM: AuNPs have size of 2 nm, size of the NS (from pictures) ~500 nm	XRD: peak @ 10° for GO, broadening & downshifts after functionalization	RS(532nm): I(D)/I(G) = 0.84 for GO; 0.86 for GOGCA; 0.94 for final composite; FT-IR: confirms functionalization.	TGA: confirms the composition of the hybrid
Ko N.R. et al. RSC Adv.(2017)	GQDs	G2, GQD-βCD, & GQD-comp, DL-GQD	FE-SEM&FE-TEM: GQD size= ~20-40 nm, GQD-comp height= 6-7 nm	EDX: C, N, & O elements	UV-Vis&FT-IR: confirms surface functionalisation	DLS: GQD- NH_2_ has size of 26 nm, GQD-comp has size of 222 nm; TGA confirms the composition of the hybrid
Bahreyni et al Int J Pharm. (2017)	GO	MUC1 aptamer-NAS-24 aptamer-GO, MUC1 aptamer-Cytochrome C aptamer-GO	AFM: GO size= 195nm, slight increase after complexing		FT-IR: confirms GO	
Zhang C. et al. RSC Adv. (2016)	NGO	NGO-PEG-ICG/PTX	AFM & TEM: NGO size= 70 nm & NGO-PEG-ICG/PTX size= 95 nm. Height= ~1-2 nm		FT-IR: confirms PEGylation; UV-vis: confirms conjugation with ICG & PTX.	Zeta: -29 mV; DLS: ~100 nm, no change over time in PBS & FBS
Wang X. et al. J. Mater. Chem. B (2015)	GQDs	AS1411-GQDs	AFM: GQD size= ~30 nm & height = ~1 nm.		UV-Vis: confirms conjugation	Zeta: GQD = -21 mV
Dong et al ACS Appl Mater Interfaces.	GQDs	f-GQDs	TEM&AFM: GQD size= ~17 nm & height= ~1.3 nm, f-GQDs size = 22 nm, height = 1.7 nm; HRTEM: d-spacing of 2.5 Å	XPS: confirms GO	FT-IR: confirms functionalization	Zeta: GQD = -30 mV, GQDs-PEG = -15 mV, f-GQDs = -5 mV.
Yang HW et al. Biomaterials. (2014)	NGO	Gd-NGO/Let-7g/EPI	AFM: NGO-COOH & Gd-NGO size=~150 nm & heights= ~1.8 nm &~3 nm.	XPS: confirms oxidation & conjugation (22.8%).	UV-Vis: EPI adsorption confirmed	Zeta: Gd-NGO= +48 mV & Gd-NGO/Let/EPI= +33 mV; TGA: conjugation confirmed.
Wang C. et al Colloids Surf B Biointerfaces (2014)	GO	GO-RGD-Chitosan	AFM: RC-GO-DOX height= 2.3 nm, larger than height of GO		FT-IR& UV-Vis: confirm loading of DOX & RC; 1H NMR: confirms RGD modification of CS	Zeta: GO= -33.6 mV, RC-GO-DOX=+31.6 mV
Nie L. et al. ACS Nano (2014)	GO	GO-Cy5.5-Dox	AFM: GO-PEG size = ~10 nm, GO-PEG-Cy5.5-Dox size = ~30 nm & height= ~6 nm.		UV-Vis: confirms successful conjugation of Cy5.5 & DOX.	
Some S. et al Sci. Rep. (2014)	GO, GDO & GQDs	GO-Cur, DGO-Cur & GQD-Cur	SEM: confirms Cur loading. Cur NPs size = 120-150 nm; HRTEM: GQD size= 3-6 nm; AFM: GO & DGO size= 1-10 um.	XPS: C/O ratio= 2.2 for GO; ~1 for DGO; < 1 for GQDs	FT-IR &UV-Vis: confirm Cur functionalisation of GO, DGO & GQD	Stability: GQDs were readily water-dispersible
He D. et al (Langmuir) (2014)	GO	GO Capped Mesoporous Silica	TEM: MS- NH_2_ size= ~100 nm, capping of MS-BA by GO is confirmed.	XRD: confirms uniform mesostructure of MS- NH_2_.	FT-IR&UV-Vis: confirm BA functionalisation & PEGylation	Zeta: MS-BA-GO = -29 mV.
Chen M.L. et al (Bioconjugate Chem.) (2013)	Gr-HQDs	Gr-HQDs	TEM: Gr-PSS, Gr-HQD to show morphology of NS & uniform distribution of HQD on Gr	XPS: confirms reduction & functionalization; EDX: confirms conjugation,	UV-Vis: confirms DOX loading & GO reduction & conjugation.; RS(633nm): I(D)/I(G) = 2 for GO & 1.36 for GO-PSS.	Zeta: Gr-PSS= -38 mV; Gr-PSS-PAH-HQDs-Trf = +8.3 mV; Stability: Gr-HQDs stable in water & biological solution for 4 h at 37 °C
Ma X. et al. J. Mater. Chem. B, (2013)	GO	Au@NGO	TEM & FE-SEM: Au@NGO size <100 nm	XRD: confirms Au in the composite	RS (488 nm): confirms GO in the composite; FT-IR & UV-Vis: confirms conjugation of AuNPs	Zeta: Au@NGO = -28 mV; DLS: Au@NGO= 98 nm in water & 134 nm in DMEM.
Chen Y et al ACS Nano.(2013)	rGO	rGO/Au/Fe_3_O_4_&rGO/BaTiO_3_/Fe_3_O4	HRTEM: Fe_3_O_4_ NP covered by thin layers of rGO (~2 nm height); SEM: NP-rGO size= 300-700 nm.	XRD: rGO peak at ~18°	RS: broad D & G peaks visible, confirming the presence of GO.	Zeta: GO= -40 mV; GO/Fe_3_O_4_ system shows positive zeta for pH <7 & negative zeta for pH >7.
Zhang M. et al ACS Appl. Mater. Interfaces, (2013)	GO	GO-DTPA-Gd	AFM: GO size= 100-300 nm & height= ~1.0 nm.	XPS: confirms DTPA conjugation	FT-IR: confirms PEGylation; UV-Vis: conjugation with DOX confirmed	Zeta: GO-COOH= -47 mV, GO-PEG= -16 mV; Stability: no aggregation for 1 d in FBS & CCM
Wang Y. et al Small (2013)	GO	MGMSPI	SEM: MGMS size= ~200nm, smaller than GO; TEM: spherical NPs evenly distributed on GO; HRTEM: Fe_3_O_4_ NPs size= 4-15 nm.		RS(633nm): D, G & 2D b& confirm GO presence. I(D)/I(G)>1; FT-IR&UV-Vis: confirms conjugation & reduction.	TGA: confirms reduction of Gr when covered with MGMS
Wang C et al. J Mater Chem B Mater Biol Med. (2013)	GO	DOX-CMG-GFP-DNA	TEM: CRGO size= ~150nm		FTIR: confirms functionalisation; UV-Vis: confirms DOX loading.	DLS: CRGO= 126 nm, DOX-CMG= 91 nm
Shi S. et al Biomaterials (2013)	rGO	^64^Cu-NOTA-RGO-TRC105	SEM & AFM: size= 20- 80 nm			Zeta: RGO-PEG- NH_2_= -20 mV, NOTA-RGO-TRC105= -2 mV; DLS: RGO-PEG- NH_2_= 22 nm, NOTA-RGO-TRC105= 37 nm
Gao Y et al Colloids & Surf. B Biointerfaces (2013)	GO	GO-SiO_2_	SEM & TEM: SiO_2_ size= 70-80 nm, GO of micron-size.		FT-IR&UV-vis: confirm conjugation of GO & SiO_2_ as well as DOX loading.	
Hong H. et al. ACS Nano (2012)	GO	GO	AFM: GO-PEG- NH_2_, NOTA-GO & NOTA-GO-TRC105 have size= 0 - 50 nm.			Zeta: GO-PEG- NH_2_= -4.8 mV, NOTA-GO-TRC105= -0.1 mV; DLS: GO-PEG- NH_2_= 22 nm, NOTA-GO-TRC105= 27 nm
Kim H. et al Bioconjugate Chem. (2011)	GO	GO-BPEI	AFM: GO size= 500-600 nm & height= 0.6-1.3 nm, BPEI-GO/pDNA size= 300-400 nm & height=16-18 nm.		FT-IR&UV-Vis: confirmation of BPEI functionalisation	Zeta: GO=-30 mV, GO-BPEI= +40 mV; Stability: stable in water, PBS & DMEM media for 1 hr
Sun X. et al Nano Res. (2008)	NGO	nGO-PEG	AFM: GO size= 10-300 nm & height= ~1.0 nm, NGO-PEG mostly <20 nm.		FT-IR: confirms PEG functionalisation; UV-Vis: confirms conjugation.	
Li P. et al. Biomater. Sci. (2018)	NGO	UCNP@NGO	TEM: UCNPs size= ~55 nm & NGO size= ~200 nm		FT-IR: to confirm coordination of UCNP on NGO;	DLS: NGO= ~100 nm & UCNP@NGO= ~200 nm; TGA: 5 wt% of OA & 34wt% of UCNPs on NGO.
Bi H. et al. ACS publications (2018)	GO	GO/ZnFe_2_O_4_/UCNPs (GZUC)	AFM: GO height= 1-2 nm; TEM: UCNPs high monodispersity & size <10 nm	ICP-MS: mass ratio GO:UCNPs:ZnFe_2_O_4_ = ~1:2:2; XPS: Zn & Fe peaks; XRD: confirms ZnFe_2_O_4_ & hexagonal NaGdF4 in GZUC	UV-Vis: strong absorption peak of GO	Zeta: UCNPs = ~-12 mV, GZUC-PEG = ~-18 mV; DLS: GZUC-PEG= ~400 nm.
Gulzar A et al. Dalton Trans (2018)	NGO	NGO-UCNP-Ce6 (NUC)	AFM&TEM: NGO-PEG size = ~100 nm, single layer.	XRD: confirms UCNPs	UV-Vis: confirms Ce6 loading on UCNPs-NGO; FT-IR: confirms PEGylation	
Zhang Y. et al. J. Mater. Chem. B. (2017)	GO	GO/Bi_2_Se_3_/PVP	AFM&TEM: GO size = 100-500 nm & height = ~1 nm; HRTEM: NPs size = ~3-9 nm.	EDS: confirms C, Bi & Se in GO/Bi_2_Se_3_/PVP; XRD: GO peak @ 11.4°	FT-IR: confirms GO & functionalisation	Zeta: GO= -18 mV; DLS: GO/Bi_2_Se_3_/PVP= ~ 149 nm
Gao S et al Biomaterials (2016)	GO	CPGA	TEM: CPGA size= ~230 nm, NP loading shown; AFM: CPGA height= ~15 nm.		UV-Vis confirms composite formation	Zeta: CPGA= -25 mV; DLS: ~230 nm; Stability: 1 d storage (UV-vis-NIR).
Kalluru P et al. Biomaterials (2016)	NGO	GO-PEG-folate-mediated NmPDT	AFM&TEM: ~100 nm size		RS: D & G peak visible; FT-IR: confirms composite; UV-Vis: confirms functionalization of GO	DLS: ~100 nm
Lin LS et al. Nanoscale. (2016)	rGO	PEG-rGO-GSPs	TEM & AFM: GO size= ~150 nm, GNPs= ~14 nm		UV-Vis: confirms reduction of GO; RS: broad D & G peak visible	DLS: 3 GO-GSPs with size: 60 nm, 90 nm & 130 nm; Stability: stable in water, PBS, CCM & FBS for 3 d
Luo S et al. ACS Appl Mater Interfaces. (2016)	NGO	NGO-808	AFM&TEM: NGO size= 100-500 nm & height= 1 nm, NGO-808 size = 20 40 nm & height= ~3 nm.		FT-IR: confirms functionalization; 1H NMR: confirms PEG functionalization	
Hu D et al Theranostics. (2016)	GO	ICG-PDA-rGO	AFM: GO size <1 µm, height= ~0.86 nm, ICG-PDA-rGO no size change, height>2 nm.	XPS: shows some GO reduction after functionalisation.	UV-Vis: confirms conjugation with ICG	
Huang G et al Nanoscale (2015)	GO	IO/GO-COOH	AFM&TEM: GO-COOH = 300-600 nm size & height= ~2 nm, IO-13/GO-COOH similar size & height= ~25 nm.		FT-IR: to show COOH functionalisation.	Stability: no aggregation for 30d
Kim YK et al. Small. (2015)	GO	PEG-Au@GON NPs	AFM&TEM: GON size= 10 -15 nm & height= 1.2 nm; TEM: spherical shape of Au@GON size= ~60 nm, NPs with 2-3 nm thick GON shell.	Elemental analysis: C/O ratio=0.40; XPS: GO & Au peaks observed.	FT-IR: confirms conjugation of Au; RS: I(D)/I(G) of GON= 0.79 & Au@GON =0.83.	Zeta: Au@GON NPs= -58 mV, GON=-47 mV; Stability: PEGylated composite showed better stability after 20min storage (UV-Vis)
Rong P. et al. RSC Adv. (2015)	GO	GO-PEG-CysCOOH	AFM: GO size = ~200 nm, GO-PEG size <~50 nm & height= ~1.5 nm, GO-PEG-CySCOOH height = ~2 nm.		UV-Vis: Confirms successful functionalization;	Stability: Good stability in water, PBS, cell medium, FBS
Yan X et al. Nanoscale. (2015)	GO	GO-PEG-DVDMS	AFM: GO-PEG size= ~ 14 nm & height= 1.3 nm. GO-PEG-DVDMS size= ~ 20.5 nm & height= 1.5 nm.		UV-Vis: confirms functionalization with DVDMS	
Zhang H et al. Biomaterials. (2015)	GO	GO/BaGdF5/PEG	TEM&AFM: GO size= ∼ 200 nm & height= ~1.0 nm, smaller size of GO/BaGdF5/PEG.	XRD: confirms BaGdF5 formation; GO peak @ 11.3° disappears	FT-IR: confirms functionalization; UV-Vis: confirms reduction of GO	Zeta: GO= -20 mV; Stability: good stability in water, saline, PBS, RPMI-1640 & FBS; TGA: BaGdF5= ~42 wt%
Gollavelli G. et al Biomaterials (2014)	MGF	MFG	AFM: size= ∼40 nm		FT-IR: confirms functionalization	Stability: MFG-SiNc4 stable in water & in biological media
Viraka Nellore et al. Faraday Discuss. (2014)	GO	ICG-FeCl_3_ @GO	TEM: NP size= ~40 nm, confirms conjugation.		RS: confirms GO	TGA: magnetic NPs= 30 wt% & GO= 70 wt %.
Nergiz S.Z. et al ACS Appl. Mater. Interfaces (2014)	GO	GO & GOAuNS	AFM: GO size= ∼0.5 μm & height= 1 nm. No change after Au NPs formation.		RS (785 nm): confirms Gr; no large changes after Au NPs formation.	Zeta: GO= -35 mV, GOAu= -25 mV; stable @ ~-20 mV in 10% FBS; Stability: excellent stability in 10% FBS over a course of 10 d.
Wang S. et al Biomaterials (2014)	GO	GO-ION-PEG	TEM: ION uniformly grew; AFM: NS size < 200 nm & height= 3-10 nm.	ICP-AES: Fe =55.6 wt% in GO-IONP.	RS: confirms GO & NPs; UV-Vis: confirms reduction of GO; FT-IR: confirms fucntionalization with PEG	DLS: ~166 nm; Stability: good stability in water & physiological solutions for 72 h
Rong P et al. ADV Theranostics (2014)	GO	GO-PEG-HPPH	AFM: GO-PEG size< 50 nm & height= ~1.5 nm; GO-PEG-HPPH height= ~2 nm.		UV-Vis: confirms loading of HPPH	Stability: good stbaility in water, PBS, cell medium & serum
Cho Y et al. Chem Commun (Camb) (2013)	GO	GO-HA-Ce6	SEM: 500-700nm size (one picture)			DLS: GO complex= ~441 nm
Shi X et al. Biomaterials. (2013)	GO	GO-IONP-Au-PEG	AFM: GO & GO-IONP size= ~200-600 nm.			Zeta: GO-IONP= -35 mV, GO-IONP-Au-PEG= -5.6 mV; Stability: GO-IONP-Au-PEG stable in water, saline & serum solutions.
Wang Y.W. et al. J. Mater. Chem. B(2013)	rGO	ICG-Rgo-FA	AFM: GO size = ~200 nm & height = ~1 nm			
Sheng Z. et al Biomaterials (2013)	nano-rGO	BSA/nano-rGO	AFM: height=4 nm; size = ~70 nm	XPS: confirms reduction	UV-Vis: confirms BSA absorption	Stability: BSA/nano-rGO stable in buffers & other biological solutions for one month's storage
Yang K. et al. Adv. Mat. (2012)	rGO	RGO-IONP-PEG	TEM: IONPs size= 8-10 nm, even distribution on RGO; AFM&DLS: RGO-ION size> 200 nm; RGO-IONP-PEG size= ~50 nm	XPS: confirms reduction after IONPs growth; ICP-MS: RGO:IONP weight ratio= 1:1.99; XRD: confirms IONP formation	FT-IR: confirms PEG functionalization; NMR: ~ 20% of carboxyl groups conjugated with PEG	Stability: RGO-IONP-PEG stable in water, saline & serum solutions
Hu S.H. et al Adv. Mat. (2012)	rGO	rGO-QD	TEM: well‐ordered QDs (size= 3.6 nm) array separated by gaps of 1.7 nm.			DLS: Three batches with different size: ~2.5 μm; ~260 nm; ~38 nm; Stability: QD‐rGO highly stable.
Yang K. et al Nano Lett. (2010)	NGS	NGS-PEG	AFM: size= 10-50 nm & height= ~1 nm		FT-IR: confirms functionalization	Stability: good stability of functionalized GO
Karimi Shervedani R et al. Biosens Bioelectron. (2018)	RGO	RGO-PDA-BSA-DTPA-Mn(II)/MTX	AFM: GO NS height = 3.9 nm; RGO-PDA-BSA height= 17 nm. Smooth surface/good distribution of Mn(II)/MTX (height= ~0.9 nm).		FT-IR: confirms functionalization; After functionalization of GO with PDA, reduction is confirmed.	
Chang X. et al. Carbon (2018)	GO	GO/MnWO_4_/PEG	TEM: GO size = ~130 nm, MnWO_4_ NPs size= ~12 nm; AFM: GO/MnWO4/PEG height=~16 nm, GO height= ~3.4 nm; SEM: confirms conjugated morphology	XPS: Mn, W, C, O, & N; XRD: confirms MnWO_4_	FT-IR & UV-Vis-NIR: confirms GO & conjuataion	Zeta: GO = ~-42 mV, GO/MnWO_4_/PEG = ~-26 mV; TGA: 59.6 wt % of MnWO4 & 13.0 wt% of GO in GO/MnWO_4_/PEG.
Gulzar A et al. Dalton Trans (2018)	NGO	UCNPs-DPA-NGO-PEG-BPEI-DOX	AFM: height= 30 nm; TEM: size= ~100 nm		FT-IR: confirms PEG functionalization	
Wu C et al. Acta Biomater. (2017)	GO	GO/AuNS-PEG & GO/AuNS-PEG/Ce6	TEM: GO size = ~ 2 μm, Au NP coating (~40 nm in size)AFM: GO/AuNS-PEG/Ce6 height = ~18 nm & size= ~400 nm.		UV-vis: confirms Au NPs on GO	Zeta: GO/AuNS-PEG/Ce6= -38 mV, GO/AuNS-PEG= -20 mV; Stability: GO/AuNS-PEG stable; TGA: content of PEG is ~40%
Battogtokh G. et al. J. Control. Resease(2016)	GO	PheoA + GO:FA-BSA-c-PheoA NC			UV-Vis: confirms conjugation;	Zeta: GO NP= -24 mV; DLS: GO = ~249 nm, PheoA+GO:FA-BSA-c-PheoA NC (0.5:1 w/w)= 182 nm
Justin R et al. Carbon (2016)	GQDs	MGQDs-LH	TEM: Iron oxide coating on Gr surface; HRTEM: several layers rGO, wrinkles; AFM: flakes diameter= 45nm & height= 2.3 nm.		RS (514.5 nm): very small & broad G peak, intense & very broad D peak; FT-IR: confirms Iron oxide formation	DLS: ~61 nm
Chen Y.W. et al. Small (2016)	rGO	anti‐EGFR‐PEG‐rGO@CPSS‐Au‐R6G	TEM: GO/silica NS height = ~44 nm. SEM: confirms morphology of rGO@CPSS-x, size = ~160 nm	EDX: Si, O, & C in GO/silica. Au in rGO@CPSS-6-Au; XRD: confirms composite	UV-Vis & FT-IR: confirms conjugation with Au NPs.	
Chen H et al Theranostics. (2016)	GO	GO-Au@PANI/DOX	AFM: GO-PVP height= ~1.3 nm; TEM: unevent deposition on GO		RS (785 nm): GO peaks not visible due to polymer	Stability: GO-Au@PANI very stable in water, PBS & RPMI-1640
Shi J et al. Pharm Res. (2016)	GO	GO@Gd-PEG-FA/DOX	TEM: GO@Gd-PEG-FA/DOX size is much smaller than GO@Gd.		UV-Vis&FT-IR: both confirm functionalization with PEG & FA	Zeta: GO@Gd-PEG-FA/DOX =-6 mV; DLS: GO-Au@PANI= 120 nm; Stability: GO@Gd-PEG-FA very stable in water & physiological solutions; TGA: PEG in GO@Gd= 15.2 wt%
Miao W et al. J Control Release. (2015)	rGO	ICG/HArGO & ICG/rGO	TEM: shows four flakes with size ~ 100nm.			Zeta: rGO= -30 mV; all hybrids= -50-60 mV; DLS: ~100 nm for all materials.
Yan X et al. Biomaterials. (2015)	GO	GO-PEG-DVDMS	AFM: GO-PEG size <50 nm & height= ∼1.5 nm; GO-PEG-DVDMS height=∼2 nm & the size x2 larger.		UV-Vis: shows a new peak for GO-PEG-DVDMS;	
Chen H. et al. RCS Adv. (2015)	rGO	DOX-rGO-Fe_2_O_3_@Au NPs	TEM: NH_2_-PEG-Fe_2_O_3_@Au NPs = ~20 nm & rGO-COOH = ~1 µm. Conjugation morphology confirmed.		UV-Vis: to confirm conjugation with NH_2_-PEG-Fe_2_O_3_@AuNP; RS: confirms GO	Zeta: rGO-COOH = -29.5 mV, rGO-Fe_2_O_3_@Au NPs = -21.1 mV; DLS: rGO-COOH= 616 nm, rGO-Fe_2_O_3_@Au NPs= 612 nm; Stability: Stable in DMEM + 10% (v/v) FBS for 5 h
Khatun Z. et al. Nanoscale (2015)	Gr	GDH	TEM: Gr size= ~5-10 nm, GDH nanogel size= ~100 nm		FT-IR: confirms conjugation	Stability: stable in PBS, pH5 & 10% FBS solution for 7 d.
Yang Y et al. J. Biomater. Appl.(2015)	GO	GO/MnFe_2_O_4_/DOX	TEM: GO size= 50-500 nm & height= 0.8-1.1 nm; GO/MnFe_2_O_4_ size= 70-310 nm.	XRD: MnFe_2_O_4_ deposition confirmed.		Stability: The hybrids can be well dispersed in water, physiological saline, & FBS
Bian X. et al Sci. Rep. (2014)	Gr	NGsAu nanocrystal	TEM: core-shell size= 65 nm. Few-layers Gr as uniform coating on the whole particle.		RS (633 nm): I(D) ≈ I(G), indicating high defect concentration.	Zeta: ~0 mV; DLS: confirms particles size; Stability: functionalization increases stability.
Feng L et al. Adv Healthc Mater. (2014)	NGO	GO-PEG-DA	AFM: size of 50-100 nm.		UV-Vis: confirms functionalization	Zeta: GO= -40 mV; NGO-PEG-DA= -35 mV; DLS: confirms stability; Stability: all stable in physiological solutions; TGA: PEG content= ~ 25.3%
Shi J. et al Biomaterials (2014)	GO	GO-Ag	TEM: confirms presence of Ag NPs with diameters of 5-15 nm	XRD: GO peak @ 10.9°, disappeared completely in GO-Ag.	UV-Vis: confirms Ag NPs; FT-IR: decrease in O-H peak after activation; confirmed DOX conjugation	Zeta: GO-Ag-DOX-NGR= -30 mV
Jin Y. et al. Biomaterials (2013)	GO	Au@PLA-(PAH/GO)n	TEM: GNP size = ~2.6 - 2.8 nm, TEM & SEM: microcapsule morphology, size = ~1 µm		UV-Vis & FT-IR: confirm conjugation	
Wang Y. et al J.Am.Chem.Soc (2013)	Gr	GSPI	AFM: GO size= ~50-250 nm; TEM: confirms mesopores with size of 50-150 nm; HRTEM: pore depth= ~25 nm.	XRD: Gr is coated with a hexagonal array of pores with ordered structure.	RS (633 nm): D peak indicates defective Gr; FT-IR: indicates GO reduction & confirms functionalization.	Zeta: GO = -52 mV; GO-PEG = -38 mV; stability: GSPI retains water dispersion.
Wang Y. et al Biomaterials (2013)	GO	GO-UCNPs--ZnPc	GO-PEG height= ∼1.2 nm, size = 100-250 nm		FT-IR: confirms PEG functionalization	
Miao W. et al Biomaterials (2013)	GO	Ce6/Dox/pGO	AFM: pGO NS height= ~1 nm & Ce6/Dox/pGO size = ~148nm		FT-IR: confirms functionalization in pGO	Zeta: ~-41 mV for all complexes.Note: inconsistency between AFM & DLS size data of pGO.
Qin H et al. Small (2015)	GO	(GO-dye: GO-RhB, GO-Cy5, & GO-Cy7)GO-Abs/Cy7				Stability: All stable in physiological solutions (but not NGO)
Hatamie S et al Colloids Surf B Biointerfaces. (2016)	GO	Gr/cobalt nanocomposites	TEM: confirms sheet decorated by the NPs.	XPS: O/C ratio=0.9 for GO, 0.18 for composites; XRD: indicates GO reduction after hybrid formation.	FT-IR&UV-vis: indicates GO reduction after hybrid formation; RS(532 nm): confirms reduction (although not complete)	Zeta: ~ -18 mV (in PBS)
Chen L et al. Biomaterials (2015)	RGO	^131^I-RGO-PEG	AFM: RGO-PEG size=∼40-60 nm & heights= 3-4 nm.			Stability: GO-PEG is stable in water, saline, serum; ^131^I-RGO-PEG is stable in 9% NaCl, FBS, PBS, & RPMI-1640 for 7 d

**Table 5B:** Table summarizing the different methodologies for the characterization of new 2DMs.

Ref	Material		Characterisation Techniques			
	2DMs	Acronym	Microscopy	Elemental Analysis/ Diffraction	Spectroscopy	Zeta Potential/ DLS/Stability/Others
Yang G. et al.Small (2018)	WS_2_	WS_2_-IO/S@MO-PEG	TEM (HAADF-STEM): WS_2_ size= ~50 nm.	Elemental mapping: Weight ratio of W:Si:Fe:Mn = 1:5.02:4.02:7.6	UV-vis: confirms composite formation	Stability: WS_2_-IO@MO-PEG in water, PBS, FBS, CCM for 24hr shows good stability (DLS)
Chao Y. et al.Small (2016)	WS_2_	^188^Re-WS_2_-PEG	TEM (DLS): WS_2_ -PEG size= 80-150 nm		UV-Vis: WS_2_- PEG do not show any feature	Stability: PEGylated nanoflakes exhibit high stability in various physiological solutions
Yong Y. et al.Nanoscale (2014)	WS_2_	BSA-WS_2_	TEM: WS_2_ size= 20-100 nm; AFM: WS_2_ height= ~ 1.6 nm, BSA-WS_2_ height= 4-5 nm.	EDS: W, S, C; XRD: confirm hexagonal structure of WS_2_	FT-IR: confirms functionalization with BSA; UV-Vis: sharp peak @ ~610 nm; RS: peaks are broader compared to bulk WS_2_.	Stability: BSA-WS_2_ shows good stability in PBS
Cheng L. et al.Adv. Mater. (2013)	WS_2_	WS_2_-PEG	AFM: WS_2_ height= ~ 1.1 nm. WS_2_- PEG height = ∼1.6 nm.; TEM: WS_2_ -PEG size= 50-100 nm	EDS: W & S; XRD: confirms hexagonal structure of WS_2_.	UV-Vis: WS_2_-PEG NS show typical features observed for 2H-WS_2_ phase.	Stability: WS_2_ PEG exhibits excellent stability in various physiological solutions
Liu J. et al.J. Colloid Interface Sci. (2019)	MoS_2_	MoS_2_-HA-DTPA-Gd	AFM(DLS): MoS_2_ & MoS_2_-HA- NH_2_ sheet-like morphology. MoS_2_ NS height= ∼1-2 nm. MoS_2_-HA height= ∼3-4 nm. MoS_2_= ~100 nm, MoS_2_-HA- NH_2_ size= ~200 nm	EDS&XPS: confirms even distribution of Gd on the surface of the MoS_2_.	FT-IR & UV-Vis: confirm functionalization with HA; UV-Vis: different from 2H MoS_2_ -no exciton peaks visible.	Stability: MoS_2_-HA- NH_2_ exhibited enhanced stability in water, PBS & cell medium; TGA: HA-SS- NH_2_= ∼67.4 wt% in MoS_2_-HA- NH_2_.
Jing X. et al. Bioconjugate Chemistry (2018)	MoS_2_	Mn-doped Fe_3_O_4_@MoS_2_ nanoflower	TEM: Mn-Fe_3_O_4_@MoS_2_ has a petal shape, a rough & folded surface structure; DF-SEM: confirms Mn-doped Fe_3_O_4_ is located at the core of the flower-like MoS_2_ NS.	XPS: confirms MoS_2_, & Fe_3_O_4_; confirms Mn doping; XRD: confirms magnetite Fe_3_O_4_ & MnFe_2_O_4_. Diffraction peaks of pristine 2H-MoS_2_ of Mn-doped Fe_3_O_4_.	RS(514 nm): confirms heterojunctions between MoS_2_ & Mn-doped Fe_3_O_4_, MoS_2_ peaks. FT-IR& UV-vis: confirm functionalization.	Zeta : Mn-doped Fe3O4= -26 mV; DLS: all hybrids = 200-1000 nm. Stability: all stable for 5 d in DI water. The composites are stable in DI water & PBS for 5 d.
Maji S. et al. ACS Appl. Mater. Interfaces(2018)	MoS_2_	AuNBPs@MoS_2_	SEM: 1 NS of size= ~80 nm; TEM: thin layer of MoS_2_ with 1.5-2 nm observed on AuNP surface.	EDX: confirms the formation of core@shell AuNBPs@MoS_2_.	RS: MoS_2_ peaks visible in AuNBPs@MoS_2_; UV-Vis: no excitons peak observed.	
Dong X. et al. ACS Appl. Mater. Interfaces (2018)	MoS_2_	MoS_2_-PEI-HA	AFM: MoS_2_ height= ~0.8 nm, MoS_2_-PEI-HA height= 5-7 nm; TEM: MoS_2_ size= ~30-50 nm.		RS (514 nm): a slight shift compared to the bare MoS_2_; FT-IR: confirms conjugation of PEI & HA; UV-Vis: exciton peaks of MoS_2_ visible in conjugated material	Zeta: MoS_2_= ~-20 mV, MoS_2_-PEI-HA= -18 mV; Stability: improved stability after HA modification in water & other physiological solution.
Chen L. et al. ACS Appl. Mater. Interfaces (2017)	MoS_2_	MoS_2_-Gd-BSA	MoS_2_ MoS_2_ & MoS_2_-Gd-BSA - composed of several lamellar structures, forming cluster.	XPS: confirms MoS_2_.	RS(633 nm): typical MoS_2_ peaks; FT-IR: confirms functionalisation.	Zeta: MoS_2_= -30 mV, MoS_2_-Gd-BSA = -26.4 mV; DLS: MoS_2_= 205 nm, MoS_2_-Gd-BSA= 297 nm; Stability: MoS_2_-Gd-BSA in PBS & CCM; TGA: confirms functionalisation.
Liu B. et al. Adv. Sci.(2017)	MoS_2_	MoS_2_@Fe_3_O_4_-ICG/Pt(IV)Nanoflowers	SEM&TEM: composite size ~80 - 180 nm, no morphology change with functionalization. HRTEM&HAADF/ STEM-EDS: confirm Fe_3_O_4_.	EDS: Mo, S, N, Fe & O. ICP-MS: 5.1 wt% of Pt; XRD: shows 2H-MoS_2_ phase	FT-IR&UV-Vis: confirm functionalization with ICG	Zeta: MoS_2_= -25 mV, MoS_2_-PEI= 27 mV; Stability: no aggregation for 2 d; TGA: 27.8wt% of PEI
Xu J. et al. Small (2017)	MoS_2_	MoS_2_	SEM&TEM: bulk MoS_2_ flower structure & NS size=~500 nm, spherical UCS size= 48 nm & composite structure with UCS particles on NS are shown.	EDS&XPS: confirm successful conjugation; XRD: confirms successful conjugation	FT-IR&UV-Vis: confirms successful functionalisation & conjugation	Zeta: MoS_2_= -25 mV, MUCS = +5 mV; Stability: MUCS in DMEM for 1d (UV-Vis)
Zhao J. et al. Oncotarget (2017)	MoS_2_	MoS_2_-PVP	TEM: MoS_2_ size= ~50 nm, MoS_2_-PVP size= 21 nm, height= ~ 1 nm, for higher PVP content size = ~15 nm	XPS&EDS: confirms MoS_2_, EDS: Mo, S, C & O; XRD: confirms crystalline structure of MoS_2_	FT-IR: confirms successful functionalisation.	Stability: MoS_2_-PVP in water, saline, CCM, & FBS showed good stability for 3 d
Li X. et al. Int. J. Nanomed (2016)	MoS_2_	SP-MoS_2_	TEM&FESEM: crumpled sheet structure of SP-MoS_2_ size= ~50 nm		UV-Vis: SP-MoS_2_ peak ~ ~300nm	Stability: SP-MoS_2_ stable in water, saline, & RPMI-1640 for 2d (DLS)
Yu J. et al. Theranostics (2015)	MoS_2_	MoS_2_/Fe_3_O_4_	SEM&TEM&AFM: MoS_2_ size= ~100 nm, & height= 8-12 nm	EDX&XPS: show possible surface oxidation of MoS_2_	FT-IR: confirms conjugation; RS: MoS_2_ peaks visible	Zeta: MSIO= -22 mV, MoS_2_= -23 mV; DLS: MSIOs = ~190 nm in water& PBS for 2 d; Stability: MSIO in DMEM, FBS.
Wang S. et al. Adv. Mater.(2015)	MoS_2_	MoS_2_/Bi_2_S_3_	FESEM&HRTEM: crumpled & defective MBP size= ~300 nm, MoS_2_ crystal lattice= 0.26 nm	EDS: Mo, Bi, S, & O; XPS: confirms composite formation; XRD: confirms high purity of the composite.	FT-IR: confrims PEG functionalisation	TGA: 20.7 wt% of PEG (100-350°C)
Wang S. et al. Biomaterials (2015)	MoS_2_	PEGylated MoS_2_	FESEM&TEM: MoS_2_ NS size= ~50 nm & height= 0.25 nm, MD300-PEG size= ~297 nm & height= 0.42 nm.	EDS: Mo & S for MoS_2_, additional C & O for PEGylated composites; XPS: confirm PEGylated composite; SAED: confirms crystallinity; XRD: confirms PEGylated composites.	FT-IR: confirms PEGylation	Stability: No change in DLS in saline after 24hr storage
Liu T. et al. ACS Nano (2015)	MoS_2_	MoS_2_-IO-(d)PEG	HRTEM: MoS_2_ NS size= 50-200 nm, confirms MoS_2_ hexagonal structure.	EDS: Mo, S, Fe, & O; XRD: confirms MoS_2_ NS.		Zeta: MoS_2_= -12 mV, MoS_2_-IO-(d)PEG = +2 mV; DLS: ~100 nm size for all composites; stability: good stability for MoS_2_-IO; TGA: confirms PEGylation
Liu T. et al. Nanoscale (2014)	MoS_2_	MoS_2_-PEG	AFM: MoS_2_ size= ~100 nm & height= ~1 nm, MoS2-PEG height= ~2 - 10 nm.		UV-Vis: confirms functionalisation	DLS: ~200 nm; Stability: MoS_2_ & MoS_2_-PEG in water& PBS. MoS_2_ unstable in PBS, but the others stable.
Li Z. et al. ACS Appl. Mater. Interfaces (2019)	BP	RP-p-BPNSs	TEM & AFM: p-BPNSs size <200 nm & height= ~1.3 nm; HRTEM: 0.22 nm lattice fringe; TEM: morphology change of BPNS after 7 d, no change of p-BPNS.	XPS: confirms no oxidation in p-BPNS	RS (633 nm): confirms BPNS; UV-Vis: confirms no degradation of p-BPNS; FT-IR: confirms conjugation	Zeta: p-BPNSs= -40 mV; DLS: p-BPNSs= ~200 nm; Stability: stable in water, PBS, RPMI & FBS.
Huang H. et al. Biomaterials (2018)	BP	BP/Bi_2_O_3_	TEM & AFM: BP size= ~500 nm, Bi_2_O_3_ NPs size = 5 nm, BP/Bi_2_O_3_, size = 300 nm & height = 25 nm; HRTEM: 0.21 nm d-spacing of BP	EDS: BP/Bi_2_O_3_ = P 36.8%, O 56.5%, & Bi 6.7; XPS: P ~35.51%, O ~54.99% &~Bi 9.5%.; XRD: confirms Bi_2_O_3_		Zeta: BP is -27 mV, BP/Bi_2_O_3_ is +34 mV.; Stability: BP unstable but BP/Bi_2_O_3_ stable for 8 d (optical, absoprtion spectroscopy)
Qiu M. et al. Proc Natl Acad Sc (2018)	BP	BP@Hydrogel	TEM & AFM: BPNSs size = ~100-200 nm & height = 2.6 nm; HRTEM: d-spacing of BPNSs 0.34 & 0.42 nm	XPS: confirms BP	RS (514 nm): confirms BP in composites	Zeta: BPNS= -28 mV & PEG-BPNSs= -17 mV; DLS: BPNSs =156 nm & PEG- BPNSs = 160 nm
Yang X. et al. ACS Appl. Mater. Interfaces (2018)	BP	BP@PEG/Ce6 NSs	TEM &AFM: BP size= 90 nm, height= 14.3 nm, BP-PEG size= 100 nm, height= 15 nm.	EDX: confirms mostly P & little oxidation; XRD: confirms BP crystallinity	RS (633 nm): confirms BP; UV-Vis-NIR: confirms loading of Ce6	Zeta: BP NSs = ~-17 mV, BP@PEG/Ce6 NSs = ~-8 mV; DLS: BP NSs & BP@PEG NSs= ~ 90 nm, BP@PEG/Ce6 NSs = 158 nm; stability: BP & composites in water & in PBS confirmed for 24 hr;
Yang B. et al. Adv. Mater.(2018)	BP	BP-BG scaffold	TEM &AFM: BP siz=e ~500 nm, height <10 nm, HRTEM: 0.32 nm d-spacing; TEM: morphology change after biomineralizaion	XPS: shows very small amount of BP compared to P_2_O_5_, increased O & Ca% after biomineralization; SAED: confirms BP crystal; XRD: confirms SiO_2_ (cristobalite)	RS & FT-IR: confirms biomineralization	Stability: stability of BP in SBF after 4 d shows flocculation & biomineralisation
Chen W. et al. Adv. Mater. (2017)	BP	BP	TEM & AFM: BP size= ~200 nm, height= ~5.5 nm.	XPS: confirms BP with small oxidation	UV-Vis & FT-IR: confirm drug loading	DLS: BP = 281 nm; Stability: stable in water for 24 hr
Tao W. et al. Adv. Mater.(2017)	BP	BP-PEG-FA/Cy7 NSs	TEM&AFM: BP size =~120 nm & height =~1-2 nm, BP-PEG size = ~100 nm & height = ~2-3 nm	XPS: P, C, & O in BP-PEG; STEM-EDS: C, O, N & P in BP-PEG-FA; XRD: confirms orthorhombic BP in BP-PEG	FT-IR: confirms functionalisation; RS: confirms BP in composites	Zeta: BP = -13 mV, BP-PEG-FA = -17 mV; Stability: BP-PEG stable in PBS & FBS (DLS= ~130 nm) for 7 d
Yang G. et al. Biomaterials Science (2017)	BP	BP-Au NSs	TEM: AuNPs size= 26 nm & BP-Au size= 484 nm.		UV-Vis: confirms conjugation with Au; RS (514.5 nm): confirms BP in composite	Stability: stability of BP-Au & BP-Au-/PEG in PBS solution
Xiang H. et al. ACS Nano (2019)	Mxene	AIPH@Nb_2_C@mSiO_2_	TEM: Nb_2_C size= 100-300 nm, morphology change observed with functionalisation	XPS: confirms Nb (8.41%), C (25.96%), Si (25.96%), & O (52.68%) in Nb_2_C@mSiO_2_ NPs.; XRD: confirms amorphous SiO_2_	FT-IR & UV-Vis: confirms AIPH functionalisation	Zeta: Nb_2_C = -39 mV, AIPH@Nb_2_C@mSiO_2_ = -10 mV; DLS: Nb_2_C = 219 nm, AIPH@Nb_2_C@mSiO_2_ = 308 nm.
Tang W. et al. ACS Nano (2019)	Mxene	Ti_3_C_2_@Au	TEM&SEM&AFM: Ti_3_C_2_ size = ~200 nm & height = ~2 nm; TEM: Au shell coating of ~30 nm height	EDS: confirms Au on Ti_3_C_2_; XRD: confirms Ti_3_C_2_ Mxene	UV-Vis-NIR: confirms conjugation with Au.	Zeta: Ti_3_C_2_ = -24 mV, Ti_3_C_2_-PAH-Au = -20 mv; DLS: Bi_3_C_2_= ~100 nm, Bi_3_C_2_@Au= 150 nm; Stability: Ti_3_C_2_@Au-PEG in water, PBS, FBS & RPMI confirmed.
Liu Z. et al.Theranostics (2018)	Mxene	Ta_4_C_3_-IONP-SPs	TEM: Ta_4_C_3_ NS size = ~50-200 nm, Ta_4_C_3_-IONPs size = ~50 - 200 nm; HRTEM: d-spacing Ta_4_C_3_= 0.26 nm, IONP = 0.30 nm	EDS: confirms Ta, C, O, & Fe in Ta_4_C_3_-IONPs composite; XPS: confirms conjugation with IONPs; XRD: confirms Fe_3_O_4_	FT-IR: confirms conjugation	Zeta: Ta_4_C_3_ =-24 mV & Ta_4_C_3_-IONP-SPs = -49 mV; Stability: Improved stability with surface modification in water, DMEM, PBS, saline, & SBF over 3 d; DLS: Ta_4_C_3_-IONP-SP = 255 nm, Ta_4_C_3_-IONP = 190 nm.
Han X. et al.Theranostics (2018)	Mxene	CTAC@Nb_2_C-MSN	TEM: Nb_2_C NS size= several hundreds nm; SEM&TEM: CTAC@Nb_2_C-MSN composite of mesoporous morphology	EDS&XPS: confirms conjugation; SAED&XRD: confirms retained high crystallinity		DLS: Nb_2_C = 91 nm, CTAC@Nb_2_C-MSN-PEG-RGD = ~220 nm; Stability: stable in water, DMEM, saline, SBF, & PBS (DLS)
Li Z. et al. Adv. Mater.(2018)	Mxene	Ti_3_C_2_@mMSNs	TEM&SEM: Ti_3_C_2_ NS size= 50-100 nm with mesoporous morphology	EDS&XPS: confirms conjugation	FT-IR: confirms functionalization.	Zeta: Ti_3_C_2_ = -20 mV, Ti_3_C_2_@mMSNs-RGD = 2 mV; DLS: Ti_3_C_2_ =92 nm, Ti_3_C_2_@mMSNs-RGD = 152.9 nm; BET: surface area 772 m^2^/g, pore size 3.1 nm
Han X. et al. Adv. Healthc. Mater.(2018)	Mxene	Ti_3_C_2_-SP	SEM&TEM&AGM: Ti_3_C_2_ NS size = ~120 nm & height =~0.9 nm; HRTEM: comfirms cyrstallinity	EDS & EELS: confirms complete removal of Al		Zeta: Ti_3_C_2_ = -52 mV, Dox@Ti_3_C_2_-SP = -29 mV; DLS: Ti_3_C_2_ = 122 nm, Ti_3_C_2_-SP = 164 nm.
Liu et al. AM&I (2017)	Mxene	Ti_3_C_2_@DOX@HA	TEM&AFM: Ti_3_C_2_ size = ~100 nm & height=1-2 nm.	XPS: confirms Ti-C & Ti-O in Ti_3_C_2_; ICP: ratio of Al:Ti = 0.62:3; XRD: confirms Ti_3_C_2_ & conjugation	UV-Vis: confirms conjugation of Al^3+^	Zeta: Ti_3_C_2_ = -23 mV, Ti_3_C_2_@DOX@HA = -21 mV; DLS: Ti3C2 = 104 nm, Ti_3_C_2_@DOX@HA = 178 nm; Stability: Ti_3_C_2_ in water, PBS compared with surface modified Ti_3_C_2_
Lin H. et al. JACS (2017)	Mxene	Nb_2_C-PVP	SEM&TEM&AFM: Nb_2_C NSs size= ∼150 nm & height= ~0.3-0.8 nm	EDS&EELS&XPS: confirms no Al & presence of Ni, C & O; XPS: confirms oxidation; SAED: confirms hexagonal symmetry; XRD: Nb_2_AlC MAX phase	RS: confirms elimination of Al layer; FT-IR & UV-Vis-NIR: confirms functionalisation with PVP;	Zeta: Nb_2_c = -50 mV; TGA: PVP loading 20 wt%
Dai C. et al. ACS Nano (2017)	Mxene	MnOx/Ta_4_C_3_-SP	SEM&TEM: Ta_4_C_3_ NS size= ~50 - 100 nm	EDS&XPS: confirms Ta, Mn & O in MnOx/Ta_3_C_3_; XRD: confirms removal of Al & 2D crystallinity		

**Table 6:** Table showing the main advantages and disadvantages of GRMs for cancer theranostics applications (Y=yes, N=no).

Property	GO	rGO	Graphene	Other 2DMs
High surface area	Y	Y	Y	Y
Defective	Y, high	Y, low	N	Depends on the 2D material
Functionalization	Very easy	Easy	Possible, but more difficult than GO or rGO	Some functionalization routes existing, depending on the 2D material
Dispersibility in water	Y	N	N	No, with the exception of few 2D materials (e.g. MXene)
Stability in air	Y	Y	Y	Some 2D (e.g. phosphorene has limited stability in air)
Electronic properties	Generally small conductivity, but depends on the degree of oxidation.	Conductive	Highly conductive	Metallic, semiconducting or insulating, depending on the 2D material
NIR Absorption	Good	Very good	Good	Depends on the 2D material
Photoluminescence	Y, weak	N	N	some of the 2D materials (e.g. TMDs) show photoluminescence
Biocompatibility	Depends on the way of production, physicochemical properties and doses			
